# Supplementary figures and images for: Bacteriologically confirmed extrapulmonary tuberculosis and the associated risk factors among extrapulmonary tuberculosis suspected patients in Ethiopia: A systematic review and meta-analysis
Source: PLoS One. 2022 Nov 23;17(11):e0276701. doi: 10.1371/journal.pone.0276701 (PMC9683558; doi:10.1371/journal.pone.0276701)

Literature search strategy

ScienceDirect


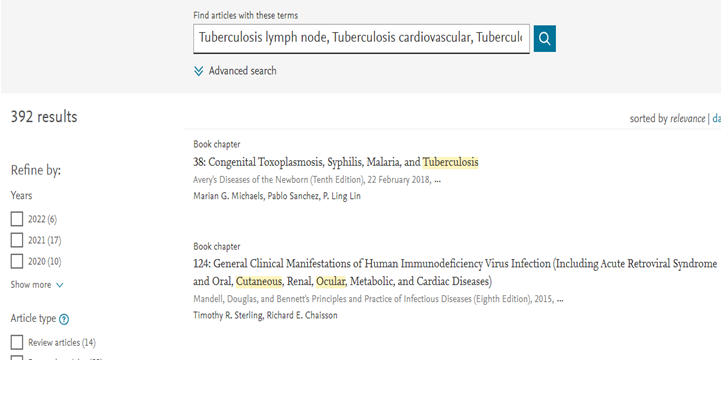


PubMed


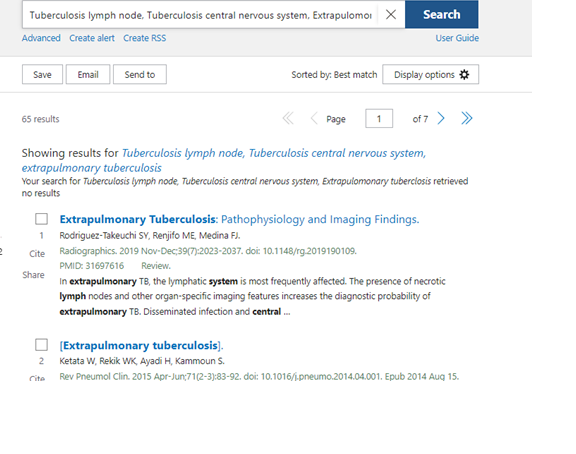


Google Scholar


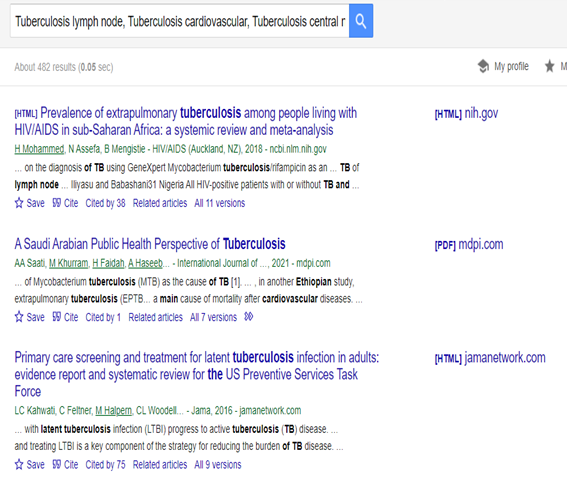

Supplement: S2 File — (DOCX) [file pone.0276701.s002.docx]
