# Supplementary material for: Bacteriologically confirmed extrapulmonary tuberculosis and the associated risk factors among extrapulmonary tuberculosis suspected patients in Ethiopia: A systematic review and meta-analysis
Source: PLoS One. 2022 Nov 23;17(11):e0276701. doi: 10.1371/journal.pone.0276701 (PMC9683558; doi:10.1371/journal.pone.0276701)
Supplement: S4 File — (DOCX) [file pone.0276701.s004.docx]

Supplementary Material. Newcastle-Ottawa quality assessment scale for cross sectional studies

| Author, year | Q1 | | | Q2 | | | Q3 | | | Q4 | | | Q5 | | | Q6 | | | Q7 | | | Q8 | | | Q9 | | | | Overall quality result | |
| --- | --- | --- | --- | --- | --- | --- | --- | --- | --- | --- | --- | --- | --- | --- | --- | --- | --- | --- | --- | --- | --- | --- | --- | --- | --- | --- | --- | --- | --- | --- |
|  | Yes | No | Can’t tell | Yes | No | Can’t tell | Yes | No | Can’t tell | Yes | No | Can’t tell | Yes | No | Can’t tell | Yes | No | Can’t tell | Yes | No | Can’t tell | Yes | No | Can’t tell | Yes | No | Can’t tell |  | |  |
| Yassin et al 2003 | √ |  |  | √ |  |  | √ |  |  |  |  | √ | √ |  |  | √ |  |  | √ |  |  | √ |  |  | √ |  |  | 90% | |  |
| Iwnetu et al 2009 | √ |  |  | √ |  |  | √ |  |  | √ |  |  |  | √ |  |  | √ |  | √ |  |  | √ |  |  | √ |  |  | 80% | |  |
| Derese et al 2012 | √ |  |  | √ |  |  | √ |  |  | √ |  |  |  | √ |  |  | √ |  | √ |  |  | √ |  |  | √ |  |  | 80% | |  |
| Biadglegne et al 2013 | √ |  |  | √ |  |  | √ |  |  | √ |  |  |  | √ |  |  | √ |  | √ |  |  | √ |  |  | √ |  |  | 80% | |  |
| Zenebe et al 2013 | √ |  |  | √ |  |  | √ |  |  | √ |  |  |  | √ |  |  | √ |  | √ |  |  | √ |  |  | √ |  |  | 80% | |  |
| Garedew et al 2013 | √ |  |  | √ |  |  | √ |  |  | √ |  |  |  | √ |  |  | √ |  | √ |  |  | √ |  |  | √ |  |  | 80% | |  |
| Abdissa et el 2014 | √ |  |  | √ |  |  | √ |  |  |  | √ |  |  | √ |  |  | √ |  | √ |  |  | √ |  |  | √ |  |  | 70% | |  |
| Birhanu et al 2014 | √ |  |  | √ |  |  | √ |  |  |  |  | √ | √ |  |  | √ |  |  | √ |  |  | √ |  |  | √ |  |  | 90% | |  |
| Berg et al 2015 | √ |  |  | √ |  |  | √ |  |  |  | √ |  |  | √ |  |  | √ |  | √ |  |  | √ |  |  | √ |  |  | 70% | |  |
| Tadesse et al 2015 | √ |  |  | √ |  |  | √ |  |  | √ |  |  |  | √ |  |  | √ |  | √ |  |  | √ |  |  | √ |  |  | 80% | |  |
| Korma et al 2015 | √ |  |  | √ |  |  | √ |  |  | √ |  |  |  | √ |  |  | √ |  | √ |  |  | √ |  |  | √ |  |  | 80% | |  |
| Abdissa et al 2015 | √ |  |  | √ |  |  | √ |  |  | √ |  |  |  | √ |  |  | √ |  | √ |  |  | √ |  |  | √ |  |  | 80% | |  |
| Fanosie et al 2016 | √ |  |  | √ |  |  | √ |  |  |  |  | √ | √ |  |  | √ |  |  | √ |  |  | √ |  |  | √ |  |  | 90% | |  |
| Zewdie et al 2016 | √ |  |  | √ |  |  | √ |  |  |  |  | √ | √ |  |  | √ |  |  | √ |  |  | √ |  |  | √ |  |  | 90% | |  |
| Mulu et al 2017 | √ |  |  | √ |  |  | √ |  |  |  |  | √ | √ |  |  | √ |  |  | √ |  |  | √ |  |  | √ |  |  | 90% | |  |
| Metaferia et al 2018 | √ |  |  | √ |  |  | √ |  |  | √ |  |  |  | √ |  |  | √ |  | √ |  |  | √ |  |  | √ |  |  | 80% | |  |
| Tadesse et al 2018 | √ |  |  | √ |  |  | √ |  |  | √ |  |  |  | √ |  |  | √ |  | √ |  |  | √ |  |  | √ |  |  | 80% | |  |
| Fantahun et al 2019 | √ |  |  | √ |  |  | √ |  |  |  | √ |  |  | √ |  |  | √ |  | √ |  |  | √ |  |  | √ |  |  | 70% | |  |
| Tedla et al 2019 | √ |  |  | √ |  |  | √ |  |  |  | √ |  |  | √ |  |  | √ |  | √ |  |  | √ |  |  | √ |  |  | 70% | |  |
| Assefa et al 2021 | √ |  |  | √ |  |  | √ |  |  |  |  | √ | √ |  |  | √ |  |  | √ |  |  | √ |  |  | √ |  |  | 90% | |  |

****Y=yes, N=nor, NA=not applicable, <60%=low, 60-80%=medium, >80%=high quality***
