# Supplementary material for: Bacteriologically confirmed extrapulmonary tuberculosis and the associated risk factors among extrapulmonary tuberculosis suspected patients in Ethiopia: A systematic review and meta-analysis
Source: PLoS One. 2022 Nov 23;17(11):e0276701. doi: 10.1371/journal.pone.0276701 (PMC9683558; doi:10.1371/journal.pone.0276701)
Supplement: Attachment — Submitted filename: PONE-D-22-00257_R1_annotated.pdf [file pone.0276701.s007.pdf]

PLOS ONE

Bacteriologically confirmed extrapulmonary tuberculosis and the associated risk factors among extrapulmonary tuberculosis suspected patients in Ethiopia: A systematic review and meta-analysis

--Manuscript Draft--

**Manuscript Number:**	PONE-D-22-00257R1
**Article Type:**	Research Article
**Full Title:**	Bacteriologically confirmed extrapulmonary tuberculosis and the associated risk factors among extrapulmonary tuberculosis suspected patients in Ethiopia: A systematic review and meta-analysis
**Short Title:**	Bacteriologically confirmed of EPTB and the associated risk factors: A systematic review and meta-analysis
**Corresponding Author:**	Getu Diriba, MSc<br>Ethiopian Public Health Institute<br>Addis Ababa, ETHIOPIA
**Keywords:**	Prevalence, Prevalence, Pulmonary Tuberculosis, Extrapulmonary Tuberculosis, Risk factors
**Abstract:**	Background: The actual burden of bacteriologically confirmed extrapulmonary tuberculosis (EPTB) and risk factors in Ethiopia is not well known due to the lack of a strong surveillance system in Ethiopia. Thus, this study was conducted to estimate the pooled prevalence of bacteriologically confirmed EPTB and the associated risk factors among persons suspected to have non-respiratory tuberculosis /EPTB/ in Ethiopia.<br>Methods: A systematic review and meta-analysis of published studies reporting the prevalence of EPTB from searched electronic databases; Science Direct, PubMed, and Google Scholar estimated spread across the research periods, nationally, and in different areas, using a random-effects model. We used I 2 to analyze heterogeneity in the reported prevalence of bacteriologically confirmed extrapulmonary tuberculosis.<br>Results: After reviewing 938 research articles, 20 studies (19 cross-sectional and 1 retrospective) from 2003 to 2021 were included in the final analyses. The pooled prevalence of bacteriologically confirmed EPTB was 43% (95%CI; 0.34–0.52, I 2 = 98.45%). The asymmetry of the funnel plot revealed the presence of publication bias. Specifically the pooled prevalence of bacteriologically confirmed EPTB based on smear microscopy, Xpert MTB/RIF assay, and culture were 22% (95%CI; 0.13–0.30, I 2 = 98.56%), 39% (95%CI; 0.23–0.54, I 2 = 98.73%) and 49% (95%CI; 0.41–0.57, I 2 = 96.43%) respectively. In this study, a history of previous pulmonary tuberculosis (PTB) contact with PTB patients, contact with live animals, consumption of raw milk, HIV-positive, male, and lower monthly income, were found to be independently associated with EPTB.<br>Conclusion: Ethiopia has a high rate of bacteriologically confirmed EPTB. A history of previous PTB, being HIV-positive and having contact with PTB patients were all associated with EPTB in the majority of studies. Strengthening laboratory services for EPTB diagnosis should be given priority in order to diagnose EPTB cases as early as possible.
**Order of Authors:**	Getu Diriba
	Ayinalem Alemu
	Kirubel Eshetu
	Bazezew Yenew
	Dinka Fikadu Gamtesa
	Habteyes Hailu Tola
**Response to Reviewers:**	Ref: PONE-D-22-00257<br>Bacteriologically confirmed extrapulmonary tuberculosis and the associated risk factors among extrapulmonary tuberculosis suspected patients in Ethiopia: A systematic review and meta-analysis

Powered by Editorial Manager® and ProduXion Manager® from Aries Systems Corporation

Dear Editor,
Thank you for your informative comments and thoughtful comments on our manuscript. We have addressed your and the reviewers' comments one by one. We also appreciate the reviewers for their critical observation and informative comments which radically improved our manuscript quality. We have provided point-by-point responses to the reviewers' comments below. Also, we would like to inform you that we have used a hyperlink to indicate where we made changes in the previous version of the manuscript based on the reviewers' comments.

Journal Requirements:
When submitting your revision, we need you to address these additional requirements.
1. Please ensure that your manuscript meets PLOS ONE's style requirements, including those for file naming. The PLOS ONE style templates can be found at https://journals.plos.org/plosone/s/file?id=wjVg/PLOSOne_formatting_sample_main_body.pdf and
https://journals.plos.org/plosone/s/file?id=ba62/PLOSOne_formatting_sample_title_authors_affiliations.pdf
Response: Thank you for the comment. The manuscript was prepared according to PLOS ONE's style requirements.
2. Please provide additional details regarding participant consent. In the ethics statement in the Methods and online submission information, please ensure that you have specified (1) whether consent was informed and (2) what type you obtained (for instance, written or verbal, and if verbal, how it was documented and witnessed). If your study included minors, state whether you obtained consent from parents or guardians. If the need for consent was waived by the ethics committee, please include this information.
If you are reporting a retrospective study of medical records or archived samples, please ensure that you have discussed whether all data were fully anonymized before you accessed them and/or whether the IRB or ethics committee waived the requirement for informed consent. If patients provided informed written consent to have data from their medical records used in research, please include this information.
Response: Thank you for the valuable comment. In the revised manuscript we included a sentence about ethical consideration “Since this study is based on previously published articles ethical approval is not applicable”.
3. Thank you for stating the following financial disclosure:
“The funders had no role in study design, data collection and analysis, decision to publish, or preparation of the manuscript.”
At this time, please address the following queries:
a) Please clarify the sources of funding (financial or material support) for your study. List the grants or organizations that supported your study, including funding received from your institution.
b) State what role the funders took in the study. If the funders had no role in your study, please state: “The funders had no role in study design, data collection and analysis, decision to publish, or preparation of the manuscript.”
c) If any authors received a salary from any of your funders, please state which authors and which funders.
d) If you did not receive any funding for this study, please state: “The authors received no specific funding for this work.”
Please include your amended statements within your cover letter; we will change the online submission form on your behalf.
Response: Thank you. This work did not receive any funding for this study and we have already stated it in the first submission before the reference section “The author(s) received no specific funding for this work”
4. We note that you have stated that you will provide repository information for your data at acceptance. Should your manuscript be accepted for publication, we will hold it until you provide the relevant accession numbers or DOIs necessary to access your data. If you wish to make changes to your Data Availability statement, please describe these changes in your cover letter and we will update your Data Availability statement to reflect the information you provide.
Response: Thank you. All the important information is available within the manuscript and its supplementary files.
5. Please ensure that you refer to Figure 5 in your text as, if accepted, production will need this reference to link the reader to the figure.

Response: Thank you for your critical observation and comment; we revised as per the suggestion.
Additional Editor Comments:
Both reviewers felt that the paper had merit. Both did however identify concerns with the methodology. Specifically, there was a concern that the denominator of the study (all PTB cases) was not realistic and undefined and 1 reviewer felt strongly that the review question needed to be tightened. There were also concerns with the construction of the forest plots which reviewer 1 felt should be ordered from low to high. Both reviewers highlighted a number of small stylistic errors which should also be corrected.
Response: Thank you for your suggestions and constructive comments. We have addressed your comments across the manuscript methodology. All the forest plots are arranged from low to high. Also, all technical errors have been corrected.
Review Comments to the Author
Reviewer #1
22 lack of a strong…
Response: Thank you, we revised it accordingly.
24 insert full stop
Response: Thank you, we corrected it as per the suggestion.
27 spread across
Response: Thank you for your suggestion. We have corrected it.
29 insert full stop
Response: Thank you for your suggestion. We inserted a full stop in the revised manuscript.
31 “majority”: what is the percentage?
Response: Thank you for the question. At this stage, we revised the result section of the abstract.
31 substitute “articles” for “research”
Response: Thank you for your suggestion. We revised it accordingly.
32 variations
Response: Thank you. We have corrected it.
33 “medium/high risk”: the funnel plot in fig 3 for the 20 papers shows 10 papers above the 95% upper limit and 5 below the 95% lower limit and only 5 within the CI. More discussion is required for your assessment. Is this justified by the standard error of the effect size on the y-axis?
Response: Thank you for the valuable comment. Now we included a detailed paragraph in the discussion section that described it.
34 Delete “having”. PTB must be in full here, that is, “pulmonary tuberculosis”
Response: Thank you for the valuable comment. Now, defined it in full first and then we used the abbreviation.
35 Delete “being”
Response: Thank you. We have deleted the comment
41 Keywords:… “Pulmonary Tuberculosis” for Tuberculosis…
Response: Thank you for the suggestion. We revised it.
49 Use “EPTB”; this has already been defined
Response: Thank you. In the current version, we used EPTB.
54 EPTB
Response: Thank you for your suggestion. We have corrected it.
55 15%
Response: Thank you for your comment. We have added the % according to the comment
61 Delete “and”
Response: Thank you for your suggestion. We have deleted it.
62 Delete extra space after “resource- “
Response: Thank you. We have deleted the extra space.
64 turnaround times
Response: Thank you for your suggestion. We corrected it accordingly.
70 “had started” for “have been started”
Response: Thank you for your suggestion. We revised it as per the given suggestion.
72 (11) for [11]
Response: Thank you for the suggestion. We revised the format for the whole referencing style based on the Plos One journal requirements.
76 et seq. Remove end full stops in numbering, for example, 2. and 2.1. should be 2 and 2.1

Powered by Editorial Manager® and ProduXion Manager® from Aries Systems Corporation

	Response: Thank you for your suggestion. In the revised version, we have removed the numbers in the entire manuscript according to the Journal’s criteria.<br>86 Delete “the”<br>Response: Thank you for your suggestion. We deleted it.<br>88 Delete “language”<br>Response: Thank you for your suggestion. We deleted it.<br>93 “using a Microsoft Excel worksheet” should be “Microsoft Excel® 2016” (or whatever version was used)<br>Response: Thank you for your suggestion. We have revised it accordingly.<br>96 “resolved inconsistencies that arose”<br>Response: Thank you for your suggestion. We revised it as per the suggestion<br>99-102 The WHO definition of a positive test result was applied. This states that a positive diagnostic test result using smear microscopy, culture, Xpert MTM/RIF or nucleic acid amplification test is a bacteriological confirmation of an EPTB infection.<br>Response: Thank you for the valuable comment and suggestion. We have revised it as per the given suggestion.<br>104 Delete “included”<br>Response: Thank you, we have deleted it.<br>105-107 Persistent disagreements indicate a lack of clarity in applying the Newcastle-Ottawa scale. Should the scale be modified and would different interpretations be applied by researchers on similar studies. The nature of the disagreements and outcomes of using alternative interpretations should be discussed especially in light of the small sample of 20 papers<br>Response: Thank you; we have addressed this according to the comment<br>112 STATA® 14.2 StataCorp LLC, Texas, USA<br>Response: Thank you, we revised it accordingly.<br>113 a 95% confidence interval. Random-effects<br>Response: We have changed according to the comment<br>114 The ‘metaprop’ command in STATA was used.<br>Response: Thank you, we revised it accordingly.<br>115-116 A forest plot shows distributional information not prevalence<br>Response: Thank you, it is revised accordingly.<br>119-127 This requires the editing out of excess detail in light of the PRISMA diagram. The PRISMA diagram is important and sets out clearly the process in reducing the 938 articles to 20 used in this study 123 The 36 non-Ethiopian papers should either be included as controls in the forest plots or in separate forest plots. These could indicate possible biases, inconsistencies or differences in interpretation of data in Ethiopian papers (for example see disagreements in 105-107 above). This applies to funnel plots as well<br>Response: Thank you, we revised it as per the suggestion.<br>135 Delete “was”<br>Response: We have deleted it.<br>142 Delete “another”<br>Response: We have deleted it.<br>Page 8 Some hyphens missing in “cross-sectional”. Associated factors column requires some editing (spaces and spacing of commas)<br>Response: Thank you, we revised it accordingly.<br>148 The frequency of EPFB varied widely over the 20 studies. The prevalence ranged from 9% (21) to 78% (32). See also general comment below on superscript of paper reference.<br>Response: Thank you for the comment. The numbers in the bracket are the references, now we revised the referencing based on the journal’s criteria or using [ ].<br>150 random-effects<br>Response: We have changed according to the comment<br>157 using a random-effects model<br>Response: We have corrected accordingly.<br>157 “Effect size” should appear in full in the x-axis label<br>Response: Thank you. We have addressed the comment<br>158 “twenty studies”, delete used.<br>Response: We have deleted it.<br>158-159 Standard error of effect size should appear in full on the y-axis<br>Response: Thank you for the suggestion, we corrected it accordingly.<br>160 The subgroups of the 20 studies should be shown in the PRISMA diagram to ensure completeness and intelligibility

Powered by Editorial Manager® and ProduXion Manager® from Aries Systems Corporation

	Response: Thank you for the suggestion. We sub-grouped the included studies based on the laboratory method used, and we described it in the flow diagram and early in the result section.<br>170 Pooled proportion of culture-positive EPTB using random-effects model<br>Response: Thank you, we revised it as per the suggestion.<br>172 Same style as 170<br>Response: We have changed according to the comment<br>174 Same style as 170<br>Response: We have changed according to the comment<br>184 Funnel plot of subgroup of 15 of the 20 selected studies<br>Response: We have changed according to the comment<br>184 Standard error of effect size and effect size should appear in the axis labels<br>Response: Thank you for the valuable comment; we revised accordingly per the suggestion. and effect size according to the comment<br>186 Same style as 184<br>Response: Thank you, we revised it accordingly.<br>188 Same style as 184<br>Response: Thank you, we revised it accordingly.<br>192 A history of PTB infection<br>Response: Thank you, we corrected it.<br>200 Delete “of”<br>Response: Thank you we have deleted it.<br>196 HIV should be HIV-1<br>Response: Thank you for the suggestion, we revised it accordingly.<br>204 A history of PTB infection ("history" implies previous history)<br>Response: We have corrected per the suggestion.<br>210 … similar to that…<br>Response: We revised it.<br>214 “systemic” should be “systematic”<br>Response: Thank you, now it is corrected.<br>217 HIV-1<br>Response: Thank you for the suggestion, we revised it accordingly.<br>218 …risk factors associated with EPTB infection…<br>Response: Thank you, we revised it as per the suggestion.<br>219 HIV-1<br>Response: Thank you, now it is revised accordingly.<br>220 … EPTB patients were HIV-1 infected,…<br>Response: Thank you. We revised as per the suggestion.<br>223 TB-HIV-1 co-infection<br>Response: Thank you. We revised it accordingly.<br>227 … with a history of… (“history” implies previous history)<br>Response: We have revised it accordingly.<br>235 Firstly,<br>Response: Thank you, we revised it.<br>237 Secondly,<br>Response: Thank you, we revised it.<br>238 Statistical power and its reduction has not been discussed in the paper. What is the estimate of the reduction and has this invalidated the paper? (Does statistical power here refer to I^2, p-value or items outside the 95% pseudo-confidence gradients in the funnel plots? 238 “poor quality of several studies” – I am not sure this has been sufficiently discussed (if at all)<br>Response: Thank you for the valuable comment. Now, we revised the limitation of the study as follows “In the end, the current study had its limitations. Firstly, the degree of EPTB prevalence in many parts of the country has yet to be addressed, making it impossible to conclude the true burden of EPTB in Ethiopia. Secondly, the observed publication bias that could be due to the differences in the laboratory methods might underestimate the estimated prevalence. Thirdly, there is high heterogeneity among studies that might affect the true estimates. However, the findings are still significant, because the rising rate of EPTB patients in the general population is concerning”<br>244 HIV-1<br>Response: We revised it accordingly.<br>General: number references to papers should preferably be shown as superscripts to avoid with number in the paper. For example, line 49, …region (8%) (3) should be …region (8%)3 (3 as superscript)

	Response: Thank you for the valuable comment and suggestion. Now we revised all the references according to the PloS One guideline, Such that we used the square brackets “[ ]”.<br>References: Journal references should be italicised. Latinisms like et.al. should be italicized<br>Response: Thank you, we revised as per the suggestion.<br>Figure 1: PRISMA diagram should show the subgroups budding off the “Final analysis” box. Reference to this in the main body of the paper will simplify some of the wording in the main body<br>Response: Thank you for the suggestion, as we have stated in the previous response we revised the flow diagram according to the PRISMA guideline.<br>Figure 2: Forest plot should be sorted by ES and show ranking from lowest to highest ES. Metaferia et. al. would be at the bottom and Iwnetu et.al. at the top<br>Response: Thank you for the comment, we revised it accordingly.<br>Figure 3: Axis-labels to be described in full<br>Response: We have described in full according to the comment<br>Figure 4: Sort the publications as in figure 2<br>Response: Thank you, we revised it accordingly.<br>Figure 5: Sort the publications as in figure 2<br>Response: Thank you, we revised it accordingly.<br>Figure 6: Sort the publications as in figure 2<br>Response: Thank you, we revised it accordingly.<br>Figures 7, 8 and 9 – same as in figure 3<br>Response: Thank you, we revised it accordingly.<br>Reviewer #2:<br>This is a well written article, and findings will be helpful to Ethiopian health care workers, and others working in a LMIC/African context.<br>However, I have a difficulty with the foundational methodology (meta-analysis) of the study.<br>o It is not stated what the research questions for the descriptive studies of EPTB which were included in the meta-analysis were.<br>o Studies which were included could have identified EPTB as a subset of all PTB, or they may have described EPTB alone, from persons with clinical criteria matching only those in whom EPTB is suspected.<br>o Without specifying which approach the authors of this meta-analysis wanted de novo, the selection criteria results in a wide range of approaches to EPTB diagnosis being selected, which will lead to enormous heterogeneity, and lack of meaningful comparison.<br>Before conducting a meta-analysis, it is critical to given to formulate the review question clearly. I think the review question ‘to investigate the prevalence of EPTB in Ethiopia’ is too broad. It will be helpful to say ‘Investigate the prevalence of EPTB amongst persons with non-pulmonary TB’. The results are then clearly applicable to persons in whom PTB has been excluded.<br><br>As the current aim stands, it implies that that the aim is to identify the burden of TB amongst all TB cases in Ethiopia. This begs the question of a denominator. Assessing the prevalence of EPTB requires that one knows the total burden of TB. How is it possible to assess the prevalence by identifying only studies that described EPTB? These studies would have identified persons who were suspected of EPTB as the starting point. Equally, an ascertainment of risk factors can only be done by comparison with non-EPTB groups.<br>Without narrowing the research question, it is not meaningful to present or interpret Forest plots.<br>Response: Thank you for the pertinent comments and valuable suggestions. This study is designed to investigate/estimate/ the prevalence of bacteriologically confirmed EPTB among individuals who are presumptive to have EPTB. Thus, the denominator is the number of EPTB or non-respiratory TB presumptive individuals, while the numerator is the number of bacteriologically confirmed EPTB presumptive individuals. Now we described in detail the research questions and the objectives in the revised manuscript. We revised the abstract section, the objectives at the end of the introduction section, and the first paragraph in the discussion section.<br>Other minor comments<br>A small consideration re methodology, search strategy – are there articles published by Ethiopians in literature that would not be listed in pubmed, Science Direct or on google

	scholar?<br>Response: Thank you very much for your informative comments. The majority of the articles published from Ethiopia are open access, because Ethiopia is among the low-income countries which waived article processing charges. Thus, all open access articles can be accessed through Google Scholar research. In addition, Since we cannot freely access other databases in Ethiopia we could not search them. To address this comment we have added a sentence that indicates as our search is not comprehensive in limitation.<br>Line 49 Extrapulmonary Tuberculosis – 'tuberculosis' should be lower case.<br>Response: Thank you for the comment, we revised it accordingly.<br>Line 64 Turnaround Time – T should be lower case,<br>Response: Thank you, we revised it as per the suggestion.<br>Line 70 Anti-TB medicine – rather 'anti-tuberculosis therapy'<br>Response: Thank you, we revised it accordingly.<br>Line 108 The NewCastle Ottowa quality assessment scale is a checklist for cohort and case control studies. What motivated the use of this scale, over and above the PRISMA guidelines which are sufficient for meta-analyses and systematic reviews.<br>Response: Thank you for the valuable question. This systematic review and meta-analysis were conducted following the PRISMA guidelines. We used the Newcastle-Ottawa quality assessment checklist to assess the quality of individuals studies based on the questions available in the checklist as the Joanna Briggs critical appraisal tool. Since all studies included in the current study are cross-sectional studies we have used the Newcastle-Ottawa quality assessment scale adapted for cross-sectional studies.<br><br>Yours, Sincerely<br>Getu Diriba
**Additional Information:**	
**Question**	**Response**
**Financial Disclosure**<br><br>Enter a financial disclosure statement that describes the sources of funding for the work included in this submission. Review the submission guidelines for detailed requirements. View published research articles from *PLOS ONE* for specific examples.<br><br>This statement is required for submission and **will appear in the published article** if the submission is accepted. Please make sure it is accurate.	The author(s) received no specific funding for this work.

**Unfunded studies**<br>Enter: *The author(s) received no specific funding for this work.*<br><br>**Funded studies**<br>Enter a statement with the following details:<br>• Initials of the authors who received each award<br>• Grant numbers awarded to each author<br>• The full name of each funder<br>• URL of each funder website<br>• Did the sponsors or funders play any role in the study design, data collection and analysis, decision to publish, or preparation of the manuscript?<br>• **NO** - Include this sentence at the end of your statement: *The funders had no role in study design, data collection and analysis, decision to publish, or preparation of the manuscript.*<br>• **YES** - Specify the role(s) played.<br><br>* typeset	
**Competing Interests**<br><br>Use the instructions below to enter a competing interest statement for this submission. On behalf of all authors, disclose any competing interests that could be perceived to bias this work—acknowledging all financial support and any other relevant financial or non-financial competing interests.<br><br>This statement is required for submission and **will appear in the published article** if the submission is accepted. Please make sure it is accurate and that any funding sources listed in your Funding Information later in the submission form are also declared in your Financial Disclosure statement.<br><br>View published research articles from *PLOS ONE* for specific examples.	The authors have declared that no competing interests exist.

**NO authors have competing interests**<br><br>Enter: *The authors have declared that no competing interests exist.*<br><br>**Authors with competing interests**<br><br>Enter competing interest details beginning with this statement:<br><br>*I have read the journal's policy and the authors of this manuscript have the following competing interests: [insert competing interests here]*<br><br>* typeset	
**Ethics Statement**<br><br>Enter an ethics statement for this submission. This statement is required if the study involved:<br><br>• Human participants<br>• Human specimens or tissue<br>• Vertebrate animals or cephalopods<br>• Vertebrate embryos or tissues<br>• Field research<br><br>Write "N/A" if the submission does not require an ethics statement.<br><br>General guidance is provided below. Consult the submission guidelines for detailed instructions. **Make sure that all information entered here is included in the Methods section of the manuscript.**	Since this study is based on previously published articles ethical approval is not applicable.

Powered by Editorial Manager® and ProduXion Manager® from Aries Systems Corporation

**Format for specific study types**<br><br>**Human Subject Research (involving human participants and/or tissue)**<br>• Give the name of the institutional review board or ethics committee that approved the study<br>• Include the approval number and/or a statement indicating approval of this research<br>• Indicate the form of consent obtained (written/oral) or the reason that consent was not obtained (e.g. the data were analyzed anonymously)<br><br>**Animal Research (involving vertebrate animals, embryos or tissues)**<br>• Provide the name of the Institutional Animal Care and Use Committee (IACUC) or other relevant ethics board that reviewed the study protocol, and indicate whether they approved this research or granted a formal waiver of ethical approval<br>• Include an approval number if one was obtained<br>• If the study involved *non-human primates*, add *additional details* about animal welfare and steps taken to ameliorate suffering<br>• If anesthesia, euthanasia, or any kind of animal sacrifice is part of the study, include briefly which substances and/or methods were applied<br><br>**Field Research**<br><br>Include the following details if this study involves the collection of plant, animal, or other materials from a natural setting:<br>• Field permit number<br>• Name of the institution or relevant body that granted permission	
**Data Availability**<br><br>Authors are required to make all data underlying the findings described fully available, without restriction, and from the time of publication. PLOS allows rare exceptions to address legal and ethical concerns. See the PLOS Data Policy and FAQ for detailed information.	Yes - all data are fully available without restriction

A Data Availability Statement describing where the data can be found is required at submission. Your answers to this question constitute the Data Availability Statement and **will be published in the article**, if accepted.<br><br>**Important:** Stating 'data available on request from the author' is not sufficient. If your data are only available upon request, select 'No' for the first question and explain your exceptional situation in the text box.<br><br>Do the authors confirm that all data underlying the findings described in their manuscript are fully available without restriction?	
**Describe where the data may be found in full sentences. If you are copying our sample text, replace any instances of XXX with the appropriate details.**<br><br>• If the data are **held or will be held in a public repository**, include URLs, accession numbers or DOIs. If this information will only be available after acceptance, indicate this by ticking the box below. For example: *All XXX files are available from the XXX database (accession number(s) XXX, XXX.*).<br>• If the data are all contained **within the manuscript and/or Supporting Information files**, enter the following: *All relevant data are within the manuscript and its Supporting Information files.*<br>• If neither of these applies but you are able to provide **details of access elsewhere**, with or without limitations, please do so. For example:<br><br>*Data cannot be shared publicly because of [XXX]. Data are available from the XXX Institutional Data Access / Ethics Committee (contact via XXX) for researchers who meet the criteria for access to confidential data.*<br><br>*The data underlying the results presented in the study are available from (include the name of the third party*	All the important information is available within the manuscript and its supplementary files.

and contact information or URL).<br>• This text is appropriate if the data are owned by a third party and authors do not have permission to share the data.<br><br>* typeset	
Additional data availability information:	Tick here if the URLs/accession numbers/DOIs will be available only after acceptance of the manuscript for publication so that we can ensure their inclusion before publication.

Powered by Editorial Manager® and ProduXion Manager® from Aries Systems Corporation

Manuscript
Click here to access/download;Manuscript;Revised Manuscript without Track Changes.docx

Bacteriologically confirmed extrapulmonary tuberculosis and the associated risk factors among extrapulmonary tuberculosis suspected patients in Ethiopia: A systematic review and meta-analysis

Getu Diriba[1*], Ayinalem Alemu[1,2], Kirubel Eshetu[3], Bazezew Yenew[1], Dinka Fikadu Gamtesa[1], Habteyes Hailu Tola[1]

[1]Ethiopian Public Health Institute, Addis Ababa, Ethiopia

[2]Aklilu Lemma Institute of Pathobiology, Addis Ababa University

[3]USAID Eliminate TB Project, Management Sciences for Health, Addis Ababa, Ethiopia

[*]Corresponding author Getu Diriba

Ethiopian Public Health Institute

Phone: +251913828019. Fax: +2510112780431

P.O.Box 1242, Addis Ababa, Ethiopia

Email: getud2020@gmail.com

Abstract

Background: The actual burden of bacteriologically confirmed extrapulmonary tuberculosis (EPTB) and risk factors in Ethiopia is not well known due to the lack of a strong surveillance system in Ethiopia. Thus, this study was conducted to estimate the pooled prevalence of bacteriologically confirmed EPTB and the associated risk factors among persons suspected to have non-respiratory tuberculosis /EPTB/ in Ethiopia.

Methods: A systematic review and meta-analysis of published studies reporting the prevalence of EPTB from searched electronic databases; Science Direct, PubMed, and Google Scholar estimated spread across the research periods, nationally, and in different areas, using a random-effects model. We used I^2 to analyze heterogeneity in the reported prevalence of bacteriologically confirmed extrapulmonary tuberculosis.

Results: After reviewing 938 research articles, 20 studies (19 cross-sectional and 1 retrospective) from 2003 to 2021 were included in the final analyses. The pooled prevalence of bacteriologically confirmed EPTB was 43% (95%CI; 0.34–0.52, I^2 = 98.45%). The asymmetry of the funnel plot revealed the presence of publication bias. Specifically the pooled prevalence of bacteriologically confirmed EPTB based on smear microscopy, Xpert MTB/RIF assay, and culture were 22% (95%CI; 0.13–0.30, I^2 = 98.56%), 39% (95%CI; 0.23–0.54, I^2 = 98.73%) and 49% (95%CI; 0.41–0.57, I^2 = 96.43%) respectively. In this study, a history of previous pulmonary tuberculosis (PTB) contact with PTB patients, contact with live animals, consumption of raw milk, HIV-positive, male, and lower monthly income, were found to be independently associated with EPTB.

Conclusion: Ethiopia has a high rate of bacteriologically confirmed EPTB. A history of previous PTB, being HIV-positive and having contact with PTB patients were all associated with EPTB in the majority of studies. Strengthening laboratory services for EPTB diagnosis should be given priority in order to diagnose EPTB cases as early as possible.

Keywords: Prevalence, Pulmonary Tuberculosis, Extrapulmonary Tuberculosis, Risk factors

Introduction

Pulmonary tuberculosis (PTB), which affects the lungs, accounts for 85% of all reported tuberculosis cases globally [1]. However, extrapulmonary tuberculosis (EPTB), which affects parts of the body other than the lung is becoming a major concern in TB prevention and control efforts [2]. The prevalence of EPTB among notified TB cases in the African region in the 2018 global report was 16%, which is the second-highest next to the Eastern Mediterranean region (24%), which is more than the global prevalence of EPTB (15%). The lowest prevalence was reported in the Western Pacific region (8%) [3]. EPTB is assumed to be produced by the spread of bacteria through the bloodstream from a primary focus in the lung, and hence represents a disseminated form of tuberculosis. EPTB most commonly affects the lymph nodes, abdomen, pleura, bones, and meninges, but the prevalence varies with age, sex, and geographic location [4].

Extrapulmonary TB remains a critical concern both in developing and developed countries. EPTB accounts for 15% to 30% of all tuberculosis cases [5, 6]. In persons with HIV/AIDS and other immunocompromised states, it is a prevalent opportunistic infection [7]. In Ethiopia, there is a scarcity of evidence on bacteriological diagnosis and evaluation of EPTB.

Extrapulmonary tuberculosis is difficult to diagnose for a variety of reasons. Many types of EPTB necessitate invasive diagnostic sampling, which can be dangerous to the patient and expensive. Because most types of EPTB are paucibacillary (TB disease caused by a limited number of bacteria), detection by smear microscopy is less sensitive. This particularly affects resource-limited settings, where the more sensitive methods of mycobacterial culture examination are not widely available. Culture has its own set of drawbacks such as a very long turnaround time and necessitates a well-equipped biosafety laboratory [8]. Molecular methods are a quick and sensitive procedure that only requires a small amount of sample and may be used on killed bacteria; however, they require highly trained technologists and can be expensive [9]. As a result of these challenges, EPTB is frequently diagnosed solely based on clinical suspicion, and many people are given the incorrect diagnosis, resulting in needless TB treatment or poor outcomes from untreated EPTB. Even in tertiary health care facilities, the majority of patients had started anti-tuberculosis therapy without bacteriological evidence. These people were misdiagnosed or received therapy too late, and they overestimated the scale of the problem at the community level [10]. There is a scarcity of data on the bacteriologically confirmed of EPTB among suspected EPTB individuals in Ethiopia. There isn't a comprehensive review and meta-analysis of the current research to uncover evidence to support policymaking. As a result, this study aimed to investigate the prevalence of bacteriologically confirmed EPTB and the associated risk factors amongst persons suspected to have non-respiratory TB /EPTB/ in Ethiopia.

Materials and methods

Search strategy

We systematically searched electronic databases such as MEDLINE (PubMed), Science Direct, and gray literature sources such as Google Scholar and Google for articles published in the English language. We used key terms such as “Tuberculosis lymph node”, “Tuberculosis cardiovascular”, “Tuberculosis central nervous system”, “Tuberculosis cutaneous”, “Tuberculosis endocrine”, “Tuberculosis gastrointestinal”, “Tuberculosis hepatic”, “Tuberculosis ocular”, “Tuberculosis osteoarticular”, “Tuberculosis pleura”, “Tuberculosis splenic”, “Tuberculosis urogenital”, and “Ethiopia” both in MeSH and free text.

Eligibility criteria

We included studies that reported the prevalence of EPTB in Ethiopia. Observational studies, such as cohort (prospective or retrospective) and cross-sectional studies, were included. Studies that were written in English and published before October 26, 2021 (the last date of the searching date), were considered. The studies were included regardless of the diagnostic methods used. The articles without a journal name and/or authors, conference proceedings or presentations, and reviews were excluded from the final analysis.

Data extraction

We created a data extraction sheet using a Microsoft Excel® 2010worksheet. Two independent authors (GD, AA) extracted data including study period, study setting (community or facility-based), study site, test method, sample size, and the number of positive patients. The third author (DF) resolved the inconsistencies that arose between the two authors. To ensure consistency, another co-author (HHT) independently examined the extracted data.

Operational Definition

In this meta-analysis, the WHO definition of a positive test result was applied. This states that a positive diagnostic test result using smear microscopy, culture, Xpert MTM/RIF or nucleic acid amplification test is a bacteriological confirmation of an EPTB infection [11].

Risk of bias assessment and study quality

Two authors (GD and DF) independently assessed the quality of the studies using the Newcastle-Ottawa quality assessment scale adapted for cross-sectional studies [12]. The tool has three components: selection, comparability, and outcome/exposure. The selection part is scored from zero to five stars, and the comparability is scored from zero to two stars. The outcome is scored from zero to three stars. To minimize the subjective interpretation of bias from scoring two reviewers (GD and DF) were assessed the quality of individual studies. Furthermore, when the disagreements that occurred throughout the quality grading process were settled by consulting a third author (AA) [12]. We used I^2 to analyze heterogeneity in reported prevalence [13]. The presence of heterogeneity was defined as an $I^2 \geq 50\%$ [14]. A funnel plot was also used to investigate the presence of publication bias.

Statistical analysis

For statistical analysis, STATA® 14.2 StataCorp LLC, Texas, USA software was employed. We estimated the pooled prevalence of EPTB and a 95% confidence interval using a random-effect meta-analysis model. The 'metaprop' command in STATA 14 (STATA Corporation, College Station, TX, USA) was used to determine the pooled prevalence of EPTB in patients with suspected TB. The distributional information of EPTB was displayed using a forest plot.

Ethical consideration and consent

Since this study is based on previously published articles ethical approval is not applicable.

Result

Study selection

The three electronic databases yielded a total of 938 articles, which were then imported into an Endnote X8 citation manager, and 173 duplicates were removed. Next, 722 articles were screened by title abstract and different outcomes. Around 43 articles that passed the first stage were assessed through a full-text review. During this review, the study subjects, study design, study quality, and outcome were considered. Due to this reason 23 articles were removed. Finally, 20 articles became eligible for data extraction. Regarding the EPTB laboratory diagnostic method used in the selected 20 studies, five studies used culture methods, five studies used culture and smear microscopy and three studies used culture, Xpert MTB/RIF, and smear microscopy, two studies used Xpert MTB/RIF, two studies used smear microscopy, two studies used culture and Xpert MTB/RIF, one study used Xpert MTB/RIF and Smear microscopy. The methodical selection of literature is depicted in Fig 1. The recommended reporting items for systematic reviews and meta-analysis (PRISMA) flow diagram were used to complete the overall screening (Fig 1).

Figure 1: shows the PRISMA flow diagram, which depicts the study screening and selection process.

Characteristics of the studies included in the review

Of the total of 20 articles reviewed, eight studies were undertaken in the Amhara region [15-22], four studies in Addis Ababa [23-26], four in Oromia [27-30], one study in the Southern Nations Nationalities and People Region [31], and the remaining three studies were based on data collected from different regions of the country [32-34]. The data collection period ranges from

1998 [31] to 2020 [26]. The sample size investigated ranged from 90 [18] to 1,198 participants [34]. The majority of the studies were cross-sectional studies, with one retrospective study. Fourteen studies only examined one type of sample, while the remaining six studies reported evaluating various sample types. Regarding diagnostic methods, five studies used smear microscopy and culture methods [26, 29, 32-34], three studies used smear microscopy, culture, and Xpert MTB/RIF assay [15, 25, 28], two studies applied smear microscopy method [16, 31], two studies used the Xpert MTB/RIF assay [20, 22], two studies used the culture and Xpert MTB/RIF assay [19, 30], one study used the Xpert MTB/RIF and microscopy methods [21], and the remaining five studies used the culture method [17, 18, 23, 24, 27] (Table 1).

Table 1: The general characteristics of the studies that were included in the review.

First Author [ref.]	Publication Year	Study Design	Study area	Study Setting	Study time	Sample size	Positive	Type of EPTB	Diagnostic Method	Associated factors
Yassin et al [31]	2003	cross-sectional	Butajira	Facility-based	1998-2000	147	107	Lymph node	Microscopy	
Iwnetu et al [32]	2009	cross-sectional	Addis Ababa, Bar Dar, Diredawa	Facility-based	2004-2005	150	117	Lymph node	Microscopy<br>Culture	
Derese et al [33]	2012	retrospective	Woldia, Butajira, Gonder	Facility-based	2011	134	50	Lymph node	Microscopy<br>Culture	
Biadglegne et al [15]	2013	cross-sectional	Bahirdar, Gondar & Dessie	Facility-based	2012	437	226	Lymph node	Microscopy<br>Culture<br>Xpert MTB/RIF	Retreated, Male , Age < 14, Urban
Zenebe et al [16]	2013	cross-sectional	Gonder	Facility-based	2012	344	34	Lymph node	Microscopy	previous history of PTB, raw milk, monthly less income, TB contact
Garedew et al [17]	2013	cross-sectional	Debre Birhan	Facility-based	2010-2011	98	36	Lymph node	Culture	
Abdissa et el [27]	2014	cross-sectional	Jimma	Facility-based	2012	200	147	Lymph node	Culture	
Birhanu et al [18]	2014	cross-sectional	Dessie	Facility-based	2012-2013	90	32	Lymph node	Culture	

Berg et al [34]	2015	cross-sectional	Gondar, Woldiya, Ghimbi, Fiche, and Butajira, Jinka and Filtu, AA	Facility-based	2006–2010	1198	456	Lymph node	Microscopy Culture	having regular and direct contact with live animals, low education level
Tadesse et al [28]	2015	cross-sectional	Jimma	Facility-based		143	88	Lymph node	Microscopy Culture Xpert MTB/RIF	
Korma et al [23]	2015	cross-sectional	Addis Ababa	Facility-based	2012 to 2013	200	116	pleural, peritoneal and synovial fluids	Culture	
Abdissa et al [29]	2015	cross-sectional	Jimma	Facility-based	2013	144	96	Lymph node	Microscopy Culture	
Fanosie et al [19]	2016	cross-sectional	Gonder	Facility-based	2015	141	37	Peritoneal fluid, CSF, Pleural fluid, lymph node	Culture Xpert MTB/RIF	Adult patients, previous history of contact with known pulmonary TB, HIV positive
Zewdie et al [24]	2016	cross-sectional	Addis Ababa,	Facility-based	2013	206	74	Lymph node	Culture	
Mulu et al [20]	2017	cross-sectional	Debre Markos	Facility-based	2014 to 2015	182	53	Peritoneal, Pus, lymph node, pleural fluid	Xpert MTB/RIF	Retreated ,Male , HIV positive , Age 41-50
Metaferia et al [21]	2018	cross-sectional	Dessie	Facility-based	2017	353	31	Peritoneal fluid, CSF, Pleural fluid, lymph node	Microscopy Xpert MTB/RIF	Previous history of PTB, contact with PTB patients
Tadesse et al [30]	2018	cross-sectional	Jimma	Facility-based	2015-2017	572	242	Lymph node, CSF, pleural, peritoneal, and pericardial fluids.	Culture Xpert MTB/RIF	
Fantahun et al [25]	2019	cross-sectional	Addis Ababa	Facility-based	2015-2016	152	75	Lymph node	Microscopy Culture Xpert MTB/RIF	
Tedla et al [22]	2019	cross-sectional	Dessie	Facility-based	2018	337	92	Peritoneal fluid, CSF, Pleural fluid, lymph node synovial fluid	Xpert MTB/RIF	HIV-positive, previous history of PTB
Assefa et al [26]	2021	cross-sectional	Addis Ababa	Facility-based	2020	211	50	Lymph node	Microscopy Culture	

HIV-human immunodeficiency virus; MTB/RIF-Mycobacterium tuberculosis/ Rifampicin; PTB-pulmonary tuberculosis; TB-Tuberculosis

The pooled prevalence of bacteriologically confirmed EPTB

The frequency of EPTB varied widely over the 20 studies. The prevalence ranged from 9% [21] to 78% [32]. The pooled prevalence of bacteriologically confirmed EPTB was 43% (95%CI; 0.34–0.52, I^2; 98.45%, p-value < 0.01) according to random-effects methodology. The highest EPTB prevalence was reported from Addis Ababa, Bar Dar, and Dire Dawa [32], with a rate of 78%, while the lowest was reported from Dessie [21], with a rate of 9% (Fig 2). The variety of distinct EPTB in Ethiopia is depicted in a funnel plot (Fig 3). The graph depicted studies with fewer participants and events scattered throughout the pooled horizontal estimate, implying a greater influence due to chance.

Figure 2: Pooled proportion of any bacteriological positive of EPTB using a random-effects model.

Figure 3: Funnel plot for the pooled proportion of any bacteriological positive in EPTB studies

Subgroup analysis by diagnostic testing methods

The pooled effect estimate on the prevalence of EPTB positive cases was based on a subgroup analysis of publications comparing culture, smear microscopy, and Xpert MTB/RIF assay methods. There was no heterogeneity among studies conducted in culture, smear microscopy, and Xpert MTB/RIF assay, according to the subgroup analysis performed by a diagnostic test. However, there is still heterogeneity among studies that look at multiple diagnostic tests. In culture, smear microscopy, and Xpert MTB/RIF assay diagnostic methods, the prevalence of pooled effect estimates was 49% (95%CI; 0.41–0.57, I^2 = 96.43%, p-value < 0.01) (see Fig 4), 22% (95%CI; 0.13–0.30, I^2 = 98.56%, p-value < 0.01) (see Fig 5), 39% (95%CI; 0.23–0.54, I^2 = 98.73%, p-value < 0.01) (see Fig 6) respectively.

Figure 4: Pooled proportion of culture-positive EPTB using a random-effects model).

Figure 5: Pooled proportion of smear-positive EPTB using a random-effects model.

Figure 6: Pooled proportion of Xpert MTB/RIF assay positive EPTB using a random-effects model.

Risk of bias across studies publication

The presence of publication bias was determined using a funnel plot. By showing funnel plots with the logarithms of effect size and their standard errors, we were able to quantify publication bias. Visual inspection revealed indications of publication bias for the majority of the culture diagnostic method estimates, with most studies clustered at the funnel's apex and a few spread to the extreme right and left corners (Fig 7). The funnel plots for Xpert MTB/RIF assay and smear microscopy methods were most studies clustered at the funnel's bottom and a few spread to the extreme right corners (Fig 8 and Fig 9).

Figure 7: Funnel plot of a subgroup of 15 of the 20 selected studies

Figure 8: Funnel plot of a subgroup of 11 of the 20 selected studies

Figure 9: Funnel plot of a subgroup of 8 of the 20 selected studies

Associated risk factors of bacteriologically confirmed EPTB

The impact of each study on the overall meta-analysis summary estimate was investigated. A history of PTB infection and contact with PTB patients was found to be significant risk factors for EPTB incidence in the majority of investigations [15, 16, 19-22]. Furthermore, having regular and direct contact with live animals, as well as the consumption of raw milk, were found to be strongly related to the incidence of EPTB [16, 34]. Additionally, HIV-1 positive [19, 20, 22],

ages <14 [15], age 41-50 [20], being male [15, 20], monthly less income [16], Urban [15] were also likely to be linked to the most common EPTB remark categories.

Discussion

This systematic review and meta-analysis estimated the pooled prevalence of bacteriologically confirmed EPTB and the associated risk factors among persons suspected to have non-respiratory tuberculosis /EPTB/ in Ethiopia using published studies over the last two decades. This meta-analysis included a total of 5439 EPTB suspects from 20 studies published between 2003 and 2021. The pooled estimated prevalence of samples with any bacteriological evidence in the studies was 43% (95%CI; 0.34–0.52, I^2; 98.45%, p-value < 0.01). A previous history of PTB infection and contact with PTB patients, contact with live animals, consumption of raw milk, HIV-positive, ages <14 to 41-50, male, monthly less income, and living in an urban area with known EPTB patients were all found to be independently associated with EPTB in this study.

The overall pooled prevalence of bacteriological confirmed EPTB in this systematic review and meta-analysis data was 43%. This finding is approximately similar to that previously reported from Cameroon [35]. In contrast, when compared to the estimated prevalence of EPTB among notified TB cases in Africa, this is a high figure. According to 2017 WHO report, the prevalence of EPTB among notified TB cases in Africa and the rest of the globe was 16% and 15%, respectively [7]. Furthermore, a systematic review and meta-analysis of the prevalence of EPTB among patients living with HIV/AIDS in Sub-Saharan Africa revealed a lower prevalence of EPTB than our findings [36]. However, in the current study, the funnel plot revealed that there is publication bias, where among 20 studies included in this study, 10 were above 95% upper limit and 5 were below 95% lower limit and only 5 were within the CI. This might be due to the low number of studies conducted so far in Ethiopia and their variations in using the laboratory

methods. Thus, this may underestimate the pooled bacteriologically confirmed EPTB prevalence among EPTB presumptive cases in Ethiopia.

In this current systematic review and meta-analysis investigation, HIV-1 infected patients were the most significant risk factors associated with EPTB infection [19, 20, 22]. Similarly, several studies have examined the association between HIV-1 infection and EPTB infection [6, 37]. Furthermore, nearly half of EPTB patients were HIV-1 infected, according to a prior study [38]. This is due to the virus's immune deficiency condition, which allows the bacteria to spread from the primary infection site, the lung, to other parts of the body. During TB-HIV-1 co-infection, there is a lack of granuloma growth and functional disruption of the local immune response within the granuloma [39].

Our study showed that having a previous history of TB and a history of contact with known pulmonary TB patients was found to be significant risk factors for EPTB development [16, 19-22]. Similarly, it is well known that patients with a previous history of anti-TB treatment cases have an increased risk of EPTB [37].

In this study, we also discovered that men had a higher prevalence of EPTB involvement than women [15, 20], which is consistent with similar findings in the previous study [40]. However, another systematic review and meta-analysis study in Africa found that female patients with lymphadenitis were more common than male patients [41]. Likewise, women had a higher rate of EPTB infections than men [42]. In addition, another study reported the prevalence of EPTB was higher among females than males [43].

In the end, the current study had its limitations. Firstly, the degree of EPTB prevalence in many parts of the country has yet to be addressed, making it impossible to conclude the true burden of

EPTB in Ethiopia. Secondly, the observed publication bias that could be due to the differences in the laboratory methods might underestimate the estimated prevalence. Thirdly, Only three databases were searched. This could lead to publication bias. Finally, there is high heterogeneity among studies that might affect the true estimates. However, the findings are still significant, because the rising rate of EPTB patients in the general population is concerning.

Conclusions

The finding of this study revealed that there is a high bacteriologically confirmed of EPTB among persons suspected to have EPTB in Ethiopia. Patients having a history of previous tuberculosis, a poor income, a history of tuberculosis contact with a known PTB case, being HIV-1 positive, and having contact with PTB patients and a history of underlying diseases were all more likely to be associated with risk factors with EPTB. Thus, we recommend strengthening laboratory services for the diagnosis of EPTB in Ethiopia.

Acknowledgments

We acknowledge Ethiopian Public Health Institute for the access to article searching. We also acknowledge the authors of the original articles included in this systematic review and meta-analysis study.

Author Contributions

Conceptualization: Getu Diriba, Ayinalem Alemu.

Data curation: Getu Diriba, Habteyes Hailu Tola, Kirubel Eshetu, Ayinalem Alemu.

Formal analysis: Getu Diriba, Bazezew Yenew, Dinka Fekadu Gamtesa, Ayinalem Alemu.

Funding acquisition: Getu Diriba, Ayinalem Alemu.

Investigation: Getu Diriba, Habteyes Hailu Tola, Kirubel Eshetu, Bazezew Yenew, Ayinalem Alemu, Dinka Fekadu Gamtesa.

Validation: Kirubel Eshetu, Ayinalem Alemu.

Methodology: Getu Diriba, Ayinalem Alemu, Habteyes Hailu Tola, Kirubel Eshetu.

Software: Ayinalem Alemu, Dinka Fekadu Gamtesa.

Writing-original draft: Getu Diriba.

Writing – review & editing: Ayinalem Alemu, Habteyes Hailu Tola, Kirubel Eshetu.

Competing interests

There are no competing interests stated by the authors.

Data Availability Statement

All the important information is available within the manuscript and its supplementary files.

Funding

The author(s) received no specific funding for this work.

References

1. Prakasha SR, Suresh G, D'Sa I P, Shetty SS, Kumar SG. Mapping the pattern and trends of extrapulmonary tuberculosis. J Glob Infect Dis. 2013;5(2):54-9.
2. Adada H, Valley MA, Nour SA, Mehta J, Byrd RP, Jr., Anderson JL, *et al.* Epidemiology of extra-pulmonary tuberculosis in the United States: high rates persist in the post-HIV era. Int J Tuberc Lung Dis. 2014;18(12):1516-21.
3. World Health Organization, Global Tuberculosis Report 2018.

4. Yang D, Kong Y. The bacterial and host factors associated with extrapulmonary dissemination of Mycobacterium tuberculosis. Front Biol (Beijing). 2015;10(3):252-61.

5. Ramirez-Lapausa M M-SAA-A. Extrapulmonary tuberculosis : an overview. Rev Esp Sanid Penit 2015;17:3-11.

6. Pollett S, Banner P, O'Sullivan MV, Ralph AP. Epidemiology, Diagnosis and Management of Extra-Pulmonary Tuberculosis in a Low-Prevalence Country: A Four Year Retrospective Study in an Australian Tertiary Infectious Diseases Unit. PLoS One. 2016;11(3):e0149372.

7. World Health Organization Global tuberculosis report 2017.

8. Purohit M, Mustafa T. Laboratory Diagnosis of Extra-pulmonary Tuberculosis (EPTB) in Resource-constrained Setting: State of the Art, Challenges and the Need. J Clin Diagn Res. 2015;9(4):EE01-6.

9. Parsons LM, Somoskovi A, Gutierrez C, Lee E, Paramasivan CN, Abimiku A, *et al.* Laboratory diagnosis of tuberculosis in resource-poor countries: challenges and opportunities. Clin Microbiol Rev. 2011;24(2):314-50.

10. Lee JY. Diagnosis and treatment of extrapulmonary tuberculosis. Tuberc Respir Dis (Seoul). 2015;78(2):47-55.

11. Definitions and reporting framework for tuberculosis 2013 revision. 2013.

12. Lo CK, Mertz D, Loeb M. Newcastle-Ottawa Scale: comparing reviewers' to authors' assessments. BMC Med Res Methodol. 2014;14:45.

13. Rucker G, Schwarzer G, Carpenter JR, Schumacher M. Undue reliance on I(2) in assessing heterogeneity may mislead. BMC Med Res Methodol. 2008;8:79.

14. Huedo-Medina TB, Sanchez-Meca J, Marin-Martinez F, Botella J. Assessing heterogeneity in meta-analysis: Q statistic or I2 index? Psychol Methods. 2006;11(2):193-206.

15. Biadglegne F, Tesfaye W, Sack U, Rodloff AC. Tuberculous lymphadenitis in Northern Ethiopia: in a public health and microbiological perspectives. PLoS One. 2013;8(12):e81918.

16. Zenebe Y, Anagaw B, Tesfay W, Debebe T, Gelaw B. Smear positive extra pulmonary tuberculosis disease at University of Gondar Hospital, Northwest Ethiopia. BMC Res Notes. 2013;6:21.

17. Garedew L, Mihret A, Ameni G. Molecular typing of mycobacteria isolated from extrapulmonary tuberculosis patients at Debre Birhan Referral Hospital, central Ethiopia. Scand J Infect Dis. 2013;45(7):512-8.

18. Birhanu T BM, Ameni G. Molecular Characterization of Mycobacterium tuberculosis Complex Isolated from Tuberculous Lymphadenitis Patients at Dessie Private Hospitals, Northern Ethiopia. Journal of Biology, Agriculture and Healthcare. 2014;4(5).

19. Fanosie A, Gelaw B, Tessema B, Tesfay W, Admasu A, Yitayew G. Mycobacterium tuberculosis Complex and HIV Co-Infection among Extrapulmonary Tuberculosis Suspected Cases at the University of Gondar Hospital, Northwestern Ethiopia. PLoS One. 2016;11(3):e0150646.

20. Mulu W, Abera B, Yimer M, Hailu T, Ayele H, Abate D. Rifampicin-resistance pattern of Mycobacterium tuberculosis and associated factors among presumptive tuberculosis patients referred to Debre Markos Referral Hospital, Ethiopia: a cross-sectional study. BMC Res Notes. 2017;10(1):8.

21. Metaferia Y, Seid A, Fenta GM, Gebretsadik D. Assessment of Extrapulmonary Tuberculosis Using Gene Xpert MTB/RIF Assay and Fluorescent Microscopy and Its Risk Factors at Dessie Referral Hospital, Northeast Ethiopia. Biomed Res Int. 2018;2018:8207098.

22. Tedla E, Ayalew, G. Mycobacterium tuberculosis burden, multidrug resistance pattern, and associated risk factors among presumptive extrapulmonary tuberculosis cases at Dessie Referral Hospital, Northeast Ethiopia. The Egyptian Journal of Chest Diseases and Tuberculosis. 2020;69(3):449.

23. Korma W, Mihret A, Hussien J, Anthony R, Lakew M, Aseffa A. Clinical, molecular and drug sensitivity pattern of mycobacterial isolates from extra-pulmonary tuberculosis cases in Addis Ababa, Ethiopia. BMC Infect Dis. 2015;15:456.

24. Zewdie O, Mihret A, Ameni G, Worku A, Gemechu T, Abebe T. Molecular typing of mycobacteria isolated from tuberculous lymphadenitis cases in Addis Ababa, Ethiopia. Int J Tuberc Lung Dis. 2016;20(11):1529-34.

25. Fantahun M, Kebede A, Yenew B, Gemechu T, Mamuye Y, Tadesse M, *et al.* Diagnostic accuracy of Xpert MTB/RIF assay and non-molecular methods for the diagnosis of tuberculosis lymphadenitis. PLoS One. 2019;14(9):e0222402.

26. Assefa G, Desta K, Araya S, Girma S, Mihret A, Hailu T, *et al.* Diagnostic efficacy of Light-Emitting Diode (LED) Fluorescence based Microscope for the diagnosis of Tuberculous lymphadenitis. PLoS One. 2021;16(7):e0255146.

27. Abdissa K, Tadesse M, Bezabih M, Bekele A, Apers L, Rigouts L, *et al.* Bacteriological methods as add on tests to fine-needle aspiration cytology in diagnosis of tuberculous lymphadenitis: can they reduce the diagnostic dilemma? BMC Infect Dis. 2014;14:720.

28. Tadesse M, Abebe G, Abdissa K, Aragaw D, Abdella K, Bekele A, *et al.* GeneXpert MTB/RIF Assay for the Diagnosis of Tuberculous Lymphadenitis on Concentrated Fine Needle Aspirates in High Tuberculosis Burden Settings. PLoS One. 2015;10(9):e0137471.

29. Abdissa K, Tadesse M, Abdella K, Bekele A, Bezabih M, Abebe G. Diagnostic performance of fluorescent light-emitting diode microscopy for tuberculous lymphadenitis in a high-burden setting. Trop Med Int Health. 2015;20(11):1543-8.

30. Tadesse M, Abebe G, Bekele A, Bezabih M, Yilma D, Apers L, *et al.* Xpert MTB/RIF assay for the diagnosis of extrapulmonary tuberculosis: a diagnostic evaluation study. Clin Microbiol Infect. 2018;25(8):1000-5.

31. Yassin MA, Olobo JO, Kidane D, Negesse Y, Shimeles E, Tadesse A, *et al.* Diagnosis of tuberculous lymphadenitis in Butajira, rural Ethiopia. Scand J Infect Dis. 2003;35(4):240-3.

32. Iwnetu R, van den Hombergh J, Woldeamanuel Y, Asfaw M, Gebrekirstos C, Negussie Y, *et al.* Is tuberculous lymphadenitis over-diagnosed in Ethiopia? Comparative performance of diagnostic tests for mycobacterial lymphadenitis in a high-burden country. Scand J Infect Dis. 2009;41(6-7):462-8.

33. Derese Y HE, Assefa T, Bekele Y, Mihret A, Aseffa A. Comparison of PCR with standard culture of fine needle aspiration samples in the diagnosis of tuberculosis lymphadenitis. J Infect Dev Ctries 2012;6(1):53-7.

34. Berg S, Schelling E, Hailu E, Firdessa R, Gumi B, Erenso G, *et al.* Investigation of the high rates of extrapulmonary tuberculosis in Ethiopia reveals no single driving factor and

minimal evidence for zoonotic transmission of Mycobacterium bovis infection. BMC Infect Dis. 2015;15:112.

35. Namme L, Achu J, Christopher K, Elvis T, Hugo Bertrand M, Marie-Solange D. Extrapulmonary tuberculosis and HIV coinfection in patients treated for tuberculosis at the Douala General Hospital in Cameroon. Annals of Tropical Medicine and Public Health. 2013;6(1):100.

36. Mohammed H, Assefa N, Mengistie B. Prevalence of extrapulmonary tuberculosis among people living with HIV/AIDS in sub-Saharan Africa: a systemic review and meta-analysis. HIV AIDS (Auckl). 2018;10:225-37.

37. Qian X, Nguyen DT, Lyu J, Albers AE, Bi X, Graviss EA. Risk factors for extrapulmonary dissemination of tuberculosis and associated mortality during treatment for extrapulmonary tuberculosis. Emerg Microbes Infect. 2018;7(1):102.

38. Arega B, Mersha A, Minda A, Getachew Y, Sitotaw A, Gebeyehu T, *et al.* Epidemiology and the diagnostic challenge of extra-pulmonary tuberculosis in a teaching hospital in Ethiopia. PLoS One. 2020;15(12):e0243945.

39. Diedrich CR, Flynn JL. HIV-1/mycobacterium tuberculosis coinfection immunology: how does HIV-1 exacerbate tuberculosis? Infect Immun. 2011;79(4):1407-17.

40. Yone EW, Kengne AP, Moifo B, Kuaban C. Prevalence and determinants of extrapulmonary involvement in patients with pulmonary tuberculosis in a Sub-Saharan African country: a cross-sectional study. Scand J Infect Dis. 2013;45(2):104-11.

41. Mekonnen D, Derbie A, Abeje A, Shumet A, Nibret E, Biadglegne F, *et al.* Epidemiology of tuberculous lymphadenitis in Africa: A systematic review and meta-analysis. PLoS One. 2019;14(4):e0215647.

42. Lin CY, Chen TC, Lu PL, Lai CC, Yang YH, Lin WR, *et al*. Effects of gender and age on development of concurrent extrapulmonary tuberculosis in patients with pulmonary tuberculosis: a population based study. PLoS One. 2013;8(5):e63936.

43. R E, M N. Epidemiological profile among pulmonary and extrapulmonary tuberculosis patients in Laayoune, Morocco. Pan African Medical Journal. 2020;37(56).

Supporting documents

S1: File: PRISMA checklist

S2: File: Literature search strategy from searched databases

S3: File: Detailed data of the included studies

S4: File: Newcastle-Ottawa quality assessment scale for cross-sectional studies

Figure Click here to access/download;Figure;Fig 1.pdf

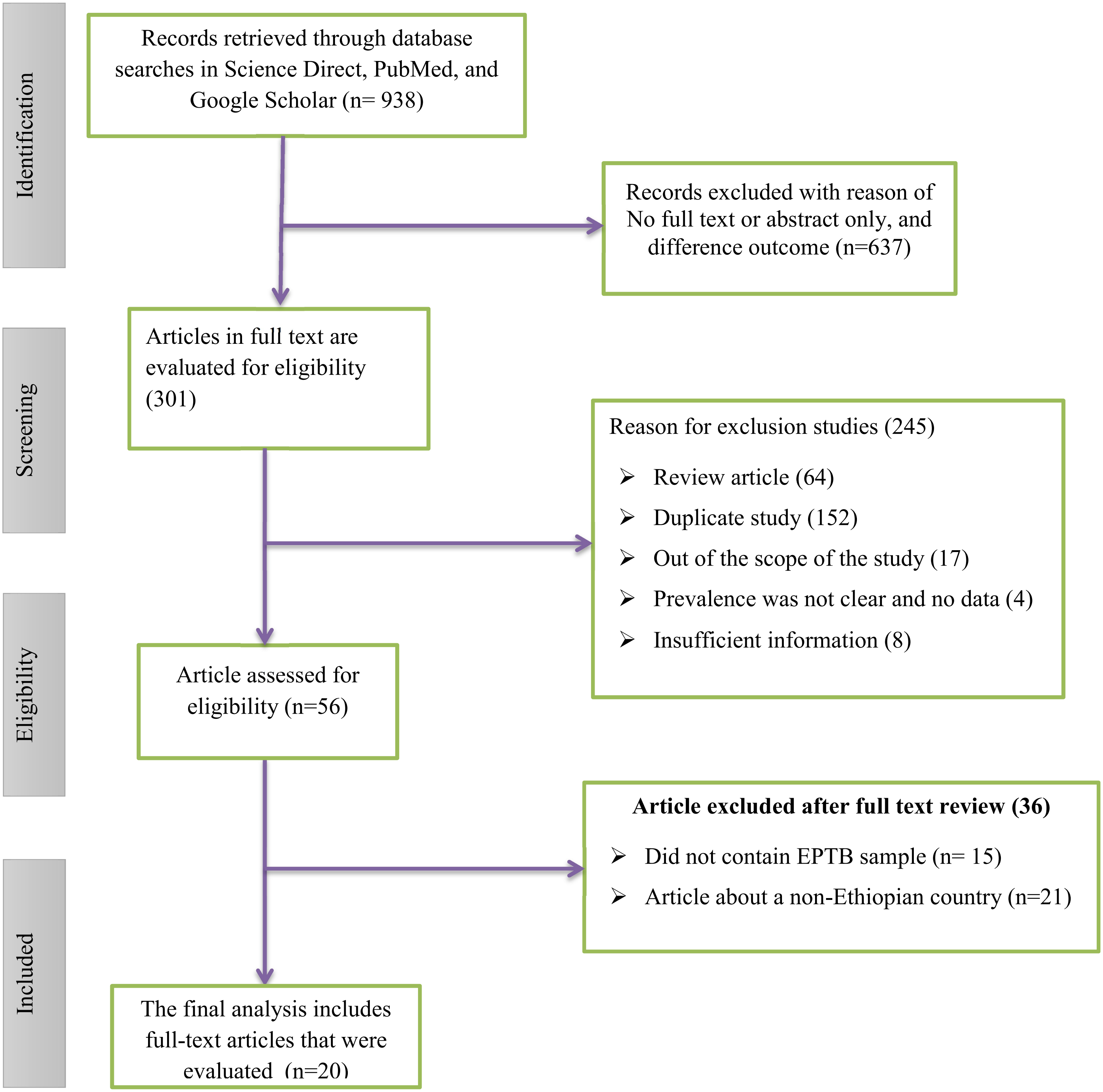

Figure

Click here to access/download;Figure;Fig 2.pdf

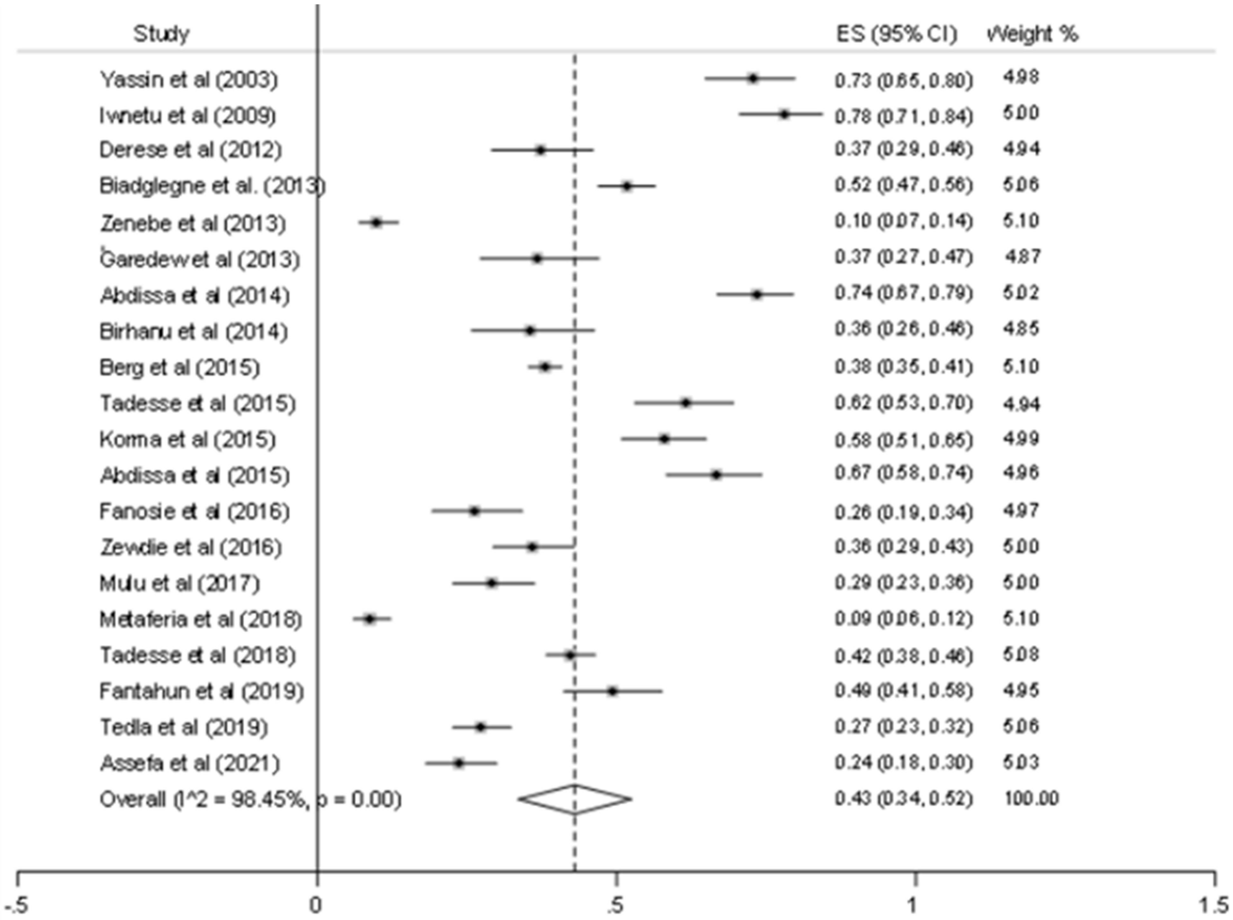

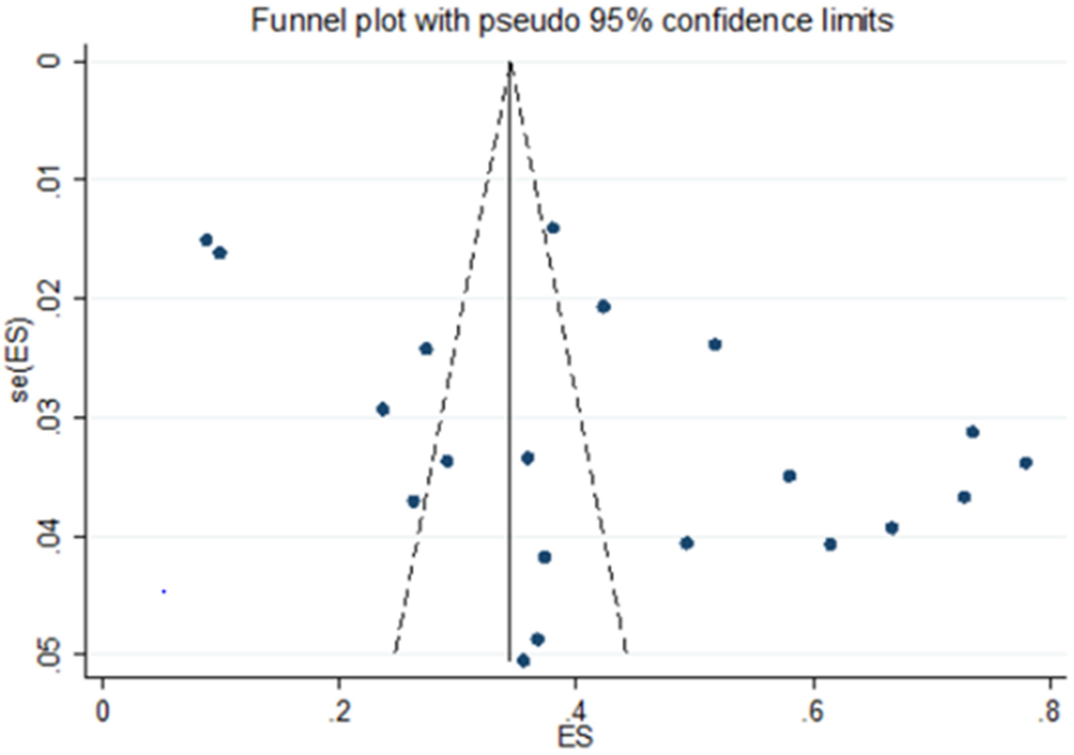
Funnel plot with pseudo 95% confidence limits
se(ES)
0
.01
.02
.03
.04
.05
0
.2
.4
.6
.8
ES

Figure
Click here to access/download;Figure;Fig 4.pdf

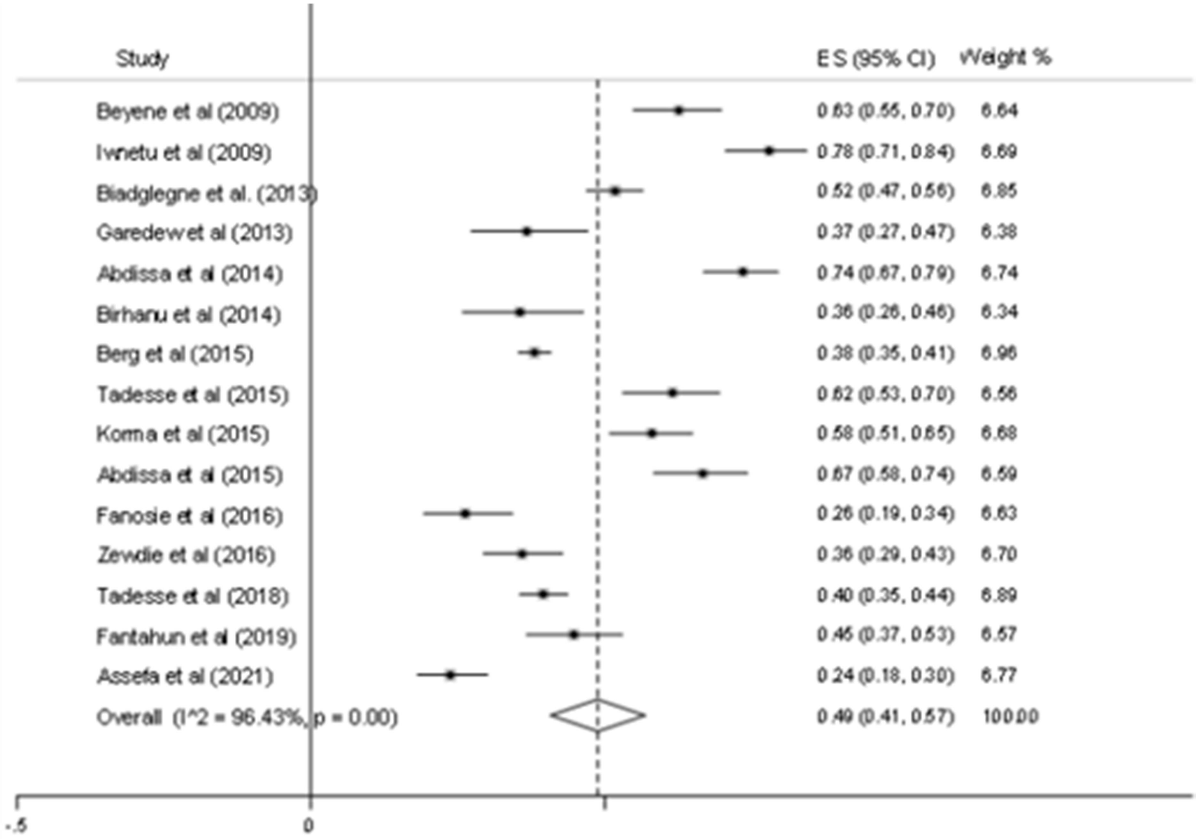

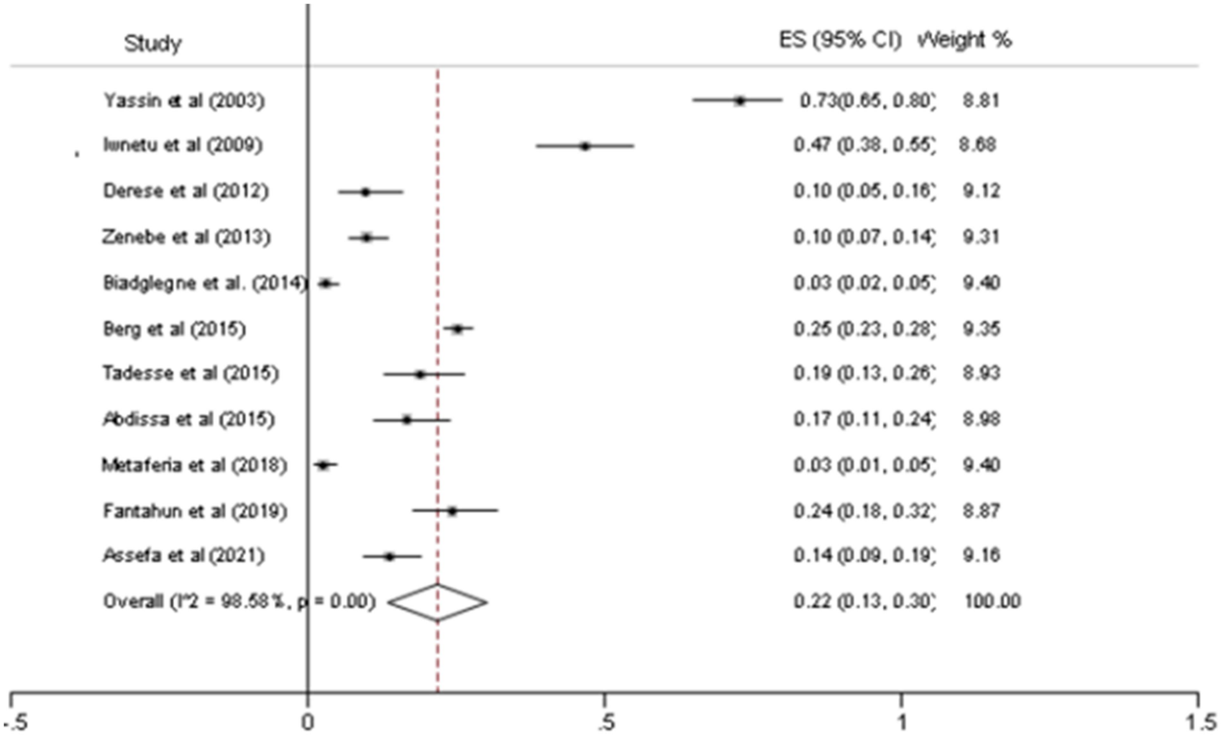
Study
ES (95% CI) Weight %
Yassin et al (2003) 0.73(0.65, 0.80) 8.81
Iwnetu et al (2009) 0.47 (0.38, 0.55) 8.68
Derese et al (2012) 0.10 (0.05, 0.16) 9.12
Zenebe et al (2013) 0.10 (0.07, 0.14) 9.31
Biadglegne et al. (2014) 0.03 (0.02, 0.05) 9.40
Berg et al (2015) 0.25 (0.23, 0.28) 9.35
Tadesse et al (2015) 0.19 (0.13, 0.26) 8.93
Abdissa et al (2015) 0.17 (0.11, 0.24) 8.98
Metaferia et al (2018) 0.03 (0.01, 0.05) 9.40
Fantahun et al (2019) 0.24 (0.18, 0.32) 8.87
Assefa et al (2021) 0.14 (0.09, 0.19) 9.16
Overall (I^2 = 98.58%, p = 0.00) 0.22 (0.13, 0.30) 100.00
-.5
0
.5
1
1.5

Figure

Click here to access/download;Figure;Fig 6.pdf

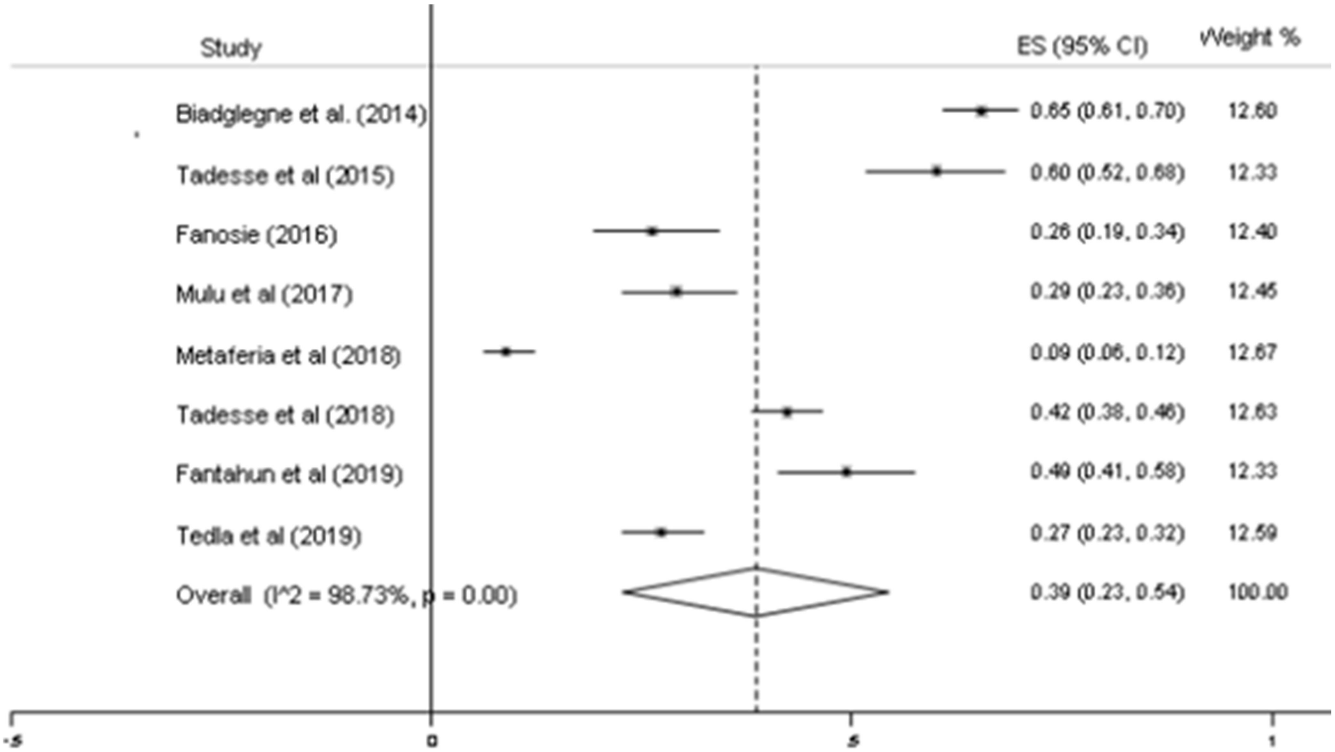

Figure Click here to access/download;Figure;Fig 7.pdf

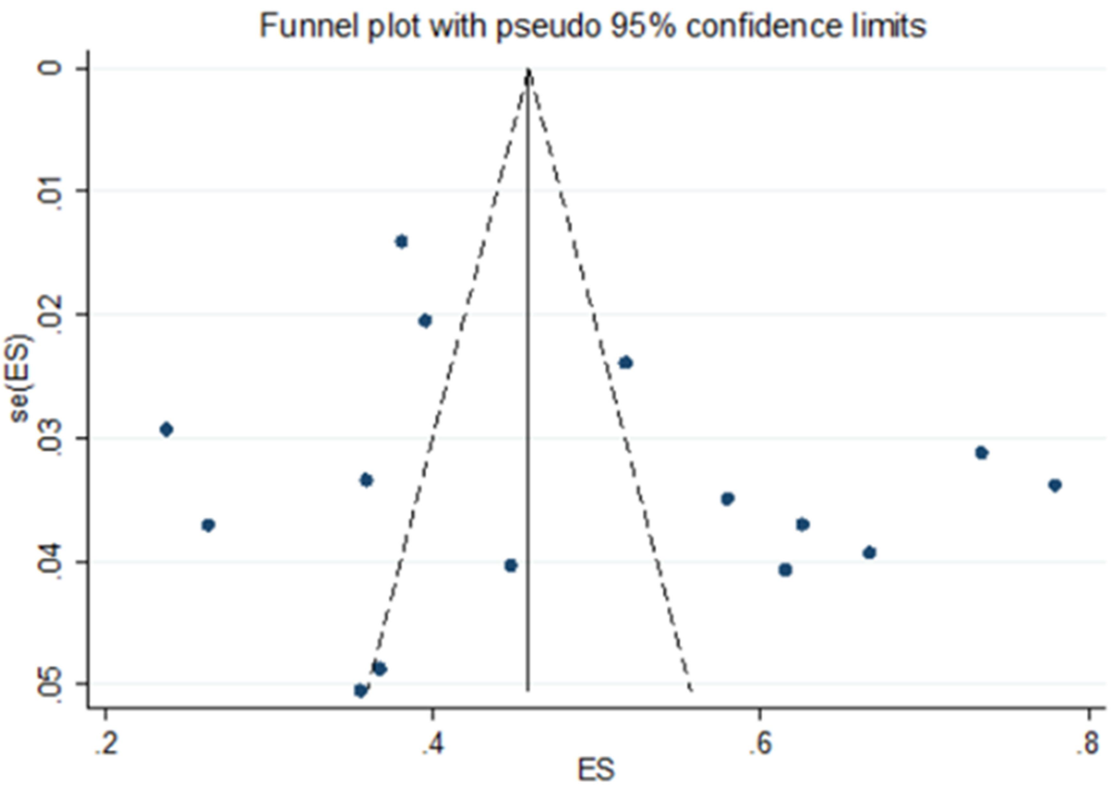

Click here to access/download;Figure;Fig 8.pdf

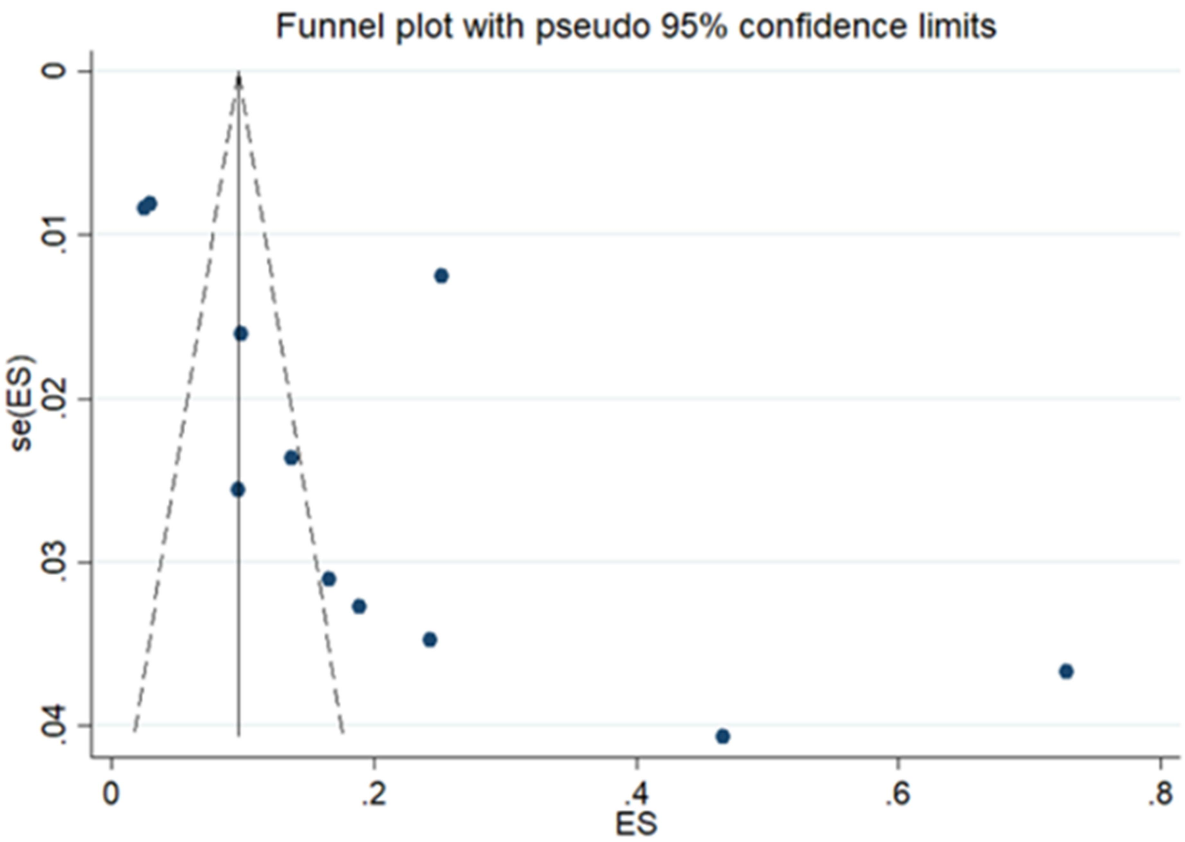

Click here to access/download;Figure;Fig 9.pdf

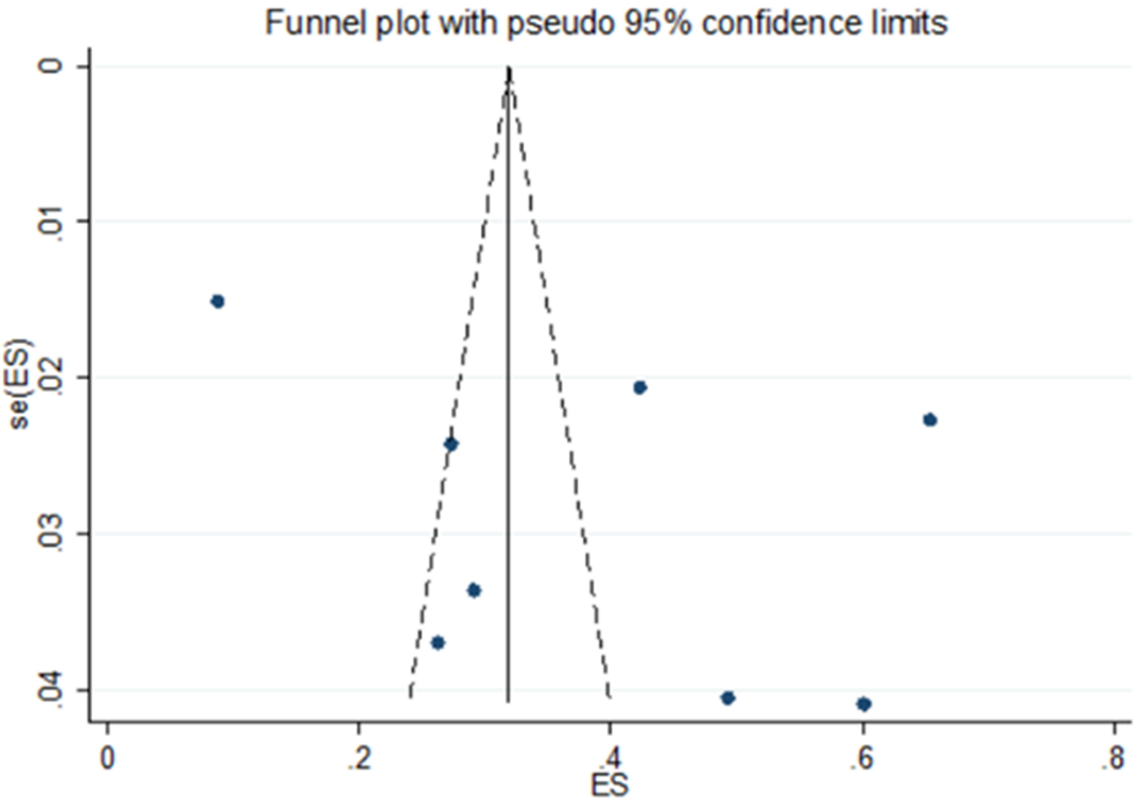

Figure

Click here to access/download;Figure;Figure 1.pdf

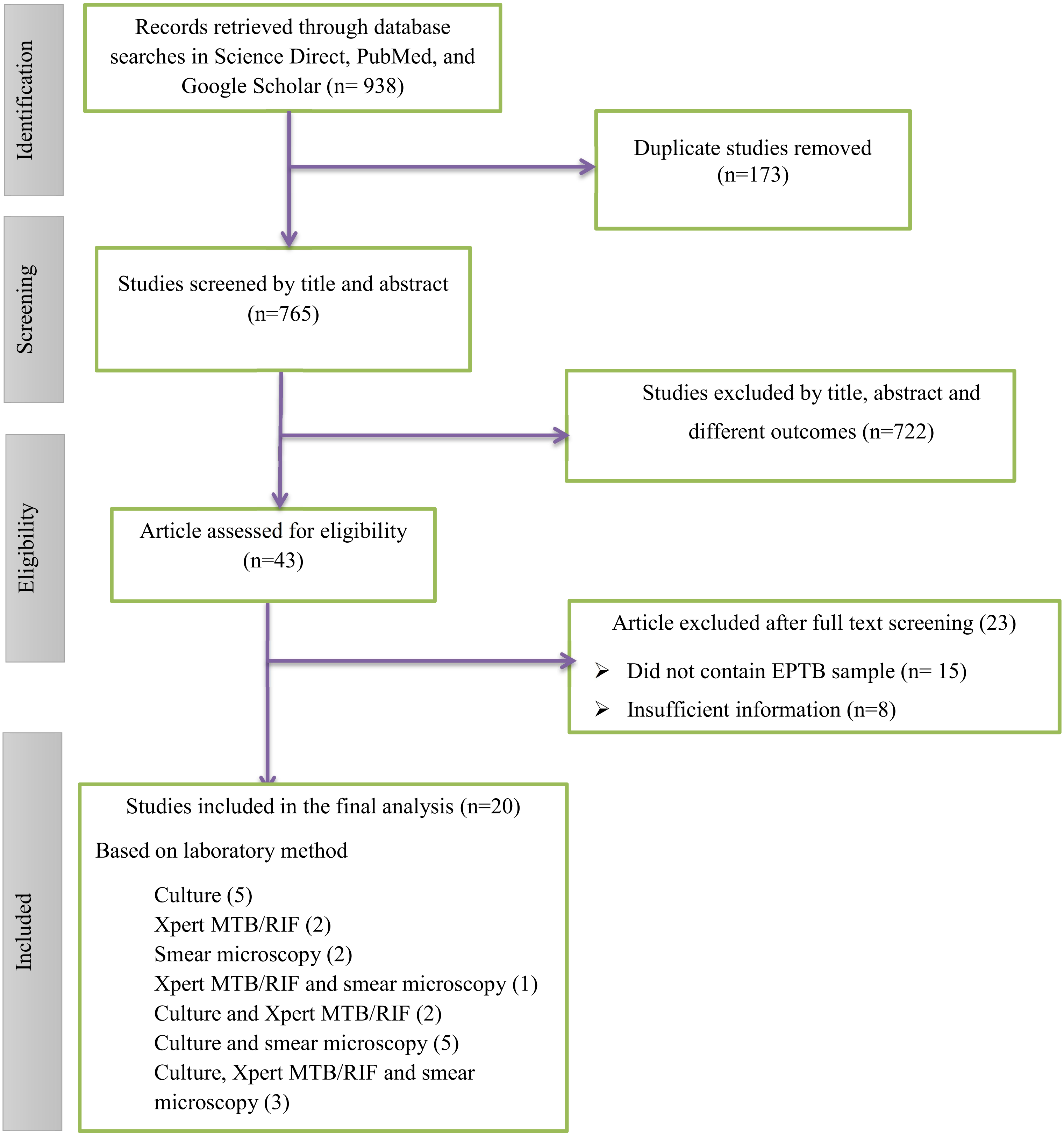

Figure

Click here to access/download;Figure;Figure 2.pdf

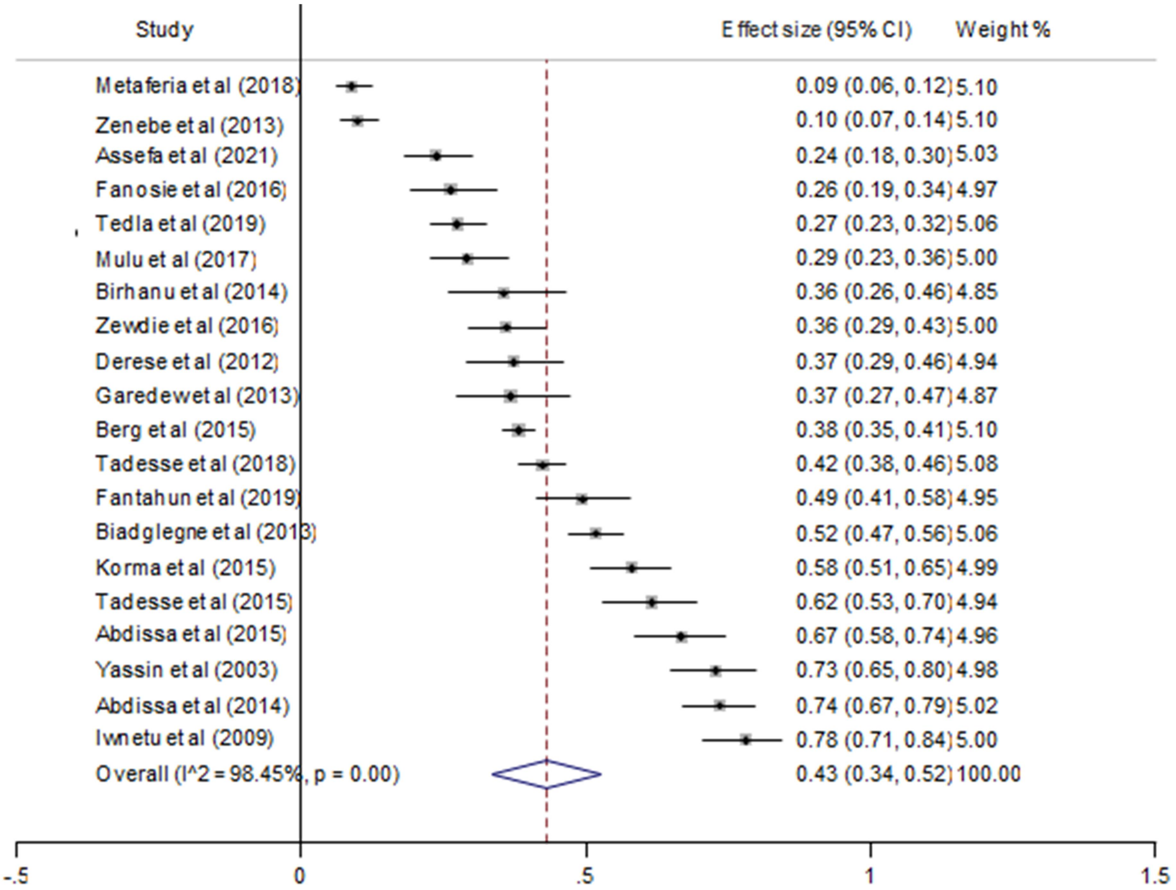

Figure Click here to access/download;Figure;Figure 3.pdf

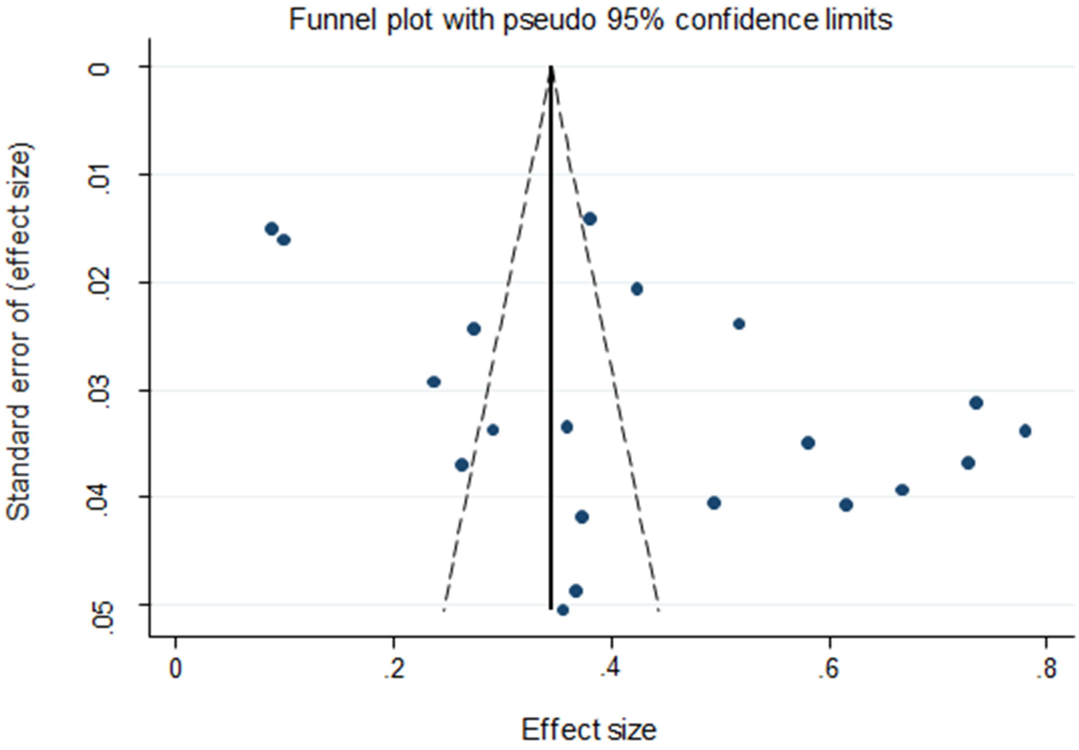

Figure
Click here to access/download;Figure;Figure 4.pdf

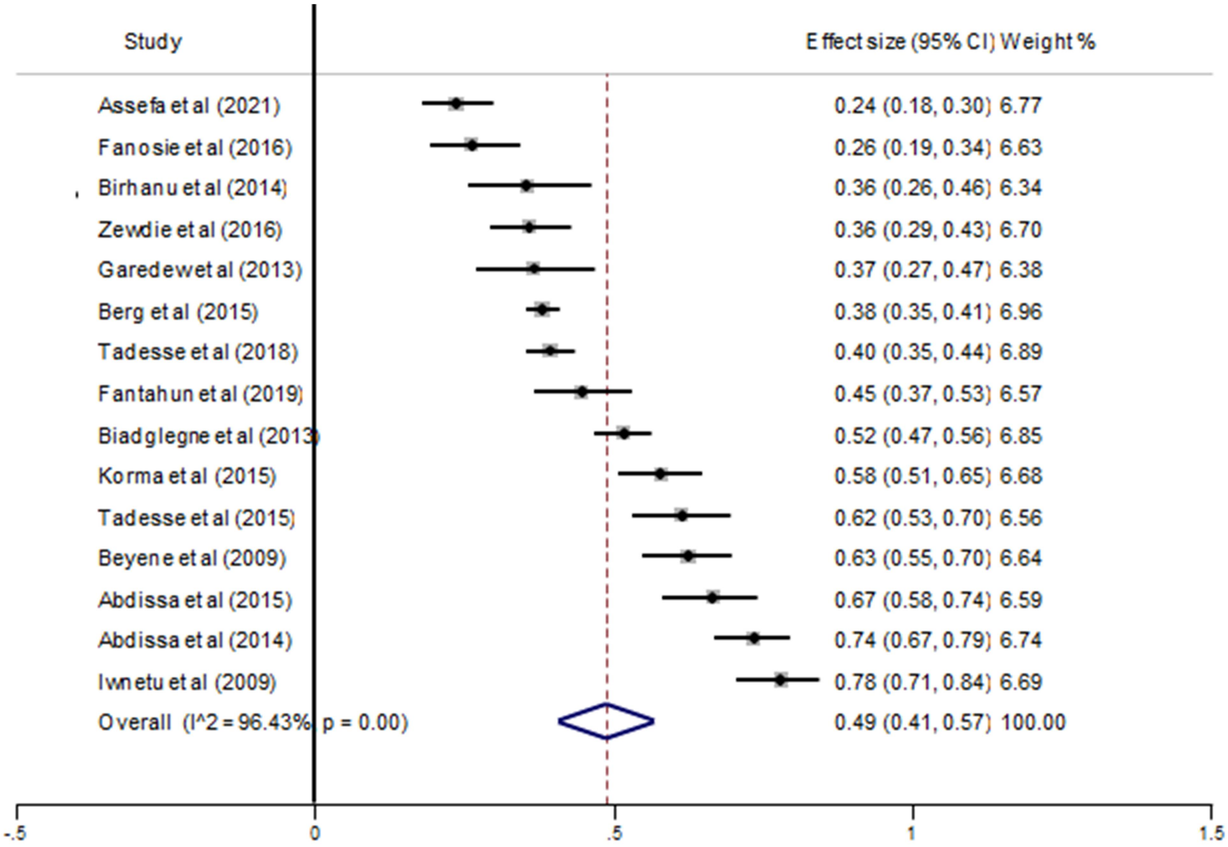

Figure
Click here to access/download;Figure;Figure 5.pdf

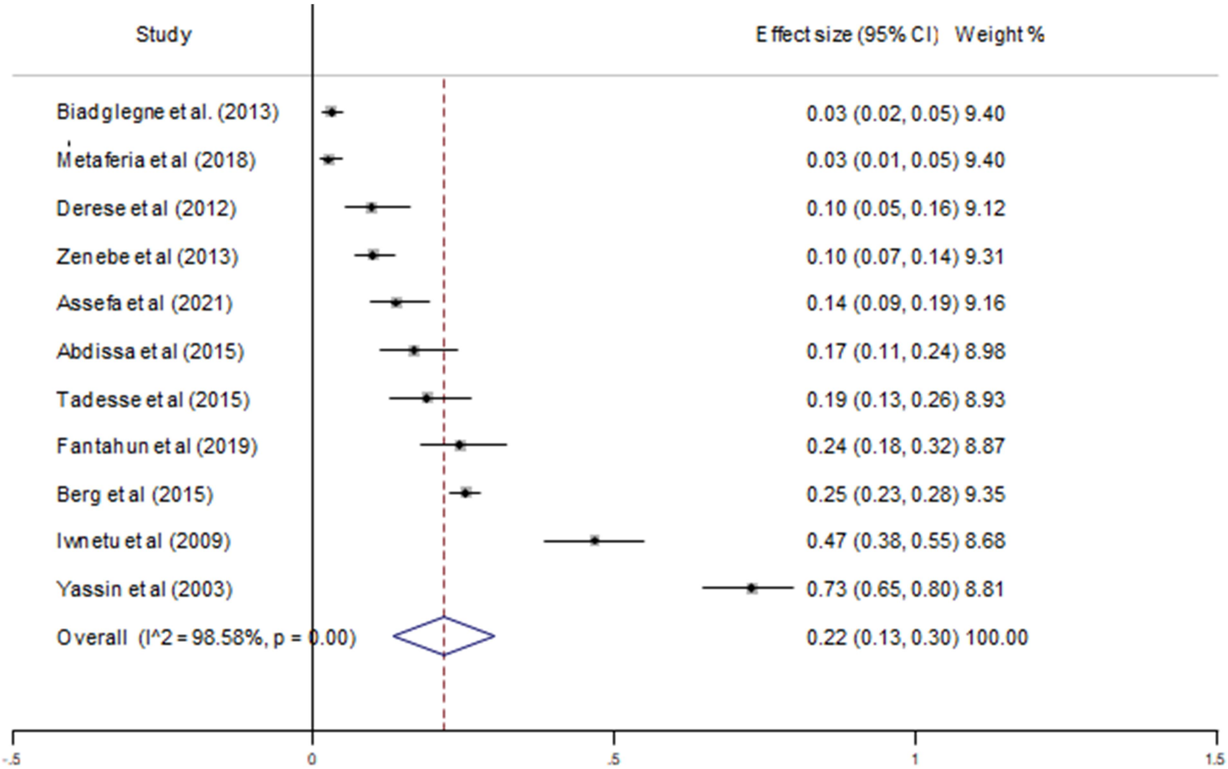

Figure
Click here to access/download;Figure;Figure 6.pdf

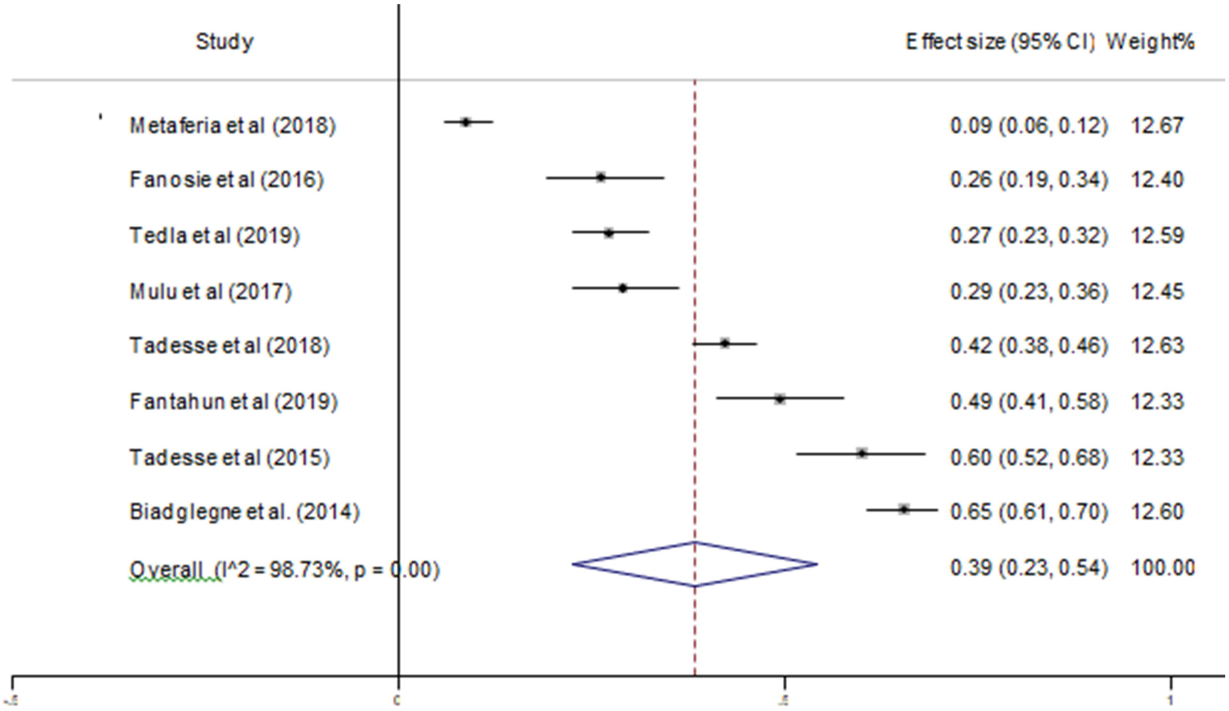

Click here to access/download;Figure;Figure 7.pdf

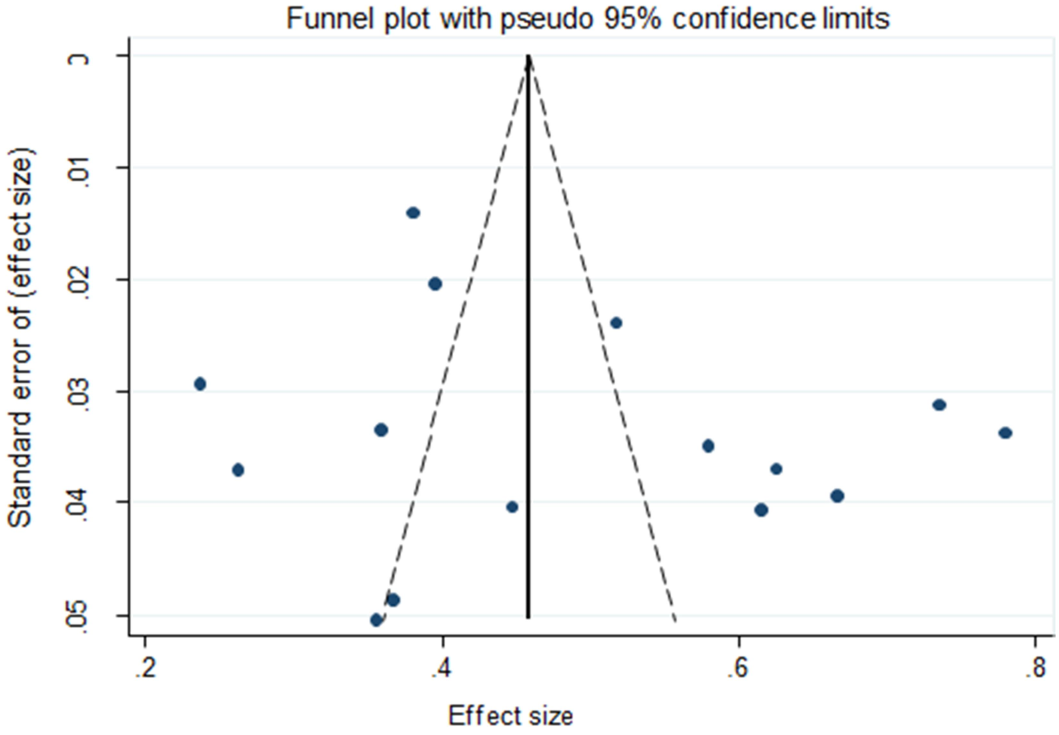

 Click here to access/download;Figure;Figure 8.pdf

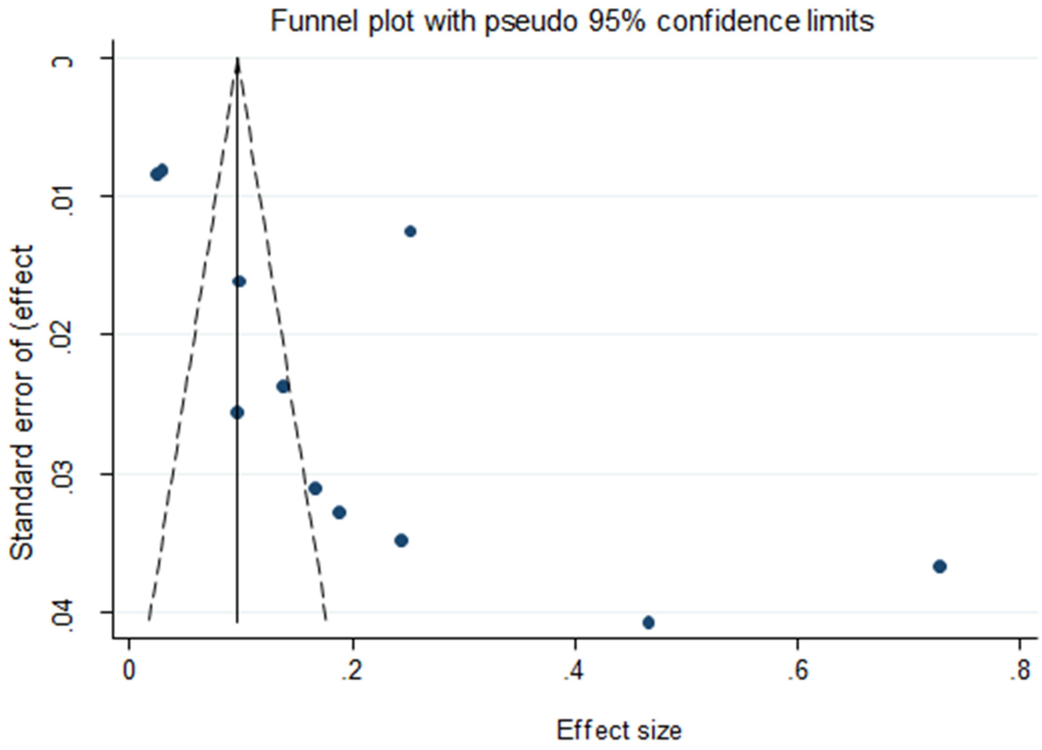

 Click here to access/download;Figure;Figure 9.pdf

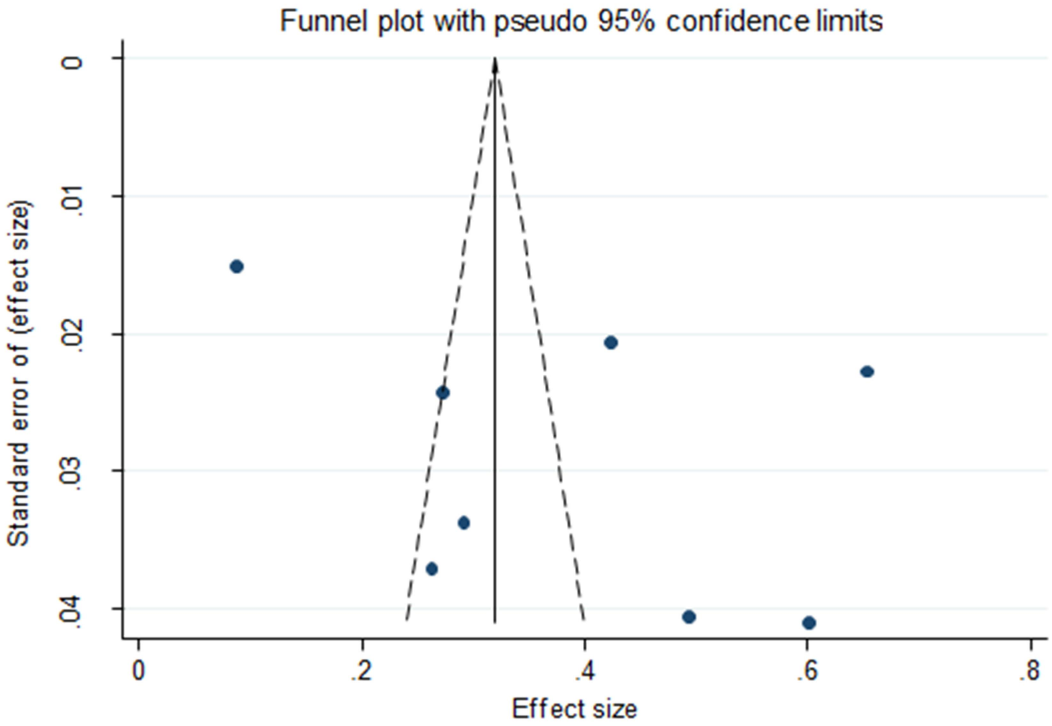

Click here to access/download
Supporting Information
S2 File.docx

Click here to access/download
Supporting Information
S3 File.xlsx

Click here to access/download
Supporting Information
S4 File.docx

Click here to access/download
Supporting Information
S1 File.doc

Bacteriologically confirmed extrapulmonary tuberculosis and the associated risk factors among extrapulmonary tuberculosis suspected patients in Ethiopia: A systematic review and meta-analysis

Getu Diriba[1*], Ayinalem Alemu[1,2], Kirubel Eshetu[3], Bazezew Yenew[1], Dinka Fikadu Gamtesa[1], Habteyes Hailu Tola[1]

[1]Ethiopian Public Health Institute, Addis Ababa, Ethiopia

[2]Aklilu Lemma Institute of Pathobiology, Addis Ababa University

[3]USAID Eliminate TB Project, Management Sciences for Health, Addis Ababa, Ethiopia

[*]Corresponding author Getu Diriba

Ethiopian Public Health Institute

Phone: +251913828019. Fax: +2510112780431

P.O.Box 1242, Addis Ababa, Ethiopia

Email: getud2020@gmail.com

Abstract

Background: The actual burden of bacteriologically confirmed extrapulmonary tuberculosis (EPTB) and risk factors in Ethiopia is not well known due to lack of a strong surveillance system in Ethiopia. Thus, this is study was conducted to estimate the pooled prevalence of bacteriologically confirmed EPTB and the associated risk factors among persons suspected to have non-respiratory tuberculosis /EPTB/ assess the prevalence of bacteriologically confirmed EPTB and risk factors in Ethiopia.

Methods: A systematic review and meta-analysis of published studies reporting the prevalence of EPTB from searched electronic databases; Science Direct, PubMed, and Google Scholar estimated spread across the research periods, nationally, and in different areas, using a random-effects model. We used I^2 to analyze heterogeneity in reported prevalence of bacteriologically confirmed extrapulmonary tuberculosis.

Results: After reviewing 938 research articles, 20 studies (19 cross-sectional and 1 retrospective) from 2003 to 2021 were included in the final analyses. In the majority of research, we found variation in study design and study population characteristics, as well as a medium/high risk of bias. The pooled prevalence of bacteriologically confirmed EPTB was 43% (95%CI; 0.34–0.52, I^2 = 98.45%). The asymmetry of the funnel plot revealed the presence of publication bias. Specifically the pooled prevalence of bacteriologically confirmed EPTB based on smear microscopy, Xpert MTB/RIF assay, and culture were 22% (95%CI; 0.13–0.30, I^2 = 98.56%), 39% (95%CI; 0.23–0.54, I^2 = 98.73%) and 49% (95%CI; 0.41–0.57, I^2 = 96.43%) respectively. In this study, having a history of previous pulmonary tuberculosis (PTB)PTB

Formatted: Not Highlight

Formatted: Not Highlight

Formatted: Not Highlight

contact with PTB patients, contact with live animals, consumption of raw milk, HIV-positive, ~~being~~ male, and lower monthly income, were found to be independently associated with EPTB.

Formatted: Font: 11 pt, Highlight

Conclusion: Ethiopia has a high rate of bacteriologically confirmed EPTB. A history of previous PTB, being HIV-positive, and having contact with PTB patients were all associated with EPTB in the majority of studies. Strengthening laboratory services for EPTB diagnosis should be given priority in order to diagnose EPTB cases as early as possible.

Keywords: Prevalence, Pulmonary Tuberculosis, Extrapulmonary Tuberculosis, Risk factors

~~1.~~ Introduction

Formatted: Font: (Default) Times New Roman, 12 pt, Bold, Kern at 16 pt

Formatted: Normal, No bullets or numbering

Pulmonary tuberculosis (PTB), which affects the lungs, accounts for 85% of all reported tuberculosis cases globally [~~(~~1~~)~~]. However, extrapulmonary tuberculosis (EPTB), which affects parts of the body other than the lung is becoming a major concern in TB prevention and control efforts [~~(~~2~~)~~]. The prevalence of EPTB among notified TB cases in the African region in the 2018 global report was 16%, which is the ~~second~~ second-highest next to the Eastern Mediterranean region (24%), which is more than the global prevalence of EPTB (15%). The lowest prevalence was reported ~~from~~ in the Western Pacific region (8%) [~~(~~3~~)~~]. EPTB ~~Extrapulmonary Tuberculosis~~ is assumed to be produced by the spread of bacteria through the bloodstream from a primary focus in the lung, and hence represents a disseminated form of tuberculosis. EPTB most commonly affects the lymph nodes, abdomen, pleura, bones, and meninges, but the prevalence varies with age, sex, and geographic location [~~(~~4~~)~~].

Extrapulmonary TB remains a critical concern both in developing and developed countries. EPTB accounts for 15% to 30% of all tuberculosis cases [~~(~~5, 6~~)~~]. In persons with HIV/AIDS and

other immunocompromised states, it is a prevalent opportunistic infection [(7)]. In Ethiopia, there is a scarcity of evidence on bacteriological diagnosis and evaluation of EPTB.

Extrapulmonary tuberculosis is difficult to diagnose for a variety of reasons. Many types of EPTB necessitate invasive diagnostic sampling, which can be dangerous to the patient and expensive. Because most types of EPTB are paucibacillary (TB disease caused by a limited number of bacteria), – and detection by smear microscopy is less sensitive. This particularly affects resource -–limited settings, where the more sensitive methods of mycobacterial culture examination are not widely available. Culture has its own set of drawbacks such as very long Turnaround turnaround Ttime and– necessitates a well-equipped biosafety laboratory [(8)]. Molecular methods are a quick and sensitive procedure that only requires a small amount of sample and may be used on killed bacteria; however, they require highly trained technologists and can be expensive [(9)]. As a result of these challenges, EPTB is frequently diagnosed solely based on clinical suspicion, and many people are given the incorrect diagnosis, resulting in needless TB treatment or poor outcomes from untreated EPTB. Even in tertiary health care facilities, the majority of patients have beenhad started anti-tuberculosis therapy TB medicine without bacteriological evidence. These people were misdiagnosed or received therapy too late, and they overestimated the scale of the problem at the community level [(10)]. There is a scarcity of data on the bacteriologically confirmedprevalence of EPTB among suspected EPTB individuals in Ethiopia. There isn't a comprehensive review and meta-analysis of the current research to uncover evidence to support policymaking. As a result, this study aimed to we investigate the prevalence of bacteriologically confirmed EPTB and the associated risk factors amongst persons with suspected to have non-respiratory TB /EPTB/ assessed the prevalence bacteriologically confirmed extrapulmonary tuberculosis and risk factors in Ethiopia.

~~2.~~ Materials and methods

Formatted: Normal, No bullets or numbering

~~2.1.~~ Search strategy

Formatted: Indent: Left: 0.25", No bullets or numbering

We systematically searched electronic databases such as MEDLINE (PubMed), Science Direct, and gray literature sources such as Google Scholar and Google for articles published in English language. We used the key terms such as “Tuberculosis lymph node”, “Tuberculosis cardiovascular”, “Tuberculosis central nervous system”, “Tuberculosis cutaneous”, “Tuberculosis endocrine”, “Tuberculosis gastrointestinal”, “Tuberculosis hepatic”, “Tuberculosis ocular”, “Tuberculosis osteoarticular”, “Tuberculosis pleura”, “Tuberculosis splenic”, “Tuberculosis urogenital”, and “Ethiopia” both in MeSH and free text.

~~2.2.~~ Eligibility criteria

Formatted: Indent: Left: 0.25", No bullets or numbering

We included ~~the~~ studies that reported the prevalence of EPTB in Ethiopia. Observational studies, such as cohort (prospective or retrospective) and cross-sectional studies, were included. Studies that were written in English ~~language~~ and published before October 26, 2021 (last date of the searching date), were considered. The studies were included regardless of the diagnostic methods used. The articles without a journal name and/or authors, conference proceedings or presentations, and reviews were excluded from the final analysis.

~~2.3.~~ Data extraction

Formatted: Indent: Left: 0.25", No bullets or numbering

We created a data extraction sheet using a Microsoft Excel® 2010~~Microsoft Excel~~ worksheet. Two independent authors (GD, AA) extracted data including study period, study setting (community or facility-based), study site, test method, sample size, and the number of positive

patients. The third author (DF) resolved the inconsistencies that arose between the two authors. To ensure consistency, another co-author (HHT) independently examined the extracted data.

~~2.4.~~ Operational Definition

Formatted: Indent: Left: 0.25", No bullets or numbering

In this meta-analysis, ~~the following definitions related to extrapulmonary tuberculosis were used; according to WHO, bacteriological confirmation extrapulmonary tuberculosis was defined as confirmed if any diagnostic test result was positive (smear microscopy, culture, Xpert MTB/RIF and/or nucleic acid amplification tests)~~the WHO definition of a positive test result was applied. This states that a positive diagnostic test result using smear microscopy, culture, Xpert MTM/RIF or nucleic acid amplification test is a bacteriological confirmation of an EPTB infection [~~(~~11~~)~~].

~~2.5.~~ Risk of bias assessment and study quality

Formatted: Indent: Left: 0.25", No bullets or numbering

Two authors (GD and DF) independently assessed the quality of the ~~included~~ studies using the Newcastle-Ottawa quality assessment scale adapted for cross-sectional studies [~~(~~12~~)~~]. ~~A consensus was reached between the two authors to end their issue. A third author (AA) was consulted in the event of a persistent disagreement.~~ The tool has three components: selection, comparability, and outcome/exposure. The selection part is scored from zero to five stars, and the comparability is scored from zero to two stars. The outcome is scored from zero to three stars. To minimise the subjective interpretation of bias from scoring two reviewers (GD and DF) were assessed the quality of individual studies. Furthermore, when the disagreements that occurred throughout the quality grading process were settled by consulting a third author (AA) ~~Studies were evaluated as having a high, moderate or low risk of bias using customized checklists of the Newcastle-Ottawa quality assessment scale~~ [~~(~~12~~)~~]. We used I^2 to analyze

Field Code Changed

Formatted: Highlight

heterogeneity in reported prevalence [~~(~~13~~)~~]. The presence of heterogeneity was defined as an $I^2 \geq$ 50% [~~(~~14~~)~~]. A funnel plot was also used to investigate the presence of publication bias.

~~2.6.~~ Statistical analysis

Formatted: Indent: Left: 0.25", No bullets or numbering

For statistical analysis, STATA® 14.2 StataCorp LLC, Texas, USA~~STATA~~ software was employed. We estimated the pooled prevalence of EPTB and ~~its~~ a 95% confidence interval using a random-effect meta-analysis model. The 'metaprop' command ~~on~~ in STATA 14 (STATA Corporation, College Station, TX, USA) was used to determine the pooled prevalence of EPTB in patients with suspected TB. The distributional information~~prevalence~~ of EPTB was displayed using a forest plot.

Ethical consideration and consent

Since this study is based on previously published articles ethical approval is not applicable.

Result

Study selection

Formatted: Font: (Default) Times New Roman, 12 pt, Bold

Formatted: Normal, Indent: Left: 0"

The three electronic databases yielded a total of 938 articles, which were then imported into an Endnote X8 citation manager~~.~~, and 173 duplicates were removed. Next, 722 articles were screened by title, abstract and difference outcome. Around 43 articles that passed the first stage were assessed through a full-text review. During this review, the study subjects, study design, study quality, and outcome were considered. Due to this reasen 23 articles removed. Finally, 20 articles became eligible for data extraction. ~~Due to differences in outcome, conference abstracts and studies were excluded at this stage. Due to duplication and out of the study's scope, review type of articles, and lack of clear prevalence data, additional 245 publications were removed. . Then, due to the article on pulmonary tuberculosis and an article about a non-Ethiopian country,~~

36 further types of research were excluded. Finally, 20 papers met all the inclusion criteria. Regarding the EPTB laboratory diagnostic method used in the selected 20 studies, five studies used culture methods, five studies used culture and smear microscopy and three studies used culture, Xpert MTB/RIF and smear microscopy, two studies used Xpert MTB/RIF, two studies used smear microscopy, two studies used culture and Xpert MTB/RIF, one study used Xpert MTB/RIF and Smear microscopy. The methodical selection of literature is depicted in Fig 1. The recommended reporting items for systematic reviews and meta-analysis (PRISMA) flow diagram were used to complete the overall screening (Fig 1).

Figure 1: shows the PRISMA flow diagram, which depicts the study screening and selection process.

2.7. Characteristics of the studies included in the review

Formatted: Indent: Left: 0.25", No bullets or numbering

Of the total of 20 articles reviewed, eight studies were undertaken in the Amhara region [(15-22)], four studies in Addis Ababa [(23-26)], four in Oromia [(27-30)], one study in the Southern Nations Nationalities and People Region [(31)], and the remaining three studies were based on data collected from- different regions of the country [(32-34)]. The data collection period ranges from 1998 [(31)] to 2020 [(26)]. The sample size investigated was ranged from 90 [(18)] to 1,198 participants [(34)]. The majority of the studies were cross-sectional studies, with one retrospective study. Fourteen studies only examined one type of sample, while the remaining six studies reported on evaluating various sample types. Regarding diagnostic methods, five studies used smear microscopy and culture methods [(26, 29, 32-34)], three studies used smear microscopy, culture, and Xpert MTB/RIF assay [(15, 25, 28)], two studies applied smear microscopy method [(16, 31)], two studies used the Xpert MTB/RIF assay [(20, 22)], two studies

used the culture and Xpert MTB/RIF assay [~~(~~19, 30~~)~~], ~~another~~ one study used the Xpert MTB/RIF and microscopy methods [~~(~~21~~)~~], and the remaining five studies used the culture method [~~(~~17, 18, 23, 24, 27~~)~~] (Table 1).

Table 1: The general characteristics of the studies that ~~was~~ were included in the review.

First Author [ref.]	Publication Year	Study Design	Study area	Study Setting	Study time	Sample size	Positive	Type of EPTB	Diagnostic Method	Associated factors
Yassin et al [~~(~~31~~)~~]	2003	cross-sectional	Butajira	Facility-based	1998-2000	147	107	Lymph node	Microscopy	
Iwnetu et al [~~(~~32~~)~~]	2009	cross-sectional	Addis Ababa, Bar Dar, Diredawa	Facility-based	2004-2005	150	117	Lymph node	Microscopy Culture	
Derese et al [~~(~~33~~)~~]	2012	retrospective	Woldia, Butajira, Gonder	Facility-based	2011	134	50	Lymph node	Microscopy Culture	
Biadglegne et al [~~(~~15~~)~~]	2013	cross~~-~~sectional	Bahirdar, Gondar & Dessie	Facility-based	2012	437	226	Lymph node	Microscopy Culture Xpert MTB/RIF	Retreated, M[illegible] Age < 14, Urban
Zenebe et al [~~(~~16~~)~~]	2013	cross-sectional	Gonder	Facility-based	2012	344	34	Lymph node	Microscopy	previous histo[illegible] PTB, raw milk, monthly less income, TB contact
Garedew et al [~~(~~17~~)~~]	2013	cross-sectional	Debre Birhan	Facility-based	2010-2011	98	36	Lymph node	Culture	
Abdissa et el [~~(~~27~~)~~]	2014	cross-sectional	Jimma	Facility-based	2012	200	147	Lymph node	Culture	
Birhanu et al [~~(~~18~~)~~]	2014	cross-sectional	Dessie	Facility-based	2012-2013	90	32	Lymph node	Culture	
Berg et al [~~(~~34~~)~~]	2015	cross-sectional	Gondar, Woldiya, Ghimbi, Fiche, and Butajira, Jinka and Filtu, AA	Facility-based	2006–2010	1198	456	Lymph node	Microscopy Culture	having regula[illegible] direct contact with live animals, low education level
Tadesse et al [~~(~~28~~)~~]	2015	cross-sectional	Jimma	Facility-based		143	88	Lymph node	Microscopy Culture Xpert MTB/RIF	
Korma et al [~~(~~23~~)~~]	2015	cross-sectional	Addis Ababa	Facility-based	2012 to 2013	200	116	pleural, peritoneal and synovial fluids	Culture	
Abdissa et al [~~(~~29~~)~~]	2015	cross-sectional	Jimma	Facility-based	2013	144	96	Lymph node	Microscopy Culture	

Formatted: Left

Formatted: Left

Formatted: Left

Fanosie et al [(19)]	2016	cross-sectional	Gonder	Facility-based	2015	141	37	Peritoneal fluid, CSF, Pleural fluid, lymph node	Culture<br>Xpert MTB/RIF	Adult patients, previous history of contact with known pulmonary TB, HIV positive
Zewdie et al [(24)]	2016	cross-sectional	Addis Ababa,	Facility-based	2013	206	74	Lymph node	Culture	
Mulu et al [(20)]	2017	cross-sectional	Debre Markos	Facility-based	2014 to 2015	182	53	Peritoneal, Pus, lymph node, pleural fluid	Xpert MTB/RIF	Retreated ,M[illegible] HIV positive –, Age 41-50
Metaferia et al [(21)]	2018	cross-–sectional	Dessie	Facility-based	2017	353	31	Peritoneal fluid, CSF, Pleural fluid, lymph node	Microscopy<br>Xpert MTB/RIF	Pprevious his[illegible] of PTB, contact with PTB patients
Tadesse et al [(30)]	2018	cross-sectional	Jimma	Facility-based	2015-2017	572	242	Lymph node, CSF, pleural, peritoneal, pericardial fluids.	Culture<br>Xpert MTB/RIF	
Fantahun et al [(25)]	2019	cross-sectional	Addis Ababa	Facility-based	2015-2016	152	75	Lymph node	Microscopy<br>Culture<br>Xpert MTB/RIF	
Tedla et al [(22)]	2019	cross-sectional	Dessie	Facility-based	2018	337	92	Peritoneal fluid, CSF, Pleural fluid, lymph node synovial fluid	Xpert MTB/RIF	HIV-positive, previous history of PTB
Assefa et al [(26)]	2021	cross-sectional	Addis Ababa	Facility-based	2020	211	50	Lymph node	Microscopy<br>Culture	

HIV-human immunodeficiency virus; MTB/RIF-Mycobacterium tuberculosis/ Rifampicin; PTB-pulmonary tuberculosis; TB-Tuberculosis;

~~2.8.~~ The pooled prevalence of bacteriologically confirmed EPTB

The frequency of EPTB varied ~~differed~~ widely over the 20 ~~between included~~ studies. The prevalence ranged ~~of EPTB was found to be ranges~~ from 9% [(21)] to 78% [(32)]. The pooled prevalence of bacteriologically confirmed EPTB was 43% (95%CI; 0.34–0.52, I^2; 98.45%, p-value < 0.01) according to random- –effects methodology. The highest EPTB prevalence was reported from Addis Ababa, Bar Dar, and Dire Dawa [(32)], with a rate of 78%, while the lowest was reported from Dessie [(21)], with a rate of 9% (Fig 2). The variety of distinct EPTB in

Formatted: Left

Formatted: Left

Formatted: Left

Formatted: Indent: Left: 0.25", No bullets or numbering

Ethiopia is depicted in a funnel plot (Fig~~.~~ 3). The graph depicted studies with fewer participants and events scattered throughout the pooled horizontal estimate, implying a greater influence due to chance.

Figure 2: Pooled proportion of any bacteriological positive of EPTB (Pooled proportion estimated using a random-effects model, *ES=effect size).

Figure 3: Funnel plot of ~~twenty used~~ studies for meta-analysis (ES-stand for standard error, ES-effect size: proportion of any bacteriologically confirmed of EPTB)

~~2.9.~~ Subgroup analysis by diagnostic testing methods

Formatted: Indent: Left: 0.25", No bullets or numbering

The pooled effect estimate on the prevalence of EPTB positive cases was based on a subgroup analysis of publications comparing culture, smear microscopy, and Xpert MTB/RIF assay methods. There was no heterogeneity among studies conducted in culture, smear microscopy, and Xpert MTB/RIF assay, according to the subgroup analysis performed by a diagnostic test. However, there is still heterogeneity among studies that look at multiple diagnostic tests. In culture, smear microscopy, and Xpert MTB/RIF assay diagnostic methods, the prevalence of pooled effect estimates was 49% (95%CI; 0.41–0.57, I^2 = 96.43%, p-value < 0.01) (see Fig~~.~~ 4), 22% (95%CI; 0.13–0.30, I^2 = 98.56%, p-value < 0.01) (see Fig~~.~~ 5~~6~~), 39% (95%CI; 0.23–0.54, I^2 = 98.73%, p-value < 0.01) (see Fig~~.~~ ~~8~~6) respectively.

Figure 4: Pooled proportion of culture-positive EPTB using random-effects model~~(Pooled proportion estimated using random-effect model, *ES=effect size~~).

Formatted: Font: (Default) +Body (Calibri), 9 pt, Bold, Font color: Accent 1

Figure 5: Pooled proportion of smear-positive EPTB using random-effects model~~(Pooled proportion estimated using random-effect model, *ES=effect size)~~.

Figure 6: Pooled proportion of Xpert MTB/RIF assay positive EPTB using random-effects model~~(Pooled proportion estimated using random-effect model, *ES=effect size)~~.

~~2.10.~~ Risk of bias across studies publication

Formatted: Indent: Left: 0.25", No bullets or numbering

The presence of publication bias was determined using a funnel plot. By showing funnel plots with the logarithms of effect size and their standard errors, we were able to quantify publication bias. Visual inspection revealed indications of publication bias for the majority of the culture diagnostic method estimates, with most studies clustered at the funnel's apex and a few spread to the extreme right and left corners (Fig 7). The funnel plots for Xpert MTB/RIF assay and smear microscopy methods were most studies clustered at the funnel's bottom and a few spread to the extreme right corners (Fig 8 and Fig 9).

Figure 7: Funnel plot of subgroup of 15 of the 20 selected studies~~fifteen used studies for meta-analysis (ES-stand for standard error, ES-effect size: proportion of culture-positive EPTB)~~

Figure 8: Funnel plot of subgroup of 11 of the 20 selected studies ~~eleven used studies for meta-analysis (ES-stand for standard error, ES-effect size: proportion of smear-positive EPTB)~~

Figure 9: Funnel plot of subgroup of 8 of the 20 selected studies ~~eight used studies for meta-analysis (ES-stand for standard error, ES-effect size: proportion of Xpert MTB/RIF assay positive EPTB)~~

~~2.11.~~ Associated risk factors of bacteriologically confirmed EPTB

Formatted: Font color: Auto

Formatted: Font: Not Bold

Formatted: Caption, Left, No bullets or numbering

The impact of each study on the overall meta-analysis summary estimate was investigated. ~~Having a~~A history of ~~previous~~ PTB infection and contact with PTB patients was found to be significant risk factors for EPTB incidence in the majority of investigations [~~(~~15, 16, 19-22~~)~~].

Furthermore, having regular and direct contact with live animals, as well as the consumption of raw milk, were found to be strongly related to the incidence of EPTB [(16, 34)]. Additionally, HIV-1 positive [(19, 20, 22)], ages <14 [(15)], age 41-50 [(20)], being male [(15, 20)], monthly less income [(16)], Urban [(15)] were also likely to be linked to the most common EPTB remark categories.

Discussion

This systematic review and meta-analysis ~~assessed~~estimated the pooled prevalence of bacteriologically confirmed EPTB and the associated risk factors among persons suspected to have non-respiratory tuberculosis /EPTB/ in Ethiopia ~~the prevalence of EPTB and of the associated risk factors~~ using published studies ~~conducted in Ethiopia~~ over the last two decades. This meta-analysis included a total of 5439 EPTB suspects from 20 studies published between 2003 and 2021. The pooled estimated prevalence of samples with any bacteriological evidence in the studies was 43% (95%CI; 0.34–0.52, I^2; 98.45%, p-value < 0.01). ~~Having a~~A previous history of ~~previous~~ PTB infection and contact with PTB patients, contact with live animals, consumption of raw milk, HIV-positive, ages <14 to 41-50, male, monthly less income, and living in an urban area with known EPTB patients were all found to be independently associated with EPTB in this study.

The overall pooled prevalence of bacteriological confirmed EPTB in this systematic review and meta-analysis data was 43%. This finding is approximately similar to that the previously reported from Cameroon [(35)]. In contrast, when compared to the estimated prevalence of EPTB among notified TB cases in Africa, this is a high figure. According to the 2017 WHO report, the prevalence of EPTB among notified TB cases in Africa and the rest of the globe was 16% and 15%, respectively [(7)]. Furthermore, a systematic~~systemic~~ review and meta-analysis of the

prevalence of EPTB among patients living with HIV/AIDS in Sub-Saharan Africa revealed a lower prevalence of EPTB than our findings [~~(~~36~~)~~]. However, in the current study the funnel plot revealed that there is publication bias, where among 20 studies included in this study, 10 were above 95% upper limit and 5 were below 95% lower limit and only 5 were within the CI. This might be due to the low number of studies conducted so far in Ethiopia and their variations in using the laboratory methods. Thus, this may under estimate the pooled bacteriologically confirmed EPTB prevalence among EPTB presumptive cases in Ethiopia.

In this current systematic review and meta-analysis investigation, HIV-1 infected patients were the most significant risk factors ~~that were~~ associated with~~/that explained~~ EPTB infection [~~(~~19, 20, 22~~)~~]. Similarly, several studies have examined the association between HIV-1 infection and EPTB infection [~~(~~6, 37~~)~~]. Furthermore, nearly half of EPTB patients were ~~developed~~ HIV-1 infected, according to a prior study [~~(~~38~~)~~]. This is due to the virus's immune deficiency condition, which allows the bacteria to spread from the primary infection site, the lung, to other parts of the body. During TB-HIV-1 co-infection, there is a lack of granuloma growth and functional disruption of the local immune response within the granuloma [~~(~~39~~)~~].

Our study showed that having a previous history of ~~previous~~ TB and history of contact with known pulmonary TB patient was found to be significant risk factors for EPTB development [~~(~~16, 19-22~~)~~]. Similarly, it is well known that patients with a previous history of anti-TB treatment cases have an increased risk of EPTB [~~(~~37~~)~~].

In this study, we also discovered that men had a higher prevalence of EPTB involvement than women [~~(~~15, 20~~)~~], which is consistent with similar findings in the previous study [~~(~~40~~)~~].

Formatted: Font: (Default) Times New Roman, 12 pt

However, another systematic review and meta-analysis study in Africa found that female patients with lymphadenitis were more common than male patients [(41)]. Likewise, women had a higher rate of EPTB infections than men [(42)]. In addition, another study reported the prevalence of EPTB was higher among females than males [(43)]. Only three databases were searched. This could lead to publication bias.

In the end, the current study had its own limitations. Firstly, the degree of EPTB prevalence in many parts of the country has yet to be addressed, making it impossible to conclude the true burden of EPTB in Ethiopia. Secondly, the observed publication bias that could be due to the differences in the laboratory methods might underestimate the estimated prevalence. Thirdly, Only three databases were searched. This could lead to publication bias. Finaly, there is high heterogeneity among studies that might affect the true estimates. However, the findings are still significant, because the rising rate of EPTB patients in the general population is concerning. In the end, the current study had its own limitations. First, the degree of EPTB prevalence in many parts of the country has yet to be addressed, making it impossible to conclude the true burden of EPTB in Ethiopia. Second, the statistical power of meta-analysis was reduced due to the poor quality of several of the studies included in the review and the variability of prevalence estimates between studies. The findings are still significant, however, because the rising rate of EPTB patients in the general population is concerning.

3. Conclusions

The finding of this study revealed that there is high bacteriologically confirmedprevalence of EPTB among persons suspected to have EPTB in Ethiopia. Patients having a history of previous tuberculosis, a poor income, a history of tuberculosis contact with a known PTB case, being

Formatted: Font: (Default) Times New Roman, 12 pt, Bold, Kern at 16 pt

Formatted: Normal, No bullets or numbering

HIV-1 positive, and having contact with PTB patients and a history of underlying diseases were all more likely to associated risk factors with EPTB. Thus, we recommend strengthening laboratory services for the diagnosis of EPTB in Ethiopia.

Acknowledgments

We acknowledge Ethiopian Public Health Institute for the access for article searching. We also acknowledge the authors of the original articles included in this systematic review and meta-analysis study.

Author Contributions

Conceptualization: Getu Diriba, Ayinalem Alemu.

Data curation: Getu Diriba, Habteyes Hailu Tola, Kirubel Eshetu, Ayinalem Alemu.

Formal analysis: Getu Diriba, Bazezew Yenew, Dinka Fekadu Gamtesa, Ayinalem Alemu.

Funding acquisition: Getu Diriba, Ayinalem Alemu.

Investigation: Getu Diriba, Habteyes Hailu Tola, Kirubel Eshetu, Bazezew Yenew, Ayinalem Alemu, Dinka Fekadu Gamtesa.

Validation: Kirubel Eshetu, Ayinalem Alemu.

Methodology: Getu Diriba, Ayinalem Alemu, Habteyes Hailu Tola, Kirubel Eshetu.

Software: Ayinalem Alemu, Dinka Fekadu Gamtesa.

Writing-original draft: Getu Diriba.

Writing – review & editing: Ayinalem Alemu, Habteyes Hailu Tola, Kirubel Eshetu.

Competing interests

There are no competing interests stated by the authors.

Data Availability Statement

All the important information is available within the manuscript and its supplementary files The manuscript contains all important information.

Funding

The author(s) received no specific funding for this work.

References

1. Prakasha SR, Suresh G, D'Sa I P, Shetty SS, Kumar SG. Mapping the pattern and trends of extrapulmonary tuberculosis. J Glob Infect Dis. 2013;5(2):54-9.
2. Adada H, Valley MA, Nour SA, Mehta J, Byrd RP, Jr., Anderson JL, *et al.* Epidemiology of extra-pulmonary tuberculosis in the United States: high rates persist in the post-HIV era. Int J Tuberc Lung Dis. 2014;18(12):1516-21.
3. World Health Organization, Global Tuberculosis Report 2018.
4. Yang D, Kong Y. The bacterial and host factors associated with extrapulmonary dissemination of Mycobacterium tuberculosis. Front Biol (Beijing). 2015;10(3):252-61.
5. Ramirez-Lapausa M M-SAA-A. Extrapulmonary tuberculosis : an overview. . Rev Esp Sanid Penit 2015;17:3-11.
6. Pollett S, Banner P, O'Sullivan MV, Ralph AP. Epidemiology, Diagnosis and Management of Extra-Pulmonary Tuberculosis in a Low-Prevalence Country: A Four Year Retrospective Study in an Australian Tertiary Infectious Diseases Unit. PLoS One. 2016;11(3):e0149372.
7. World Health Organization Global tuberculosis report 2017.

Formatted: Font: Italic

8. Purohit M, Mustafa T. Laboratory Diagnosis of Extra-pulmonary Tuberculosis (EPTB) in Resource-constrained Setting: State of the Art, Challenges and the Need. J Clin Diagn Res. 2015;9(4):EE01-6.
9. Parsons LM, Somoskovi A, Gutierrez C, Lee E, Paramasivan CN, Abimiku A, *et al*. Laboratory diagnosis of tuberculosis in resource-poor countries: challenges and opportunities. Clin Microbiol Rev. 2011;24(2):314-50.
10. Lee JY. Diagnosis and treatment of extrapulmonary tuberculosis. Tuberc Respir Dis (Seoul). 2015;78(2):47-55.
11. Definitions and reporting framework for tuberculosis 2013 revision. 2013.
12. Lo CK, Mertz D, Loeb M. Newcastle-Ottawa Scale: comparing reviewers' to authors' assessments. BMC Med Res Methodol. 2014;14:45.
13. Rucker G, Schwarzer G, Carpenter JR, Schumacher M. Undue reliance on I(2) in assessing heterogeneity may mislead. BMC Med Res Methodol. 2008;8:79.
14. Huedo-Medina TB, Sanchez-Meca J, Marin-Martinez F, Botella J. Assessing heterogeneity in meta-analysis: Q statistic or I2 index? Psychol Methods. 2006;11(2):193-206.
15. Biadglegne F, Tesfaye W, Sack U, Rodloff AC. Tuberculous lymphadenitis in Northern Ethiopia: in a public health and microbiological perspectives. PLoS One. 2013;8(12):e81918.
16. Zenebe Y, Anagaw B, Tesfay W, Debebe T, Gelaw B. Smear positive extra pulmonary tuberculosis disease at University of Gondar Hospital, Northwest Ethiopia. BMC Res Notes. 2013;6:21.

Formatted: Font: Italic

17. Garedew L, Mihret A, Ameni G. Molecular typing of mycobacteria isolated from extrapulmonary tuberculosis patients at Debre Birhan Referral Hospital, central Ethiopia. Scand J Infect Dis. 2013;45(7):512-8.

18. Birhanu T BM, Ameni G. Molecular Characterization of Mycobacterium tuberculosis Complex Isolated from Tuberculous Lymphadenitis Patients at Dessie Private Hospitals, Northern Ethiopia. Journal of Biology, Agriculture and Healthcare. 2014;4(5).

19. Fanosie A, Gelaw B, Tessema B, Tesfay W, Admasu A, Yitayew G. Mycobacterium tuberculosis Complex and HIV Co-Infection among Extrapulmonary Tuberculosis Suspected Cases at the University of Gondar Hospital, Northwestern Ethiopia. PLoS One. 2016;11(3):e0150646.

20. Mulu W, Abera B, Yimer M, Hailu T, Ayele H, Abate D. Rifampicin-resistance pattern of Mycobacterium tuberculosis and associated factors among presumptive tuberculosis patients referred to Debre Markos Referral Hospital, Ethiopia: a cross-sectional study. BMC Res Notes. 2017;10(1):8.

21. Metaferia Y, Seid A, Fenta GM, Gebretsadik D. Assessment of Extrapulmonary Tuberculosis Using Gene Xpert MTB/RIF Assay and Fluorescent Microscopy and Its Risk Factors at Dessie Referral Hospital, Northeast Ethiopia. Biomed Res Int. 2018;2018:8207098.

22. Tedla E, Ayalew, G. Mycobacterium tuberculosis burden, multidrug resistance pattern, and associated risk factors among presumptive extrapulmonary tuberculosis cases at Dessie Referral Hospital, Northeast Ethiopia. The Egyptian Journal of Chest Diseases and Tuberculosis. 2020;69(3):449.

23. Korma W, Mihret A, Hussien J, Anthony R, Lakew M, Aseffa A. Clinical, molecular and drug sensitivity pattern of mycobacterial isolates from extra-pulmonary tuberculosis cases in Addis Ababa, Ethiopia. BMC Infect Dis. 2015;15:456.

24. Zewdie O, Mihret A, Ameni G, Worku A, Gemechu T, Abebe T. Molecular typing of mycobacteria isolated from tuberculous lymphadenitis cases in Addis Ababa, Ethiopia. Int J Tuberc Lung Dis. 2016;20(11):1529-34.

25. Fantahun M, Kebede A, Yenew B, Gemechu T, Mamuye Y, Tadesse M, *et al.* Diagnostic accuracy of Xpert MTB/RIF assay and non-molecular methods for the diagnosis of tuberculosis lymphadenitis. PLoS One. 2019;14(9):e0222402.

26. Assefa G, Desta K, Araya S, Girma S, Mihret A, Hailu T, *et al.* Diagnostic efficacy of Light-Emitting Diode (LED) Fluorescence based Microscope for the diagnosis of Tuberculous lymphadenitis. PLoS One. 2021;16(7):e0255146.

27. Abdissa K, Tadesse M, Bezabih M, Bekele A, Apers L, Rigouts L, *et al.* Bacteriological methods as add on tests to fine-needle aspiration cytology in diagnosis of tuberculous lymphadenitis: can they reduce the diagnostic dilemma? BMC Infect Dis. 2014;14:720.

28. Tadesse M, Abebe G, Abdissa K, Aragaw D, Abdella K, Bekele A, *et al.* GeneXpert MTB/RIF Assay for the Diagnosis of Tuberculous Lymphadenitis on Concentrated Fine Needle Aspirates in High Tuberculosis Burden Settings. PLoS One. 2015;10(9):e0137471.

29. Abdissa K, Tadesse M, Abdella K, Bekele A, Bezabih M, Abebe G. Diagnostic performance of fluorescent light-emitting diode microscopy for tuberculous lymphadenitis in a high-burden setting. Trop Med Int Health. 2015;20(11):1543-8.

30. Tadesse M, Abebe G, Bekele A, Bezabih M, Yilma D, Apers L, *et al.* Xpert MTB/RIF assay for the diagnosis of extrapulmonary tuberculosis: a diagnostic evaluation study. Clin Microbiol Infect. 2018;25(8):1000-5.

31. Yassin MA, Olobo JO, Kidane D, Negesse Y, Shimeles E, Tadesse A, *et al.* Diagnosis of tuberculous lymphadenitis in Butajira, rural Ethiopia. Scand J Infect Dis. 2003;35(4):240-3.

32. Iwnetu R, van den Hombergh J, Woldeamanuel Y, Asfaw M, Gebrekirstos C, Negussie Y, *et al.* Is tuberculous lymphadenitis over-diagnosed in Ethiopia? Comparative performance of diagnostic tests for mycobacterial lymphadenitis in a high-burden country. Scand J Infect Dis. 2009;41(6-7):462-8.

33. Derese Y HE, Assefa T, Bekele Y, Mihret A, Aseffa A. Comparison of PCR with standard culture of fine needle aspiration samples in the diagnosis of tuberculosis lymphadenitis. J Infect Dev Ctries 2012;6(1):53-7.

34. Berg S, Schelling E, Hailu E, Firdessa R, Gumi B, Erenso G, *et al.* Investigation of the high rates of extrapulmonary tuberculosis in Ethiopia reveals no single driving factor and minimal evidence for zoonotic transmission of Mycobacterium bovis infection. BMC Infect Dis. 2015;15:112.

35. Namme L, Achu J, Christopher K, Elvis T, Hugo Bertrand M, Marie-Solange D. Extrapulmonary tuberculosis and HIV coinfection in patients treated for tuberculosis at the Douala General Hospital in Cameroon. Annals of Tropical Medicine and Public Health. 2013;6(1):100.

Formatted: Font: Italic

Formatted: Font: Italic

Formatted: Font: Italic

Formatted: Font: Italic

36. Mohammed H, Assefa N, Mengistie B. Prevalence of extrapulmonary tuberculosis among people living with HIV/AIDS in sub-Saharan Africa: a systemic review and meta-analysis. HIV AIDS (Auckl). 2018;10:225-37.

37. Qian X, Nguyen DT, Lyu J, Albers AE, Bi X, Graviss EA. Risk factors for extrapulmonary dissemination of tuberculosis and associated mortality during treatment for extrapulmonary tuberculosis. Emerg Microbes Infect. 2018;7(1):102.

38. Arega B, Mersha A, Minda A, Getachew Y, Sitotaw A, Gebeyehu T, *et al.* Epidemiology and the diagnostic challenge of extra-pulmonary tuberculosis in a teaching hospital in Ethiopia. PLoS One. 2020;15(12):e0243945.

39. Diedrich CR, Flynn JL. HIV-1/mycobacterium tuberculosis coinfection immunology: how does HIV-1 exacerbate tuberculosis? Infect Immun. 2011;79(4):1407-17.

40. Yone EW, Kengne AP, Moifo B, Kuaban C. Prevalence and determinants of extrapulmonary involvement in patients with pulmonary tuberculosis in a Sub-Saharan African country: a cross-sectional study. Scand J Infect Dis. 2013;45(2):104-11.

41. Mekonnen D, Derbie A, Abeje A, Shumet A, Nibret E, Biadglegne F, *et al.* Epidemiology of tuberculous lymphadenitis in Africa: A systematic review and meta-analysis. PLoS One. 2019;14(4):e0215647.

42. Lin CY, Chen TC, Lu PL, Lai CC, Yang YH, Lin WR, *et al.* Effects of gender and age on development of concurrent extrapulmonary tuberculosis in patients with pulmonary tuberculosis: a population based study. PLoS One. 2013;8(5):e63936.

43. R E, M N. Epidemiological profile among pulmonary and extrapulmonary tuberculosis patients in Laayoune, Morocco. Pan African Medical Journal. 2020;37(56).

Supporting documents

S1: File: PRISMA checklist

S2: File: Literature search strategy from searched databases

S3: File: Detailed data of the included studies

S4: File: Newcastle-Ottawa quality assessment scale for cross sectional studies

Ref: PONE-D-22-00257

Bacteriologically confirmed extrapulmonary tuberculosis and the associated risk factors among extrapulmonary tuberculosis suspected patients in Ethiopia: A systematic review and meta-analysis

Dear Editor,

Thank you for your informative comments and thoughtful comments on our manuscript. We have addressed your and the reviewers' comments one by one. We also appreciate the reviewers for their critical observation and informative comments which radically improved our manuscript quality. We have provided point-by-point responses to the reviewers' comments below. Also, we would like to inform you that we have used a hyperlink to indicate where we made changes in the previous version of the manuscript based on the reviewers' comments.

Journal Requirements:

When submitting your revision, we need you to address these additional requirements.

1. Please ensure that your manuscript meets PLOS ONE's style requirements, including those for file naming. The PLOS ONE style templates can be found at

https://journals.plos.org/plosone/s/file?id=wjVg/PLOSOne_formatting_sample_main_body.pdf and https://journals.plos.org/plosone/s/file?id=ba62/PLOSOne_formatting_sample_title_authors_affiliations.pdf

Response: Thank you for the comment. The manuscript was prepared according to PLOS ONE's style requirements.

2. Please provide additional details regarding participant consent. In the ethics statement in the Methods and online submission information, please ensure that you have specified (1) whether consent was informed and (2) what type you obtained (for instance, written or verbal, and if verbal, how it was documented and witnessed). If your study included minors, state whether you obtained consent from parents or guardians. If the need for consent was waived by the ethics committee, please include this information.

If you are reporting a retrospective study of medical records or archived samples, please ensure that you have discussed whether all data were fully anonymized before you accessed them and/or whether the IRB or ethics committee waived the requirement for informed consent. If patients provided informed written consent to have data from their medical records used in research, please include this information.

Response: Thank you for the valuable comment. In the revised manuscript we included a sentence about ethical consideration “Since this study is based on previously published articles ethical approval is not applicable”.

3. Thank you for stating the following financial disclosure:

“The funders had no role in study design, data collection and analysis, decision to publish, or preparation of the manuscript.”

At this time, please address the following queries:

a) Please clarify the sources of funding (financial or material support) for your study. List the grants or organizations that supported your study, including funding received from your institution.

b) State what role the funders took in the study. If the funders had no role in your study, please state: “The funders had no role in study design, data collection and analysis, decision to publish, or preparation of the manuscript.”

c) If any authors received a salary from any of your funders, please state which authors and which funders.

d) If you did not receive any funding for this study, please state: “The authors received no specific funding for this work.”

Please include your amended statements within your cover letter; we will change the online submission form on your behalf.

Response: Thank you. This work did not receive any funding for this study and we have already stated it in the first submission before the reference section “The author(s) received no specific funding for this work”

4. We note that you have stated that you will provide repository information for your data at acceptance. Should your manuscript be accepted for publication, we will hold it until you provide the relevant accession numbers or DOIs necessary to access your data. If you wish to make changes to your Data Availability statement, please describe these changes in your cover letter and we will update your Data Availability statement to reflect the information you provide.

Response: Thank you. All the important information is available within the manuscript and its supplementary files.

5. Please ensure that you refer to Figure 5 in your text as, if accepted, production will need this reference to link the reader to the figure.

Response: Thank you for your critical observation and comment; we revised as per the suggestion.

Additional Editor Comments:

Both reviewers felt that the paper had merit. Both did however identify concerns with the methodology. Specifically, there was a concern that the denominator of the study (all PTB cases) was not realistic and undefined and 1 reviewer felt strongly that the review question needed to be tightened. There were also concerns with the construction of the forest plots which reviewer 1 felt should be ordered from low to high. Both reviewers highlighted a number of small stylistic errors which should also be corrected.

Response: Thank you for your suggestions and constructive comments. We have addressed your comments across the manuscript methodology. All the forest plots are arranged from low to high. Also, all technical errors have been corrected.

Review Comments to the Author

Reviewer #1

22 lack of a strong…

Response: Thank you, we revised it accordingly.

24 insert full stop

Response: Thank you, we corrected it as per the suggestion.

27 spread across

Response: Thank you for your suggestion. We have corrected it.

29 insert full stop

Response: Thank you for your suggestion. We inserted a full stop in the revised manuscript.

31 “majority”: what is the percentage?

Response: Thank you for the question. At this stage, we revised the result section of the abstract.

31 substitute “articles” for “research”

Response: Thank you for your suggestion. We revised it accordingly.

32 variations

Response: Thank you. We have corrected it.

33 “medium/high risk”: the funnel plot in fig 3 for the 20 papers shows 10 papers above the 95% upper limit and 5 below the 95% lower limit and only 5 within the CI. More discussion is required for your assessment. Is this justified by the standard error of the effect size on the y-axis?

Response: Thank you for the valuable comment. Now we included a detailed paragraph in the discussion section that described it.

34 Delete “having”. PTB must be in full here, that is, “pulmonary tuberculosis”

Response: Thank you for the valuable comment. Now, defined it in full first and then we used the abbreviation.

35 Delete “being”

Response: Thank you. We have deleted the comment

41 Keywords:… “Pulmonary Tuberculosis” for Tuberculosis…

Response: Thank you for the suggestion. We revised it.

49 Use “EPTB”; this has already been defined

Response: Thank you. In the current version, we used EPTB.

54 EPTB

Response: Thank you for your suggestion. We have corrected it.

55 15%

Response: Thank you for your comment. We have added the % according to the comment

61 Delete “and”

Response: Thank you for your suggestion. We have deleted it.

62 Delete extra space after “resource- “

Response: Thank you. We have deleted the extra space.

64 turnaround times

Response: Thank you for your suggestion. We corrected it accordingly.

70 “had started” for “have been started”

Response: Thank you for your suggestion. We revised it as per the given suggestion.

72 (11) for [11]

Response: Thank you for the suggestion. We revised the format for the whole referencing style based on the *Plos One* journal requirements.

76 et seq. Remove end full stops in numbering, for example, 2. and 2.1. should be 2 and 2.1

Response: Thank you for your suggestion. In the revised version, we have removed the numbers in the entire manuscript according to the Journal’s criteria.

86 Delete “the”

Response: Thank you for your suggestion. We deleted it.

88 Delete “language”

Response: Thank you for your suggestion. We deleted it.

93 “using a Microsoft Excel worksheet” should be “Microsoft Excel® 2016” (or whatever version was used)

Response: Thank you for your suggestion. We have revised it accordingly.

96 “resolved inconsistencies that arose”

Response: Thank you for your suggestion. We revised it as per the suggestion

99-102 The WHO definition of a positive test result was applied. This states that a positive diagnostic test result using smear microscopy, culture, Xpert MTM/RIF or nucleic acid amplification test is a bacteriological confirmation of an EPTB infection.

Response: Thank you for the valuable comment and suggestion. We have revised it as per the given suggestion.

104 Delete “included”

Response: Thank you, we have deleted it.

105-107 Persistent disagreements indicate a lack of clarity in applying the Newcastle-Ottawa scale. Should the scale be modified and would different interpretations be applied by researchers on similar studies. The nature of the disagreements and outcomes of using alternative interpretations should be discussed especially in light of the small sample of 20 papers

Response: Thank you; we have addressed this according to the comment

112 STATA® 14.2 StataCorp LLC, Texas, USA

Response: Thank you, we revised it accordingly.

113 a 95% confidence interval. Random-effects

Response: We have changed according to the comment

114 The ‘metaprop’ command in STATA was used.

Response: Thank you, we revised it accordingly.

115-116 A forest plot shows distributional information not prevalence

Response: Thank you, it is revised accordingly.

119-127 This requires the editing out of excess detail in light of the PRISMA diagram. The PRISMA diagram is important and sets out clearly the process in reducing the 938 articles to 20 used in this study 123 The 36 non-Ethiopian papers should either be included as controls in the forest plots or in separate forest plots. These could indicate possible biases, inconsistencies or differences in interpretation of data in Ethiopian papers (for example see disagreements in 105-107 above). This applies to funnel plots as well

Response: Thank you, we revised it as per the suggestion.

135 Delete “was”

Response: We have deleted it.

142 Delete “another”

Response: We have deleted it.

Page 8 Some hyphens missing in “cross-sectional”. Associated factors column requires some editing (spaces and spacing of commas)

Response: Thank you, we revised it accordingly.

148 The frequency of EPFB varied widely over the 20 studies. The prevalence ranged from 9% (21) to 78% (32). See also general comment below on superscript of paper reference.

Response: Thank you for the comment. The numbers in the bracket are the references, now we revised the referencing based on the journal’s criteria or using [ ].

150 random-effects

Response: We have changed according to the comment

157 using a random-effects model

Response: We have corrected accordingly.

157 “Effect size” should appear in full in the x-axis label

Response: Thank you. We have addressed the comment

158 “twenty studies”, delete used.

Response: We have deleted it.

158-159 Standard error of effect size should appear in full on the y-axis

Response: Thank you for the suggestion, we corrected it accordingly.

160 The subgroups of the 20 studies should be shown in the PRISMA diagram to ensure completeness and intelligibility

Response: Thank you for the suggestion. We sub-grouped the included studies based on the laboratory method used, and we described it in the flow diagram and early in the result section.

170 Pooled proportion of culture-positive EPTB using random-effects model

Response: Thank you, we revised it as per the suggestion.

172 Same style as 170

Response: We have changed according to the comment

174 Same style as 170

Response: We have changed according to the comment

184 Funnel plot of subgroup of 15 of the 20 selected studies

Response: We have changed according to the comment

184 Standard error of effect size and effect size should appear in the axis labels

Response: Thank you for the valuable comment; we revised accordingly per the suggestion. and effect size according to the comment

186 Same style as 184

Response: Thank you, we revised it accordingly.

188 Same style as 184

Response: Thank you, we revised it accordingly.

192 A history of PTB infection

Response: Thank you, we corrected it.

200 Delete “of”

Response: Thank you we have deleted it.

196 HIV should be HIV-1

Response: Thank you for the suggestion, we revised it accordingly.

204 A history of PTB infection ("history" implies previous history)

Response: We have corrected per the suggestion.

210 … similar to that…

Response: We revised it.

214 “systemic” should be “systematic”

Response: Thank you, now it is corrected.

217 HIV-1

Response: Thank you for the suggestion, we revised it accordingly.

218 …risk factors associated with EPTB infection…

Response: Thank you, we revised it as per the suggestion.

219 HIV-1

Response: Thank you, now it is revised accordingly.

220 … EPTB patients were HIV-1 infected,…

Response: Thank you. We revised as per the suggestion.

223 TB-HIV-1 co-infection

Response: Thank you. We revised it accordingly.

227 … with a history of… ("history" implies previous history)

Response: We have revised it accordingly.

235 Firstly,

Response: Thank you, we revised it.

237 Secondly,

Response: Thank you, we revised it.

238 Statistical power and its reduction has not been discussed in the paper. What is the estimate of the reduction and has this invalidated the paper? (Does statistical power here refer to I^2, p-value or items outside the 95% pseudo-confidence gradients in the funnel plots? 238 "poor quality of several studies" – I am not sure this has been sufficiently discussed (if at all)

Response: Thank you for the valuable comment. Now, we revised the limitation of the study as follows "In the end, the current study had its limitations. Firstly, the degree of EPTB prevalence in many parts of the country has yet to be addressed, making it impossible to conclude the true burden of EPTB in Ethiopia. Secondly, the observed publication bias that could be due to the differences in the laboratory methods might underestimate the estimated prevalence. Thirdly, there is high heterogeneity among studies that might affect the true estimates. However, the findings are still significant, because the rising rate of EPTB patients in the general population is concerning"

244 HIV-1

Response: We revised it accordingly.

General: number references to papers should preferably be shown as superscripts to avoid with number in the paper. For example, line 49, …region (8%) (3) should be …region (8%)3 (3 as superscript)

Response: Thank you for the valuable comment and suggestion. Now we revised all the references according to the PloS One guideline, Such that we used the square brackets "[ ]".

References: Journal references should be italicised. Latinisms like et.al. should be italicized

Response: Thank you, we revised as per the suggestion.

Figure 1: PRISMA diagram should show the subgroups budding off the "Final analysis" box. Reference to this in the main body of the paper will simplify some of the wording in the main body

Response: Thank you for the suggestion, as we have stated in the previous response we revised the flow diagram according to the PRISMA guideline.

Figure 2: Forest plot should be sorted by ES and show ranking from lowest to highest ES. Metaferia et. al. would be at the bottom and Iwnetu et.al. at the top

Response: Thank you for the comment, we revised it accordingly.

Figure 3: Axis-labels to be described in full

Response: We have described in full according to the comment

Figure 4: Sort the publications as in figure 2

Response: Thank you, we revised it accordingly.

Figure 5: Sort the publications as in figure 2

Response: Thank you, we revised it accordingly.

Figure 6: Sort the publications as in figure 2

Response: Thank you, we revised it accordingly.

Figures 7, 8 and 9 – same as in figure 3

Response: Thank you, we revised it accordingly.

Reviewer #2:

This is a well written article, and findings will be helpful to Ethiopian health care workers, and others working in a LMIC/African context.

However, I have a difficulty with the foundational methodology (meta-analysis) of the study.
o It is not stated what the research questions for the descriptive studies of EPTB which were included in the meta-analysis were.

o Studies which were included could have identified EPTB as a subset of all PTB, or they may have described EPTB alone, from persons with clinical criteria matching only those in whom EPTB is suspected.
o Without specifying which approach the authors of this meta-analysis wanted de novo, the

selection criteria results in a wide range of approaches to EPTB diagnosis being selected, which will lead to enormous heterogeneity, and lack of meaningful comparison.

Before conducting a meta-analysis, it is critical to given to formulate the review question clearly. I think the review question ‘to investigate the prevalence of EPTB in Ethiopia’ is too broad. It will be helpful to say ‘Investigate the prevalence of EPTB amongst persons with non-pulmonary TB’. The results are then clearly applicable to persons in whom PTB has been excluded.

As the current aim stands, it implies that that the aim is to identify the burden of TB amongst all TB cases in Ethiopia. This begs the question of a denominator. Assessing the prevalence of EPTB requires that one knows the total burden of TB. How is it possible to assess the prevalence by identifying only studies that described EPTB? These studies would have identified persons who were suspected of EPTB as the starting point. Equally, an ascertainment of risk factors can only be done by comparison with non-EPTB groups.

Without narrowing the research question, it is not meaningful to present or interpret Forest plots.

Response: Thank you for the pertinent comments and valuable suggestions. This study is designed to investigate/estimate/ the prevalence of bacteriologically confirmed EPTB among individuals who are presumptive to have EPTB. Thus, the denominator is the number of EPTB or non-respiratory TB presumptive individuals, while the numerator is the number of bacteriologically confirmed EPTB presumptive individuals. Now we described in detail the research questions and the objectives in the revised manuscript. We revised the abstract section, the objectives at the end of the introduction section, and the first paragraph in the discussion section.

Other minor comments

A small consideration re methodology, search strategy – are there articles published by Ethiopians in literature that would not be listed in pubmed, Science Direct or on google scholar?

Response: Thank you very much for your informative comments. The majority of the articles published from Ethiopia are open access, because Ethiopia is among the low-income countries

which waived article processing charges. Thus, all open access articles can be accessed through Google Scholar research. In addition, Since we cannot freely access other databases in Ethiopia we could not search them. To address this comment we have added a sentence that indicates as our search is not comprehensive in limitation.

Line 49 Extrapulmonary Tuberculosis – 'tuberculosis' should be lower case.

Response: Thank you for the comment, we revised it accordingly.

Line 64 Turnaround Time – T should be lower case,

Response: Thank you, we revised it as per the suggestion.

Line 70 Anti-TB medicine – rather 'anti-tuberculosis therapy'

Response: Thank you, we revised it accordingly.

Line 108 The NewCastle Ottowa quality assessment scale is a checklist for cohort and case control studies. What motivated the use of this scale, over and above the PRISMA guidelines which are sufficient for meta-analyses and systematic reviews.

Response: Thank you for the valuable question. This systematic review and meta-analysis were conducted following the PRISMA guidelines. We used the Newcastle-Ottawa quality assessment checklist to assess the quality of individuals studies based on the questions available in the checklist as the Joanna Briggs critical appraisal tool. Since all studies included in the current study are cross-sectional studies we have used the Newcastle-Ottawa quality assessment scale adapted for cross-sectional studies.

Yours, Sincerely

Getu Diriba
