# Supplementary material for: Bacteriologically confirmed extrapulmonary tuberculosis and the associated risk factors among extrapulmonary tuberculosis suspected patients in Ethiopia: A systematic review and meta-analysis
Source: PLoS One. 2022 Nov 23;17(11):e0276701. doi: 10.1371/journal.pone.0276701 (PMC9683558; doi:10.1371/journal.pone.0276701)
Supplement: Attachment — Submitted filename: Response to Reviewers.docx [file pone.0276701.s005.docx]

**Ref: PONE-D-22-00257**

**Bacteriologically confirmed extrapulmonary tuberculosis and the associated risk factors among extrapulmonary tuberculosis suspected patients in Ethiopia: A systematic review and meta-analysis**

Dear Editor,

Thank you for your informative comments and thoughtful comments on our manuscript. We have addressed your and the reviewers' comments one by one. We also appreciate the reviewers for their critical observation and informative comments which radically improved our manuscript quality. We have provided point-by-point responses to the reviewers' comments below. Also, we would like to inform you that we have used a hyperlink to indicate where we made changes in the previous version of the manuscript based on the reviewers’ comments.

**Journal Requirements:**

When submitting your revision, we need you to address these additional requirements.

1. Please ensure that your manuscript meets PLOS ONE's style requirements, including those for file naming. The PLOS ONE style templates can be found at

<https://journals.plos.org/plosone/s/file?id=wjVg/PLOSOne_formatting_sample_main_body.pdf> and <https://journals.plos.org/plosone/s/file?id=ba62/PLOSOne_formatting_sample_title_authors_affiliations.pdf>

**Response:** Thank you for the comment. The manuscript was prepared according to PLOS ONE's style requirements.

2. Please provide additional details regarding participant consent. In the ethics statement in the Methods and online submission information, please ensure that you have specified (1) whether consent was informed and (2) what type you obtained (for instance, written or verbal, and if verbal, how it was documented and witnessed). If your study included minors, state whether you obtained consent from parents or guardians. If the need for consent was waived by the ethics committee, please include this information.

If you are reporting a retrospective study of medical records or archived samples, please ensure that you have discussed whether all data were fully anonymized before you accessed them and/or whether the IRB or ethics committee waived the requirement for informed consent. If patients provided informed written consent to have data from their medical records used in research, please include this information.

**Response:** Thank you for the valuable comment. In the revised manuscript we included a sentence about ethical consideration “Since this study is based on previously published articles ethical approval is not applicable”.

3. Thank you for stating the following financial disclosure:

“The funders had no role in study design, data collection and analysis, decision to publish, or preparation of the manuscript.”

At this time, please address the following queries:

a)        Please clarify the sources of funding (financial or material support) for your study. List the grants or organizations that supported your study, including funding received from your institution.

b)        State what role the funders took in the study. If the funders had no role in your study, please state: “The funders had no role in study design, data collection and analysis, decision to publish, or preparation of the manuscript.”

c)        If any authors received a salary from any of your funders, please state which authors and which funders.

d)        If you did not receive any funding for this study, please state: “The authors received no specific funding for this work.”

Please include your amended statements within your cover letter; we will change the online submission form on your behalf.

**Response:** Thank you. This work did not receive any funding for this study and we have already stated it in the first submission before the reference section “The author(s) received no specific funding for this work”

4. We note that you have stated that you will provide repository information for your data at acceptance. Should your manuscript be accepted for publication, we will hold it until you provide the relevant accession numbers or DOIs necessary to access your data. If you wish to make changes to your Data Availability statement, please describe these changes in your cover letter and we will update your Data Availability statement to reflect the information you provide.

**Response:** Thank you. All the important information is available within the manuscript and its supplementary files.

5. Please ensure that you refer to Figure 5 in your text as, if accepted, production will need this reference to link the reader to the figure.

**Response:** Thank you for your critical observation and comment; we revised as per the suggestion.

**Additional Editor Comments:**

Both reviewers felt that the paper had merit. Both did however identify concerns with the methodology. Specifically, there was a concern that the denominator of the study (all PTB cases) was not realistic and undefined and 1 reviewer felt strongly that the review question needed to be tightened. There were also concerns with the construction of the forest plots which reviewer 1 felt should be ordered from low to high. Both reviewers highlighted a number of small stylistic errors which should also be corrected.

**Response:** Thank you for your suggestions and constructive comments. We have addressed your comments across the manuscript methodology. All the forest plots are arranged from low to high. Also, all technical errors have been corrected.

**Review Comments to the Author**

**Reviewer #1**

22 lack of a strong…

**Response:** Thank you, we revised it accordingly.

24 insert full stop

**Response:** Thank you, we corrected it as per the suggestion.

27 spread across

**Response:** Thank you for your suggestion. We have corrected it.

29 insert full stop

**Response:** Thank you for your suggestion. We inserted a full stop in the revised manuscript.

31 “majority”: what is the percentage?

**Response:** Thank you for the question. At this stage, we revised the result section of the abstract.

31 substitute “articles” for “research”

**Response:** Thank you for your suggestion. We revised it accordingly.

32 variations

**Response:** Thank you. We have corrected it.

33 “medium/high risk”: the funnel plot in fig 3 for the 20 papers shows 10 papers above the 95% upper limit and 5 below the 95% lower limit and only 5 within the CI. More discussion is required for your assessment. Is this justified by the standard error of the effect size on the y-axis?

**Response:** Thank you for the valuable comment. Now we included a detailed paragraph in the discussion section that described it.

34 Delete “having”. PTB must be in full here, that is, “pulmonary tuberculosis”

**Response:** Thank you for the valuable comment. Now, defined it in full first and then we used the abbreviation.

35 Delete “being”

**Response:** Thank you. We have deleted the comment

41 Keywords:… “Pulmonary Tuberculosis” for Tuberculosis…

**Response:** Thank you for the suggestion. We revised it.

49 Use “EPTB”; this has already been defined

**Response:** Thank you. In the current version, we used EPTB.

54 EPTB

**Response:** Thank you for your suggestion. We have corrected it.

55 15%

**Response:** Thank you for your comment. We have added the % according to the comment

61 Delete “and”

**Response:** Thank you for your suggestion. We have deleted it.

62 Delete extra space after “resource- “

**Response:** Thank you. We have deleted the extra space.

64 turnaround times

**Response:** Thank you for your suggestion. We corrected it accordingly.

70 “had started” for “have been started”

**Response:** Thank you for your suggestion. We revised it as per the given suggestion.

72 (11) for [11]

**Response:** Thank you for the suggestion. We revised the format for the whole referencing style based on the *Plos One* journal requirements.

76 et seq. Remove end full stops in numbering, for example, 2. and 2.1. should be 2 and 2.1

**Response:** Thank you for your suggestion. In the revised version, we have removed the numbers in the entire manuscript according to the Journal’s criteria.

86 Delete “the”

**Response:** Thank you for your suggestion. We deleted it.

88 Delete “language”

**Response:** Thank you for your suggestion. We deleted it.

93 “using a Microsoft Excel worksheet” should be “Microsoft Excel® 2016” (or whatever version was used)

**Response:** Thank you for your suggestion. We have revised it accordingly.

96 “resolved inconsistencies that arose”

**Response:** Thank you for your suggestion. We revised it as per the suggestion

99-102 The WHO definition of a positive test result was applied. This states that a positive diagnostic test result using smear microscopy, culture, Xpert MTM/RIF or nucleic acid amplification test is a bacteriological confirmation of an EPTB infection.

**Response:** Thank you for the valuable comment and suggestion. We have revised it as per the given suggestion.

104 Delete “included”

**Response:** Thank you, we have deleted it.

105-107 Persistent disagreements indicate a lack of clarity in applying the Newcastle-Ottawa scale. Should the scale be modified and would different interpretations be applied by researchers on similar studies. The nature of the disagreements and outcomes of using alternative interpretations should be discussed especially in light of the small sample of 20 papers

**Response:** Thank you; we have addressed this according to the comment

112 STATA® 14.2 StataCorp LLC, Texas, USA

**Response:** Thank you, we revised it accordingly.

113 a 95% confidence interval. Random-effects

**Response:** We have changed according to the comment

114 The ‘metaprop’ command in STATA was used.

**Response:** Thank you, we revised it accordingly.

115-116 A forest plot shows distributional information not prevalence

**Response:** Thank you, it is revised accordingly.

119-127 This requires the editing out of excess detail in light of the PRISMA diagram. The PRISMA diagram is important and sets out clearly the process in reducing the 938 articles to 20 used in this study 123 The 36 non-Ethiopian papers should either be included as controls in the forest plots or in separate forest plots. These could indicate possible biases, inconsistencies or differences in interpretation of data in Ethiopian papers (for example see disagreements in 105-107 above). This applies to funnel plots as well

**Response:** Thank you, we revised it as per the suggestion.

135 Delete “was”

**Response:** We have deleted it.

142 Delete “another”

**Response:** We have deleted it.

Page 8 Some hyphens missing in “cross-sectional”. Associated factors column requires some editing (spaces and spacing of commas)

**Response:** Thank you, we revised it accordingly.

148 The frequency of EPFB varied widely over the 20 studies. The prevalence ranged from 9% (21) to 78% (32). See also general comment below on superscript of paper reference.

**Response:** Thank you for the comment. The numbers in the bracket are the references, now we revised the referencing based on the journal’s criteria or using [ ].

150 random-effects

**Response:** We have changed according to the comment

157 using a random-effects model

**Response:** We have corrected accordingly.

157 “Effect size” should appear in full in the x-axis label

**Response:** Thank you. We have addressed the comment

158 “twenty studies”, delete used.

**Response:** We have deleted it.

158-159 Standard error of effect size should appear in full on the y-axis

**Response:** Thank you for the suggestion, we corrected it accordingly.

160 The subgroups of the 20 studies should be shown in the PRISMA diagram to ensure completeness and intelligibility

**Response:** Thank you for the suggestion. We sub-grouped the included studies based on the laboratory method used, and we described it in the flow diagram and early in the result section.

170 Pooled proportion of culture-positive EPTB using random-effects model

**Response:** Thank you, we revised it as per the suggestion.

172 Same style as 170

**Response:** We have changed according to the comment

174 Same style as 170

**Response:** We have changed according to the comment

184 Funnel plot of subgroup of 15 of the 20 selected studies

**Response:** We have changed according to the comment

184 Standard error of effect size and effect size should appear in the axis labels

**Response:** Thank you for the valuable comment; we revised accordingly per the suggestion. and effect size according to the comment

186 Same style as 184

**Response:** Thank you, we revised it accordingly.

188 Same style as 184

**Response:** Thank you, we revised it accordingly.

192 A history of PTB infection

**Response:** Thank you, we corrected it.

200 Delete “of”

**Response:** Thank you we have deleted it.

196 HIV should be HIV-1

**Response:** Thank you for the suggestion, we revised it accordingly.

204 A history of PTB infection ("history" implies previous history)

**Response:** We have corrected per the suggestion.

210 … similar to that…

**Response:** We revised it.

214 “systemic” should be “systematic”

**Response:** Thank you, now it is corrected.

217 HIV-1

**Response:** Thank you for the suggestion, we revised it accordingly.

218 …risk factors associated with EPTB infection…

**Response:** Thank you, we revised it as per the suggestion.

219 HIV-1

**Response:** Thank you, now it is revised accordingly.

220 … EPTB patients were HIV-1 infected,…

**Response:** Thank you. We revised as per the suggestion.

223 TB-HIV-1 co-infection

**Response:** Thank you. We revised it accordingly.

227 … with a history of… (“history” implies previous history)

**Response:** We have revised it accordingly.

235 Firstly,

**Response:** Thank you, we revised it.

237 Secondly,

**Response:** Thank you, we revised it.

238 Statistical power and its reduction has not been discussed in the paper. What is the estimate of the reduction and has this invalidated the paper? (Does statistical power here refer to I^2, p-value or items outside the 95% pseudo-confidence gradients in the funnel plots? 238 “poor quality of several studies” – I am not sure this has been sufficiently discussed (if at all)

**Response:** Thank you for the valuable comment. Now, we revised the limitation of the study as follows “In the end, the current study had its limitations. Firstly, the degree of EPTB prevalence in many parts of the country has yet to be addressed, making it impossible to conclude the true burden of EPTB in Ethiopia. Secondly, the observed publication bias that could be due to the differences in the laboratory methods might underestimate the estimated prevalence. Thirdly, there is high heterogeneity among studies that might affect the true estimates. However, the findings are still significant, because the rising rate of EPTB patients in the general population is concerning”

244 HIV-1

**Response:** We revised it accordingly.

General: number references to papers should preferably be shown as superscripts to avoid with number in the paper. For example, line 49, …region (8%) (3) should be …region (8%)3 (3 as superscript)

**Response:** Thank you for the valuable comment and suggestion. Now we revised all the references according to the PloS One guideline, Such that we used the square brackets “[ ]”.

References: Journal references should be italicised. Latinisms like [et.al](http://et.al/). should be italicized

**Response:** Thank you, we revised as per the suggestion.

Figure 1: PRISMA diagram should show the subgroups budding off the “Final analysis” box. Reference to this in the main body of the paper will simplify some of the wording in the main body

**Response:** Thank you for the suggestion, as we have stated in the previous response we revised the flow diagram according to the PRISMA guideline.

Figure 2: Forest plot should be sorted by ES and show ranking from lowest to highest ES. Metaferia et. al. would be at the bottom and Iwnetu [et.al](http://et.al/). at the top

**Response:** Thank you for the comment, we revised it accordingly.

Figure 3: Axis-labels to be described in full

**Response:** We have described in full according to the comment

Figure 4: Sort the publications as in figure 2

**Response:** Thank you, we revised it accordingly.

Figure 5: Sort the publications as in figure 2

**Response:** Thank you, we revised it accordingly.

Figure 6: Sort the publications as in figure 2

**Response:** Thank you, we revised it accordingly.

Figures 7, 8 and 9 – same as in figure 3

**Response:** Thank you, we revised it accordingly.

**Reviewer #2:**

This is a well written article, and findings will be helpful to Ethiopian health care workers, and others working in a LMIC/African context.

However, I have a difficulty with the foundational methodology (meta-analysis) of the study.
o It is not stated what the research questions for the descriptive studies of EPTB which were included in the meta-analysis were.

o Studies which were included could have identified EPTB as a subset of all PTB, or they may have described EPTB alone, from persons with clinical criteria matching only those in whom EPTB is suspected.
o Without specifying which approach the authors of this meta-analysis wanted de novo, the selection criteria results in a wide range of approaches to EPTB diagnosis being selected, which will lead to enormous heterogeneity, and lack of meaningful comparison.

Before conducting a meta-analysis, it is critical to given to formulate the review question clearly. I think the review question ‘to investigate the prevalence of EPTB in Ethiopia’ is too broad. It will be helpful to say ‘Investigate the prevalence of EPTB amongst persons with non-pulmonary TB’. The results are then clearly applicable to persons in whom PTB has been excluded.

As the current aim stands, it implies that that the aim is to identify the burden of TB amongst all TB cases in Ethiopia. This begs the question of a denominator. Assessing the prevalence of EPTB requires that one knows the total burden of TB. How is it possible to assess the prevalence by identifying only studies that described EPTB? These studies would have identified persons who were suspected of EPTB as the starting point. Equally, an ascertainment of risk factors can only be done by comparison with non-EPTB groups.

Without narrowing the research question, it is not meaningful to present or interpret Forest plots.

**Response:** Thank you for the pertinent comments and valuable suggestions. This study is designed to investigate/estimate/ the prevalence of bacteriologically confirmed EPTB among individuals who are presumptive to have EPTB. Thus, the denominator is the number of EPTB or non-respiratory TB presumptive individuals, while the numerator is the number of bacteriologically confirmed EPTB presumptive individuals. Now we described in detail the research questions and the objectives in the revised manuscript. We revised the abstract section, the objectives at the end of the introduction section, and the first paragraph in the discussion section.

Other minor comments

A small consideration re methodology, search strategy – are there articles published by Ethiopians in literature that would not be listed in pubmed, Science Direct or on google scholar?

**Response:** Thank you very much for your informative comments. The majority of the articles published from Ethiopia are open access, because Ethiopia is among the low-income countries which waived article processing charges. Thus, all open access articles can be accessed through Google Scholar research. In addition, Since we cannot freely access other databases in Ethiopia we could not search them. To address this comment we have added a sentence that indicates as our search is not comprehensive in limitation.

Line 49 Extrapulmonary Tuberculosis – ‘tuberculosis’ should be lower case.

**Response:** Thank you for the comment, we revised it accordingly.

Line 64 Turnaround Time – T should be lower case,

**Response:** Thank you, we revised it as per the suggestion.

Line 70 Anti-TB medicine – rather ‘anti-tuberculosis therapy’

**Response:** Thank you, we revised it accordingly.

Line 108 The NewCastle Ottowa quality assessment scale is a checklist for cohort and case control studies. What motivated the use of this scale, over and above the PRISMA guidelines which are sufficient for meta-analyses and systematic reviews.

**Response:** Thank you for the valuable question. This systematic review and meta-analysis were conducted following the PRISMA guidelines. We used the Newcastle-Ottawa quality assessment checklist to assess the quality of individuals studies based on the questions available in the checklist as the Joanna Briggs critical appraisal tool. Since all studies included in the current study are cross-sectional studies we have used the Newcastle-Ottawa quality assessment scale adapted for cross-sectional studies.

Yours, Sincerely

Getu Diriba
