# Supplementary material for: Bacteriologically confirmed extrapulmonary tuberculosis and the associated risk factors among extrapulmonary tuberculosis suspected patients in Ethiopia: A systematic review and meta-analysis
Source: PLoS One. 2022 Nov 23;17(11):e0276701. doi: 10.1371/journal.pone.0276701 (PMC9683558; doi:10.1371/journal.pone.0276701)
Supplement: Attachment — Submitted filename: PONE-D-22-000257__R1_reviewercomments.docx [file pone.0276701.s006.docx]

**PONE-D-22-000257_R1**

**General comments**

1. Bacteriologically-confirmed should be hyphenated everywhere.
2. Please explain the rationale for the study more clearly. Line 78 talks about ‘policy making’ – policy making about what? The question is – how will it benefit policy makers to understand what proportion of EPTB is bacteriologically confirmed? What policy will follow, once policy makers know this information. Follow this logic:

If the following statements are true:

- Registered # (number of) EPTB cases=registered # (number of) bacteriologically confirmed EPTB PLUS registered # (number of) clinically diagnosed EPTB
- True # EPTB cases= #bacteriologically confirmed PLUS (unknown% of) clinically diagnosed EPTB cases

Then:

If most cases of EPTB are diagnosed clinically in Ethiopia because of paucity of diagnostic tests, how will it help to conduct a meta-anaylsis to establish the prevalence of bacteriologically confirmed EPTB cases in Ethiopia? This exercise will be affected by the same diagnostic limitations experienced in Ethiopia. It will not allow policy makers to have an idea of how many EPTB cases are actually caused by TB.

Would it be meaningful to compare % of EPTB cases diagnosed bacteriologically in Ethiopian cohorts with % of EPTB cases diagnosed bacteriologically in non-Ethiopian cohorts in contexts that are epidemiologically comparable with the Ethiopian context but where diagnostic testing is stronger? (if there is such a context?)

3. Rgarding the search strategy – why did you not include the term –‘extra-pulmonary TB’?
4. In the plots of ‘pooled proportions’ –the horizontal axis should range from 0-1. Across the figures, horizontal axes range from -0.5-1.5. This should be corrected. See <https://archpublichealth.biomedcentral.com/articles/10.1186/2049-3258-72-39>
5. The discussion restates the findings (related to the prevalence of bacteriologically-confirmed TB amongst all EPTB) but then proceeds to make comparisons with the proportion of EPTB cases amongst all TB cases. The narrative then discusses risk factors for extra-pulmonary TB vs PTB. AS such, the discussion does not concur with the aim of the paper, nor highlight what the reasons are for such a low % of EPTB cases having bacteriological confirmation.

**Introduction**

Line 39 – were found to be independently associated with bacteriologically confirmed EPTB?

If cases of EPTB were not bacteriologically confirmed, how were they diagnosed?

Line 75 – it’s not clear who the ‘they’ is (patients? Health policy makers?), and what the sentence means ‘These people were misdiagnosed or received therapy too late, and they overestimated the scale of the problem at the community level

Line 76 – does not make sense ‘There is a scarcity of data on the bacteriologically confirmed of EPTB among suspected EPTB individuals in Ethiopia’

Line 77 – avoid ‘isn’t’ – rather say ‘ there is no comprehensive review….

**Materials and methods**

Line 85 ‘gray’ should be ‘grey’

Line 107 – remove ‘a’ in ‘is a bacteriological confirmation’

Line 107 remove ‘an’ and ‘infection’. Sentence can read ‘is bacteriological confirmation of EPTB’.

Line 121 – should it not read ‘the pooled prevalence of *bacteriologically confirmed* EPTB’ ?

Line 123 – if I understand your aim correctly, line 123 should read ‘…was used to determine the pooled prevalence of bacteriologically confirmed EPTB in all patients with EPTB’?

**Results**

Line 121 and PRISMA flow diagram. It’s not clear what is meant by exclusion of studies because of ‘difference outcomes’. The box in the PRISMA diagram also reads ‘no full text or abstract only, and ‘difference outcome’ – what is meant by ‘difference outcome’?

Line 133 – ‘due to this reason’ – should read ‘because of this, 23 articles were removed’. However, is is not clear what the problems with these studies were – what was it abou the subjects, design or quality that made them unsuitable?

Line 138 ‘Smear’ should have lower case s

Line 142 if this is the caption for Figure 1, it should be a full sentence, and refer to the context of the study. Eg. ‘A PRISMA flow diagram depicting screening an selection process to identify literature describing extra-pulmonary TB cases in Ethiopia, 200x-200y.

Line 154 – remove ‘method’ so it reads ‘two studies applied smear microscopy’.

Line 156 as above remove ‘method

Line 157 as above remove ‘method

Line 158. If this is the legend for the table, it should reference the context of the study e.g. General characteristics of studies describing extrapulmonary tuberculosis in Ethiopia, 200x-y.

Table 1 – Header row –

- The column ‘Sample size’ should read ‘Sample size (N)’.
- the column ‘Positive’ should read ‘number of cases with bacteriological confirmation (n/N, %)’. This column could also have a % in brackets
- The meaning of the column header ‘associated factors’ is not clear. Perhaps the authors mean ‘risk factors’ – but then risk factors for EPTB or risk factors for bacteriologically confirmed TB? The column header should read ‘risk factors for [xx]’

Table 1 – column entitled ‘Positive’

- Each number should be followed by a % so for row 1, it should read ‘107 (73)’, for row 2 should read ‘117 (78) etc

Table 1 column enetitled ‘Associated factors’

- Where there are none, rather than blank the column should say ‘not stated’.

Row 166 – it is not clear what is meant by ‘the variety of distinct EPTB’.

Row 167 -169– the sentence beginning ‘The graph depicted studies’ – is an interpretation and should be placed in the Discussion section

Row 170. If this is the legend for Figure 2, it is not a full sentence and does not reference the context of the study. It is not clear what ‘any bacteriological positive of EPTB’ means. I think this is a grammatical issue. Perhaps the authors mean ‘the pooed proportion of bacteriologically-confirmed EPTB cases amongst alstudies identified for review’

Row 172 – as above.

Row 173 – the subgroup analysis should be described in the methods section. So rows 174-176 should be moved to Methods, under ‘statistical analysis’

Row 174 – what are ‘EPTB positive cases’ – I think authors mean ‘bacteriologically confirmed EPTB cases’? if so, rather state this.

Row 177 – The meaning of the phrase ‘performed by a diagnostic test’ – is unclear. Does this refer to a ‘diagnostic test’ which is part of the meta-analysis statistical evaluation, or ‘a diagnostic test’ for TB (ie culture, etc). The sentence makes sense without this phrase.

Row 178 – the words ‘however’ and ‘still’ should be omitted as these imply some kind of evaluation/comparision/judgement, which is rightly part of the discussion, and should not appear in the ‘results’ section.

Row 178 - the word ‘In’ should be substituted with ‘For’, to read ‘For culture, smear microscopy….’

Row 183. If this is the legend for Figure 4, it should include reference to the context of the study. The bracket at the end should be removed.

Row 188-190 – this is a description of methods and should be included in the methods section.

Rows 195-197 – Legends should refer to context, and should describe what the funnel plot is attempting to describe.

Row 203 the sentence should read ‘Additionally, being HIV-positive….etc

Row 204 – change ‘Urban’ to a lower-case ‘u’.

Row 205 – what is a ‘remark category’? The meaning of this sentence is unclear.

**FIGURES**

These should all be contextualised as above.

Discussion

Line 218 ‘ it is not meaningful to compare the ‘prevalence of bacteriologically-confirmed EPTB ’ (line 216), with the prevalence of EPTB amongst all cases of TB in africa.

- The former = [bacteriologically confirmed EPTB] / [all EPTB cases ] (this is what the study aimed to do)
- The latter = [all EPTB cases] / [all TB cases] (this is reference 7 and 36]

Line 221-223 – same problem as above. The authors are not comparing the same prevalence.

The discussion focuses on risk factors for EPTB, but its not clear if these are risk factors for bacteriologically-confirmed TB amongst all EPTB, or ‘risk factors for EPTB vs PTB’. This relates back to the comment on the ‘Associated factor’ column in Table 1.
