# Supplementary material for: Bacteriologically confirmed extrapulmonary tuberculosis and the associated risk factors among extrapulmonary tuberculosis suspected patients in Ethiopia: A systematic review and meta-analysis
Source: PLoS One. 2022 Nov 23;17(11):e0276701. doi: 10.1371/journal.pone.0276701 (PMC9683558; doi:10.1371/journal.pone.0276701)
Supplement: Attachment — Submitted filename: Response to Reviewers.docx [file pone.0276701.s008.docx]

**Ref: PONE-D-22-00257R1**

**Bacteriologically confirmed extrapulmonary tuberculosis and the associated risk factors among extrapulmonary tuberculosis suspected patients in Ethiopia: A systematic review and meta-analysis**

Dear Editor,

Thank you very much for your informative comments on our manuscript entitled “Bacteriologically confirmed extrapulmonary tuberculosis and the associated risk factors among extrapulmonary tuberculosis suspected patients in Ethiopia: A systematic review and meta-analysis”. We have addressed your comments one by one. We also appreciate you for allowing us to revise our manuscript and correct errors in the previous version. We thank the reviewers for their informative comments, and our point-by-point responses to the reviewers’ comments are given below. Also, we would like to inform you that we have used track changes to indicate where we made changes in response to the reviewers’ comments. Review Comments to the Author

Reviewer #1:

 1 Reviewer 1 comments in the first review have been addressed and the original document has been extensively revised or rewritten. As a result, the revised document is more readable. The revised document has however highlighted additional significant matters and some minor issues. In view of the novelty and importance of the study, it is well worthwhile to resolve these matters and issues.
2 Lines refer to the revised document.
3 Lines 29, 33, 36, 37,117,163,164,180,181,182, and 212. The I^2 test for heterogeneity has values of 98.45%, 98.56%, 98.73% and 96.43%. In terms of Cochran’s Q statistic I^2=((Q-df)/Q*100% (see Cochrane Handbook, 2011), heterogeneity greater than 75% means that studies in the forest plot are not sufficiently comparable and that a meta-analysis may be invalid. A possible remedy is to ignore heterogeneity and adopt a fixed effects model.
4 Lines 170, 183, 184, 185 and Figures 4, 5, and 6. In terms of point 3 (for a I^2 statistic > 75%) a fixed effects model is usually applied unless a suitable analytical justification is given for the application of a random effects model (see Cochrane Handbook, 2011).
5 Lines 116 and 117, a rule of thumb for heterogeneity of I^2 of 50% is moderate and I^2 ≥ 75% is high – see point 3 above. The unqualified statement in line 117 of I^2 ≥ 50% would seem to imply that high heterogeneity is desirable.
6 Lines 180 to 182. P-values of <0.01 are given. P-values as given are incorrectly associated with I^2 tests, which are applied as broad-based categories (see Cochrane Handbook, 2011). P-values are associated with the alternative chi-square test in which a p-value of <0.01 is indicative of a chi-square value in excess of the table value, which indicates high heterogeneity in values and like a high I^2 value (75%), requires further analysis to justify the use of a forest plot (see above).

**Response:** Thank you very much for your unreserved constructive comments. These sections of comments (1-6) have been updated to follow a fixed-effects model, as requested.

7 Repetition of statistics in 5 places - see lines in point 3 above. This suggests that the document requires editing.

**Response:** Thank you for the valuable comment. We have addressed it.
8 Inconsistency in presentation of statistics. The repetitions of statistics in point 7 are inconsistent. Lines 180 to 182, 164 and 212 give p-values, which are not given in the other lines in point 3 above.

**Response:** Thank you for the valuable comment. We have corrected it.
9 The forest plots in Figures 4, 5 and 6 in support of the data in point 3 above have I^2 values >75% which, with p-values <0.01 (presumably relating to a chi-square test), which indicates high heterogeneity. The plots do not indicate if a random effects model or a fixed effects model have been applied. An analysis justifying the relevance of forest plots is required in light of high heterogeneity.

**Response:** Thank you for the valuable comment. We have used the fixed-effect model and put the type of the model of analysis.
10 Lines 35 to 37 which refer to smear microscopy, Xpert MTB/RIF, culture as separate categories with statistics for each, does not agree with line 106 which adds

nucleic acid amplification as an additional test.

**Response:** Thank you for your suggestion. We are removed additional words.
11 Lines 35 to 37, tests do not agree with the categories in lines 136 to 138, which combine categories. For example, in line 136, three studies combined culture, Xpert MTB/RIF and smear microscopy. The same combination of categories in lines 152 to 157 is at odds with the separate categories in lines 35 to 37. Proposed categories do not agree with tested categories and evaluated categories.

**Response:** Thank you for your comments. Our findings are reported on lines 35–37 and the characteristics of the research included in the review are described on lines 152–157. We have omitted the repeated lines 136–138.

12 Lines 32, 34, 163, 174, 180-182, 211, 216 do not explain how pooled estimates were computed. Is this a weighted average by subject or by study? How are the 95% confidence intervals computed, are they also weighted by subject or by study?

**Response:** Thank you for your critical observations and comments; the pooled estimate was weighted by study.

13 Line 202 risk factors are live animals, raw milk, HIV, male, less income, urban. Line 215 adds contact with EPTB patients and line 258 adds underlying disease as a risk factor. Line 75 risk of misdiagnosis of tuberculosis is also a risk factor as is line 66 – resource limited settings. Line 243 refers to women with lymphadenitis with a higher rate of EPTB than men and Line 245 as women with a higher rate of EPTB than men contrary to line 202 which regards maleness as being an outright risk.

**Response:** Thank you for your critical observation and comment; we revised as per the suggestion.

14 Lines 256 to 259, in the conclusion, leaves out male, live animals, raw milk, lymphadenitis, and misdiagnosis as risk factors without justification. Consistency in the set of risk factors, from proposal to testing and conclusion should be maintained. Adding or dropping risk factors has not been explained or justified

**Response:** Thank you for your critical observation and comment; we have put the most reported risk factors in the conclusion.

15 ‘Previous history’ in lines 37, 41, 212, 237, 239, 256, 258 should be ‘history’.

**Response:** Thank you for the suggestion. We revised it.

16 /EPTB/ in lines 25 and 80 should be omitted.

**Response:** Thank you for your suggestion. We have deleted it.

17 Line 45 capitalise f in factors.

**Response:** Thank you for your suggestion. We have corrected it.

18 Line 99 space between 2010 and worksheet.

**Response:** Thank you for your suggestion. We have corrected it.

19 Line 120 …software (STATA) so that line 122 refers to ‘STATA’.

**Response:** Thank you for your suggestion. We corrected it accordingly.

Reviewer #2: The authors have clarified the aim of the study, and it is now possible to make comments on the rest of the paper. Please see detailed comments in word document which are summarised here.
Firstly, the rationale for the study could be made clearer.

**Response:** Thank you for your suggestion. We have revised the rational of the study.

Secondly, in table 1, it is not clear what 'associated factors' refer to. I think the authors mean 'risk factors', but it is not clear if the factors listed are 'risk factors for EPTB (as opposed to PTB) or 'risk factors for bacteriologically-confirmed TB (vs non-bacteriologically confirmed TB). Following on from this, in the discussion, the authors have made incorrect comparisons with international iterature, and have focused the discussion on prevalence of EPTB amongst all TB cases. Therefore the discussion does not have bearing on the aim of the paper (to discuss % of EPTB that is bacteriologically confirmed, as opposed to the % which is not bacteriologically confirmed). The authors point out that diagnostic tests in Ethiopia are limited, and that understanding the % of EPTB that is bacteriologically confirmed will guide policy. It's not clear in what direction this policy should go. This could be (should be?) brought up in the discussion.

**Response:** Thank you for your suggestion. We have revised it accordingly.

Thirdly, correction of grammatical/syntax errors will make aspects of the paper easier to understand.

**Response:** Thank you for your suggestion. In the revised manuscript we have addressed the grammatical problems.

Fourthly, all legends to figures and tables should refer to the context of the figure/table in the paper.

**Response:** Thank you for your suggestion. We have corrected all the legends accordingly.

Fifthly, once the above are corrected, a statistical reviewer should assess the validity of the tests that have been conducted and how they have been interpreted.

**Response:** Thank you for your suggestion. Every statistical analysis is correct.
