# Supplementary material for: Bacteriologically confirmed extrapulmonary tuberculosis and the associated risk factors among extrapulmonary tuberculosis suspected patients in Ethiopia: A systematic review and meta-analysis
Source: PLoS One. 2022 Nov 23;17(11):e0276701. doi: 10.1371/journal.pone.0276701 (PMC9683558; doi:10.1371/journal.pone.0276701)
Supplement: Attachment — Submitted filename: Response to Reviewers.docx [file pone.0276701.s009.docx]

**Ref: PONE-D-22-00257R2**

**Bacteriologically confirmed extrapulmonary tuberculosis and the associated risk factors among extrapulmonary tuberculosis suspected patients in Ethiopia: A systematic review and meta-analysis**

**Dear Editor,**

Regarding our manuscript, "Bacteriologically confirmed extrapulmonary tuberculosis and the associated risk factors among extrapulmonary tuberculosis suspected patients in Ethiopia: A systematic review and meta-analysis" we appreciate your insightful comments. Each of your comments has been addressed one by one. We also appreciate your letting us modify our manuscript and make corrections in the first draft. We appreciate the reviewers' informative comments and suggestions. His comments have improved the manuscript effectively. Also, we would like to inform you that we have used hyperlink to indicate where we made changes in the previous version of the manuscript based on the reviewers’ comments.

Reviewer #1

1 Please insert spaces or remove spaces as indicated in the red or blue underlines on the document

**Response:** Thank you for the valuable comment. We have addressed it.

2 line 117 "the presence of publication bias..." is a repetition of the previous sentence

**Response:** Thank you for the valuable comment. We have removed the repetition sntence.

3 line 124 remove STATA 14... details, this has already been defined in line 121

**Response:** Thank you for your critical observation and comment; we have addressed.

4 line 171 bacteriologically

**Response:** Thank you for your suggestion. We have corrected it.

5 line 121 remove (STATA)

**Response:** Thank you for your suggestion. We have remove it.

6 line 100 facility-based

**Response:** Thank you for your suggestion. We have corrected it.

7 line 215 meta-analysis

**Response:** Thank you for your suggestion. We have corrected it.

8 line 169 Figure 2: pooled not pooed

**Response:** Thank you for your suggestion. We have corrected the word.

9 Table 1 last column is 12 point type, the preceding columns are point 8

**Response:** Thank you for the valuable comment. We have addressed in the revised manuscript.

10 The document needs a final edit for typos and set-out

**Response:** Thank you for the valuable comment. We have addressed in the revised manuscript.

Kind regards,

Getu Diriba

Ethiopian Public Health Institute

Phone: +251913828019. Fax: +2510112780431

P.O.Box 1242, Addis Ababa, Ethiopia

E-mail: [getud2020@gmail.com](mailto:getud2020@gmail.com)
